# Stereoselectively synthesis and structural confirmation of dehydrodipeptides with dehydrobutyrine

**DOI:** 10.1186/s40064-016-2005-z

**Published:** 2016-04-01

**Authors:** Xia Tian, Linna Li, Jianrong Han, Xiaoli Zhen, Shouxin Liu

**Affiliations:** College of Sciences, Hebei University of Science and Technology, Shijiazhuang, 050018 People’s Republic of China; State Key Laboratory Breeding Base-Hebei Province Key Laboratory of Molecular Chemistry for Drug, Hebei University of Science and Technology, Shijiazhuang, 050018 People’s Republic of China

**Keywords:** l-threonine, α,β-Dehydrobutyrine, Z-isomer, Dipeptide, Synthesis

## Abstract

Most of polypeptides containing α,β-dehydroamino acids have important biological activity, so exploration of synthetic method has practical significance. In this paper, dipeptides were prepared from l-threonine by protecting of c-terminal allyl acetate, and condensing reaction with a series of N-Boc amino acid. Then, treatment of dipeptides obtained with DMAP, (Boc)_2_O and tetramethylguanidine in the acetonitrile occured β-elimination reaction to yield stereoselectively dehydrodipeptides. Structures of dehydrodipeptides were confirmed by ^1^H NMR, ^13^C NMR and MS. Analysis of ^1^H NMR, 2D NMR and crystal structure showed that the dehydrodipeptides were Z-configuration.

**Electronic supplementary material:**

The online version of this article (doi:10.1186/s40064-016-2005-z) contains supplementary material, which is available to authorized users.

**Graphical abstract:**
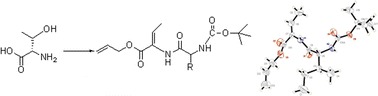
Dehydrodipeptides were prepared from l-threonine. Their structures were confirmed by ^1^H NMR, ^13^C NMR and MS

## Background

α,β-Dehydroamino acids, as unnatural amino acids, are found in many natural products including the fungal metabolites, β-lactam antibiotic, sulfide antibiotics, anticarcinoma antibiotics, phytotoxin, the antrimycins, tentoxin, and the phosphatase inhibitors microcystin, nodularin and the synthetic drugs (Gross and Meienhofer 1983; Valentekovich and Schreiber 1995; Botes et al. 1984). In the active peptide, α,β-dehydroamino acids are usually used to fixed peptide main chain and side chain, and make its conformation keeping relatively stable, also can inhibit its biological degradation process. So α,β-dehydroamino acids play an important role in the design and synthesis of biological peptide and the study of its structure–activity relationship (Chang et al. 1998; Rappoport 1994; Harburn et al. 1998; Kohno et al. 1996; Bierbaum 1999).

Many methods available for synthesis of α,β-dehydroamino acids have been reported (Bonauer et al. 2006; Poisel and Schmidt 1976; Kolasa 1983; Maekawa et al. 2004; Schmidt et al. 1992; Trost and Dake 1997; Nagano and Kinoshita 2000; Chen et al. 2005; Goodall and Parsons 1995; Li et al. 1996; Somekh and Shanzer 1983; Miller 1980; Stohimeyer et al. 1999). However, these methods often are low yielding, multistep, require tedious purification steps to remove reagent side products, or incorporate unusual, difficultly obtained amino acid intermediates. Elimination of water from β-hydroxy-α-amino acids is a well-established route to obtain β-dehydroamino acids. This method has been used for the preparation of dehydroalanine and dehydroaminobutanoate from serine and threonine. Shioiri demonstrated Martin’s sulfurane is a mild, neutral and stereospecific dehydrative agent for the dehydrative elimination, which give stereospecific polypeptide with Z-configurational unsaturated amino acid, through removing hydroxyl group of β-hydroxy-α-amino acids in dipeptide or tripeptide (Yokokawa and Shioiri 2002). However, the disadvantage is that the Martin’s sulfurane is expensive. Ferreira reported one important and well-used approach involves the β-elimination reactions of serine and threonine derivatives with Boc-anhydride and 4- (*N*,*N*-dimethylamino) pyridine (DMAP) (Ferreira et al. 1999; Ferreira et al. 1998). Furthermore, they use the base *N*,*N*,*N*,*N*-tetra-methylguanidine (TMG) to induce elimination of the *tert*-butyl carbonate group from the *O*- (*tert* butyl-oxycarbonyl)-β-hydroxyamino acid derivatives, give the corresponding dehydroamino acid derivative. This two-step method can be carried out as a one-pot procedure and is stereoselective, giving only the *Z* isomer (Ferreira et al. 2007). In this study, We now wish to synthesize a variety of dehydroamino acid derivatives by Ferreira’s synthetic approach and the double bond formed by dehydration reaction is determined Z isomer by NOESY (Shimohigashi et al. 1982; Duhamel et al. 1972) and X-ray crystal diffraction.

## Result and discussion

### Synthesis

In the synthesis of dehydrodipeptides, first, we obtained dehydroamino acids using *N*-protected β-hydroxyamino acid esters as raw material. Then, we attempted to obtain dehydrodipeptides by condensation reaction of dehydroamino acids and amino acid. However, owing to the low reactivity of the α-amine group of dehydroamino acids and to the instability of its *N*-deprotected derivatives, byproducts of reaction are very much, and difficult to separate by this methods. Therefore, this led us to investigate the applicability of Ferreira’s methodology to the dehydration of peptides containing β-hydroxyamino acids as precursors of dehydropeptides. First, dipeptides were prepared from two amino acid, then dehydrodipeptides were obtained by β elimination dehydration of dipeptides.

In order to prepare dipeptides containing l-threonine, the protection of functional groups were very important because of containing three activity groups in l-threonine. Generally, protected groups were selected according to the final products. In this studies, allyl group were used to protect carboxy terminus of amino acid. However, if l-threonine was treated with allyl bromide in dry DMF, besides carboxyl group, the amino group was reacted with allyl bromide. Thus, amino group of l-threonine was protected firstly by Boc group, When *N*-Boc-protected l-threonine **2** was reacted with allyl bromide in the presence of K_2_CO_3_ in dry DMF, it was converted quantitatively into **3** within 12 h. However, in the presence of NaOH, only traces of **3** were detected. This suggests that the by-product may result from a base-induced side-reaction. Thus, the use of an alternative base could possibly reduce this side reaction, so K_2_CO_3_ was substituted for NaOH in the reaction of **2** with allyl bromide. Therefore, *N*-Boc-protected l-threonine **2** were treated with 1.2 equiv. of allyl bromide and K_2_CO_3_ in dry DMF to give the corresponding allyl esters of *N*-protected l-threonine **3**.

**Scheme 1:**
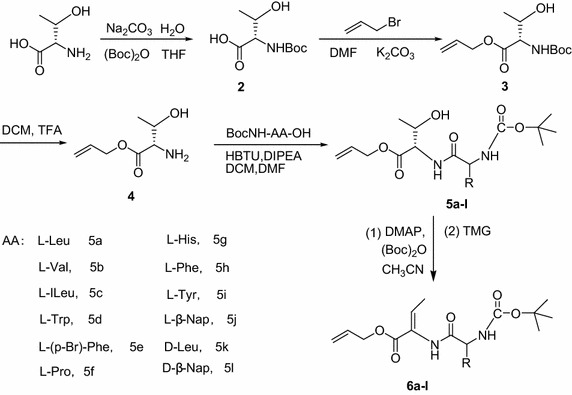
The synthetic route of dehydrodipeptides

Dipeptides were readily prepared from **3** by N-deprotection and coupling with the N-protected amino acid using DIPEA/HBTU in higher yield (Scheme 1). Compound **4** was treated with 1.2 molar amounts of N-protected amino acid in the presence of 1.2 molar amounts of HBTU and DIPEA in dichloromethane (DCM) and DMF at room temperature to afford the desired dipeptides **5a-5l** in high yield (>90 %).

Ferreira’s method, one of the noteworthy stereospecific feature of the method was reported for the elimination with Boc-anhydride and 4- (*N*,*N*-dimethylamino) pyridine (DMAP) and TMG by Ferreira et al. (1999). Therefore, We treated N-Boc protected dipeptides with 3.3 equiv. of (Boc)_2_O in the presence of DMAP, gave *O*-*tert*-butyl carbonates of dipeptides, followed by direct reaction with *N*,*N*,*N*,*N*-tetra-methylguanidin (TMG) without isolation, afforded the corresponding dehydrodipeptides in good yields. This two-step method can be carried out as a one-pot procedure. However, In order to compare with Ferreira’s method, we tried to synthesize dehydrodipeptides by β-elimination of dipeptides containing β-hydroxyl group using Martin’s sulfurane. Unfortunately, the treatment of dipeptides **5** with Martin’s sulfurane did not led to the desired products. Therefore, Ferreira’s synthetic approach could be used efficiently stereoselective synthesis of dehydrodipeptides. In comparison with previous methods, this procedure is a one-step process, since separation of the intermediates is unnecessary and theatment of intermediates with TMG gave the corresponding dehydrodipeptides in good yield. This mildness of the mothod is compatible with the presence of a variety of functional groups.

### Structure characterization

**Fig. 1:**
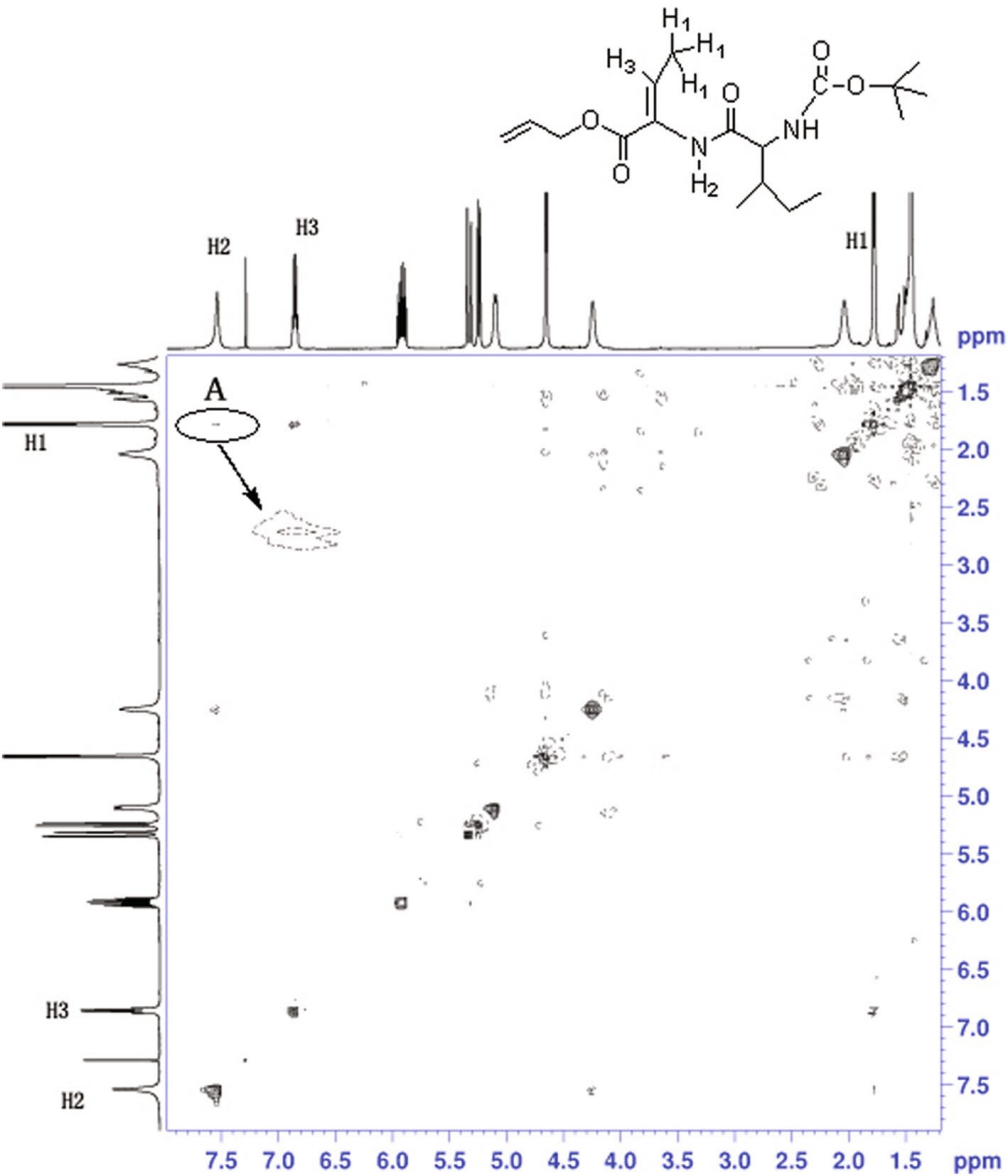
1H NOESY spectrum of **6c** in CDCl3 at 293.2 K. Schematic representation of the polypyridyl bases and complexes **1** and **2**

**Fig. 2:**
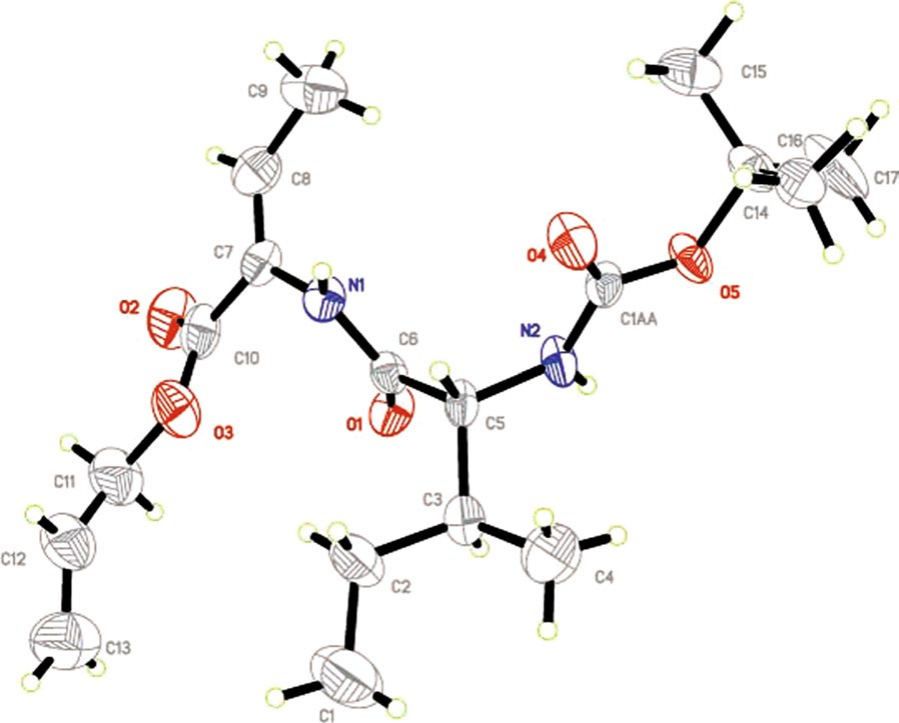
The X-ray crystal structure of **6c**

To further investigate the configuration of the dehydrodipeptides, **6c** was subjected to the 2D NMR measurements. Since the NOE cross-peaks between the protons that are closer than 0.4 nm in space will be observed in NOESY spectrum and the relative intensities of these cross-peaks depend on the spaces between the corresponding protons. As can be seen from Fig. 1, the NOESY spectra of 6c showed clear NOE cross-peaks A of H1 of methyl group in double bonds and H2 of amino group in amide bonds, demonstrating that the substituents of methyl goup and amino group located on the same side of the double bond. As well as no correlation between H3 of double hand and H2 of amino group, would provide us further information about the orientation of the H proton and amino group in the double bonds. These indicated distinctly that the double bond in dehydrodipeptides was Z configuration. In addition, We obtained single crystals suitable for X-ray crystallography by slowly evaporating a ethyl acetate solution of **6c**. Interestingly, X-ray crystallographic analysis of **6c** reveals that the double bond C7–C8 in dehydrodipeptides was Z configuration (Fig. 2). Therefore, the dehydro-dipeptides was synthesized stereoselectively by β-elimination reaction using DMAP, (Boc)_2_O and tetramethylguanidine.

## Experimental

### Materials and instrumentation

All of the org. solvents used in this study were dried over appropriate drying agents and distilled prior to use. All analytical grade chemicals were purchased commercially and used without further purification. Compound **2** (Goodall and Parsons 1995) was prepared according to the literature procedures.

^1^H NMR and ^13^C NMR spectra were recorded on a Bruker AVANCE II500 instrument in CDCl_3_ solution, using tetramethylsilane as an internal reference. Elemental analyses were performed on a Perkin-Elmer 2400C instrument. The X-ray diffraction data were collected by using a Rigaku Mercury CCD AFC10 system with monochromated Mo Ka radiation.

### Synthesis

#### *Boc*-*L*-*Thr*-*OAllyl* (**3**)

21.9 g Boc-l-threonine**-**OH (0.1 mol) was dissolved in 70 mL DMF, 16.6 g (0.12 mol) K_2_CO_3_ was added and cooled to 0 °C in an ice bath. 14.4 g (0.12 mol) Allyl bromide was added dropwise with stirring by means of a separatory funnel. After the mixture was stirred for 1 h, the two phase solution was allowed to warm slowly to room temperature with vigorous stirring over 12 h. The solid residue was isolated by filtration. The solvent in the filtrate was removed *in vacuo* and the residue was taken up in 80 mL of a saturated NaCl solution, and the aqueous solution was extracted with ethyl acetate (30 mL × 5). The organics were combined and washed with 1 M KHSO_4_ solution, water, saturated KHCO_3_ and saturated NaCl, dried over MgSO_4_, the solvent removed under reduced pressure, evaporated in vacuo to give a viscous liquid, which was purified by means of silica-gel chromatography (Petroleum ether/Ethyl acetate = 5:1) to give Boc-L-Thr-OAllyl **3**, the white solid with yield 91 %. ^1^H NMR (500 MHz, CDCl_3_) 1.26 (d, 3H), 1.45 (s, 9H), 2.89 (br, 1H), 4.26 (d, J = 8.5 Hz, 1H), 4.33 (m, 1H), 4.66 (m, 2H), 5.29 (dd, J = 1.5 Hz, *J* = 10.5 Hz, 1H), 5.37 (d, *J* = 1.5 Hz, J = 16 Hz 1H), 5.49 (d, J = 9.0, 1H), 5.91 (m, 1H). Anal. calc. for C_12_H_21_NO_5_ (259.14): C 55.58, H 8.16, N 5.40; found: C 55.63, H 8.19, N5.44.

#### *Boc*-*N*-*AA*-*L*-*Thr*-*OAllyl* (**5**)

1.29 g (5 mmol) Boc-L-Thr- OAllyl was taken up into a solution of trifluoroacetic acid (20 mL) in DCM (30 mL) and stirred for 3 h to remove the Boc group. After removing the solvent and TFA *in vacuo*, the crude product **4** was obtained (yield > 90 %), which was used to next step reaction without purification. The resulting compound was dissolved in 150 mL DCM and 15 mL DMF and 6 mmol BocNH-AA-OH and 2.25 g (6 mmol) HBTU were added, followed by gradual addition of 7.5 mL DIPEA, the mixture was stirred at room temperature overnight. The solution was concentrated *in vacuo*, then 100 mL water were added, and the aqueous solution was extracted with ethyl acetate (30 mL × 5). The organics were combined and washed with water, 5 % K_2_CO_3_, 2 % HCl and water, dried over MgSO_4_, the solvent removed under reduced pressure, evaporated *in vacuo*, the crude product was purified by means of silica-gel chromatography (Petroleum ether/Ethyl acetate = 5:1) to give white solids.

#### *Boc*-*L*-*Leu*-*L*-*Thr*-*OAllyl* (**5a**)

White solids, yield 95 %. ^1^H NMR (500 MHz, CDCl_3_): δ = 0.93 (d, J = 6.5, 3H), 0.95 (d, J = 6.0 Hz, 3H), 1.21 (d, J = 6.5 Hz, 3H), 1.43 (s, 9H), 1.49 (m, 1H), 1.66 (m, 2H), 3.41 (br, 1H), 4.17 (m, 1H), 4.37 (m, 2H), 4.65 (m, 2H), 5.17 (d, 1H), 5.24 (d, *J* = 10.5 Hz, 1H), 5.34 (d, J = 17.0 Hz, 1H), 5.90 (m, 1 H), 7.05 (m, 1 H). Anal. calc. for C_18_H_32_N_2_O_6_ (372.23): C 58.05, H 8.66, N 7.52; found: C58.01, H 8.63, N7.54 (see Additional file 1: Figure S1).

#### *Boc*-*L*-*Val*-*L*-*Thr*-*OAllyl* (**5b**)

White solids, yield 97 %. ^1^HNMR (500 MHz, CDCl_3_) δ = 0.96 (d, J = 6.5, 3H), 0.98 (d, J = 7.0 Hz, 3H), 1.21 (d, J = 6.0 Hz, 3H), 1.43 (s, 9H), 2.07 (m, 1H), 3.46 (br, 1H), 3.95 (t, 1H), 4.38 (m, 2H), 4.64 (d, 1H), 4.66 (m, 2H), 5.24 (d, *J* = 10.5 Hz, 1H), 5.34 (d, J = 17.0 Hz, 1H), 5.90 (m, 1 H), 6.99 (m, 1 H). Anal. calc. for C_17_H_30_N_2_O_6_ (358.21): C 56.97, H 8.44, N 7.82; found: C56.95, H 8.47, N7.88 (see Additional file 1: Figure S5).

#### *Boc*-*L*-*ILeu*-*L*-*Thr*-*OAllyl* (**5c**)

White solids, yield 93 %. ^1^HNMR (500 MHz, CDCl_3_) δ = 0.89 (d, J = 7.0, 3H), 0.95 (t, J = 7.0 Hz, 3H), 1.23 (d, J = 6.5 Hz, 3H), 1.23 (m, 2H), 1.43 (s, 9H), 1.49 (m, 1H), 2.05 (m, 1H), 2.14 (br, 1H), 4.19 (m, 1H), 4.38 (m, 2H), 4.62 (m, 1H), 4.67 (m, 1H), 4.94 (d, 1H), 5.26 (d, *J* = 10.5 Hz, 1H), 5.34 (d, J = 17.0 Hz 1H), 5.90 (m, 1 H), 6.83 (m, 1H). Anal. calc. for C_18_H_32_N_2_O_6_ (372.23): C 58.05, H 8.66, N 7.52; found: C58.08, H 8.64, N7.57 (see Additional file 1: Figure S9).

#### *Boc*-*L*-*Trp*-*L*-*Thr*-*OAllyl* (**5d**)

White solids, yield 91 %. ^1^HNMR (500 MHz, CDCl_3_) δ = 0.99 (d, J = 6.5 Hz, 3H), 1.31 (s, 9H), 2.39 (br, 1H), 3.15 (m, 2H), 3.30 (br, 1H), 4.15 (m, 1H), 4.45 (m, 2H), 4.47 (m, 1H), 5.12 (d, *J* = 10.5 Hz, 1H), 5.19 (d, *J* = 17.0 Hz, 1H), 5.30 (d, J = 8.0 Hz, 1H), 5.74 (m, 1 H), 6.88 (d, *J* = 9.0 Hz, 1 H). 6.99 (m, 2H), 7.07 (m, 1H), 7.21 (d, J = 8.0 Hz, 1H), 7.52 (d, J = 8.0 Hz, 1H), 8.47 (s, 1H); Anal. calc. for C_23_H_31_N_3_O_6_ (445.22): C 62.01, H 7.01, N 9.43; found: C61.98, H 7.06, N9.44 (see Additional file 1: Figure S13).

#### *Boc*-*L*- *(p*-*Br)*-*Phe*-*L*-*Thr*-*OAllyl* (**5e**)

White solids, yield 94 %. ^1^HNMR (500 MHz, CDCl_3_) δ = 1.17 (d, J = 6.0 Hz, 3H), 1.39 (s, 9H), 2.09 (br, 1H), 2.95 (m, 1H), 3.09 (m, 1H), 3.14 (br, 1H), 4.32 (m, 1H), 4.41 (m, 1H), 4.60 (m, 1H), 4.65 (m, 2H), 5.23 (m, 1H), 5.26 (d, *J* = 10.5 Hz, 1H), 5.33 (d, *J* = 17.0 Hz, 1H), 5.88 (m, 1 H), 6.97 (d, J = 9.0 Hz, 1H), 7.09 (d, *J* = 8.0 Hz, 2H), 7.39 (d, *J* = 8.0 Hz, 2H). C_21_H_29_N_2_O_6_Br (484.12): C 51.97, H 6.02, N 5.77; found: C51.93, H 6.08, N5.81 (see Additional file 1: Figure S17).

#### *Boc-L-Pro-L-Thr-OAllyl* (**5f**)

White solids, yield 90 %. ^1^HNMR (500 MHz, CDCl_3_) δ = 1.20 (d, J = 6.5 Hz, 3H), 1.45 (s, 9H), 1.89 (m, 1H), 2.01 (m, 1H), 2.20 (m, 2H), 3.38 (m, 1H), 3.48 (m, 2H), 4.30 (m, 1H), 4.31 (m, 1H), 4.59 (m, 1H), 4.66 (m, 2H), 5.25 (d, J = 10.5 Hz, 1H), 5.34 (d, J = 17.0 Hz, 1H), 5.90 (m, 1H), 7.24 (br, 1H), Anal. calc. for C_17_H_28_N_2_O_6_ (356.19): C 57.29, H 7.92, N 7.86; found: C 57.31, H 7.91, N7.89 (see Additional file 1: Figure S21).

#### *Boc*-*L*-*His*-*L*-*Thr*-*OAllyl* (**5g**)

White solids, yield 91 %. ^1^HNMR (500 MHz, CDCl_3_) δ = 1.14 (d, J = 6.0 Hz, 3H), 1.42 (s, 9H), 1.60 (s, 9H), 2.99 (dd, J = 6.0 Hz, J = 9.0 Hz, 1H), 3.06 (dd, J = 6.0 Hz, J = 9.0 Hz, 1H), 4.29 (m, 1H), 4.53 (m, 1H), 4.56 (m, 2H), 4.62 (m, 2H), 5.23 (d, J = 10.5 Hz, 1H), 5.31 (d, J = 17.0 Hz, 1H), 5.88 (m, 2H), 7.19 (s, 1H), 8.03 (s, 1H), Anal. calc. for C_23_H_36_N_4_O_8_ (496.25): C 55.63, H 7.31, N 11.28; found: C 55.60, H 7.35, N11.30 (see Additional file 1: Figure S25).

#### *Boc*-*L*-*Phe*-*L*-*Thr*-*OAllyl* (**5h**)

White solids, yield 96 %. ^1^HNMR (500 MHz, CDCl_3_) δ = 1.17 (d, J = 6.5 Hz, 3H), 1.40 (s, 9H), 2.51 (br, 1H), 3.06 (dd, J = 6.0 Hz, J = 9.0 Hz, 1H), 3.13 (dd, J = 6.0 Hz, J = 9.0 Hz, 1H) 4.29 (m, 1H), 4.39 (m, 1H), 4.59 (m, 1H), 4.63 (m, 1H), 4.64 (m, 1H), 5.05 (m, 1H), 5.25 (d, J = 10.5 Hz, 1H), 5.32 (d, J = 17.0 Hz, 1H), 5.88 (m, 1H), 6.73 (m, 1H), 7.21–7.30 (m, 5H). Anal. calc. for C_21_H_30_N_2_O_6_ (406.21): C 62.05, H 7.44, N 6.89; found: C 62.01, H 7.46, N6.93 (see Additional file 1: Figure S29).

#### *Boc*-*L*-*Tyr*-*L*-*Thr*-*OAllyl* (**5i**)

White solids, yield 93 %. ^1^HNMR (500 MHz, CDCl_3_) δ = 1.17 (d, *J* = 6.0 Hz, 3H), 1.41 (s, 9H), 2.98 (m, 2H), 4.33 (m, 2H), 4.57 (m, 1H), 4.623 (m, 2H), 5.14 (m, 1H), 5.25 (d, *J* = 10.5 Hz, 1H), 5.31 (d, *J* = 17.0 Hz, 1H), 5.88 (m, 1H), 6.07 (br, 1H), 6.72 (d, *J* = 8.5 Hz, 2H), 7.04 (m, *J* = 8.5 Hz, 2H), Anal. calc. for C_21_H_30_N_2_O_7_ (422.21): C 59.70, H 7.16, N 6.63; found: C 59.72, H 7.19, N6.68 (see Additional file 1: Figure S32).

#### *Boc*-*L*-*β*-*Nap*-*L*-*Thr*-*OAllyl* (**5j**)

White solids, yield 90 %. ^1^HNMR (500 MHz, CDCl_3_) δ = 1.12 (d, J = 6.5 Hz, 3H), 1.43 (s, 9H), 3.19 (dd, *J*_1_ = 6.0 Hz, *J*_2_ = 7.5 Hz, 1H), 3.27 (dd, *J*_1_ = 6.0 Hz, *J*_2_ = 7.5 Hz, 1H), 4.27 (br, 1H), 4.50 (m, 2H), 4.61 (d, J = 6.0 Hz, 2H), 4.98 (m, 3H), 5.13 (d, *J* = 8.0 Hz, 1H), 5.83 (m, 1 H), 6.85 (m, 1 H), 7.42 (m, 1H), 7.45 (m, 2H), 7.64 (m, 1H), 7.74 (m, 3H); Anal. calc. for C_25_H_32_N_2_O_6_ (456.23): C65.77, H 7.07, N6.14; found: C65.72, H 7.04, N6.17 (see Additional file 1: Figure S36).

#### *Boc*-*D*-*Leu*-*L*-*Thr*-*OAllyl* (**5k**)

White solids, yield 92 %. ^1^H NMR (500 MHz, CDCl_3_): δ = 0.95 (d, *J* = 6.0 Hz, 3H), 0.96 (d, *J* = 4.5 Hz, 3H), 1.23 (d, *J* = 6.5 Hz, 3H), 1.45 (s, 9H), 1.49 (m, 1H), 1.69 (m, 3H), 4.18 (br, 1H), 4.36 (br, 1H), 4.59 (m, 1H), 4.66 (m, 2H), 4.90 (m, 1H), 5.25 (d, *J* = 10.5 Hz, 1H), 5.34 (d, *J* = 17 Hz 1H), 5.90 (m, 1 H), 6.92 (m, 1 H). Anal. calc. for C_18_H_32_N_2_O_6_ (372.23): C 58.05, H 8.66, N 7.52; found: C58.03, H 8.69, N7.56 (see Additional file 1: Figure S40).

#### *Boc*-*D*-*β*-*Nap*-*L*-*Thr*-*OAllyl* (**5l**)

White solids, yield 93 %. ^1^HNMR (500 MHz, CDCl_3_) δ = 1.00 (d, J = 6.0 Hz, 3H), 1.38 (s, 9H), 3.24 (dd, *J*_1_ = 6.0 Hz, *J*_2_ = 7.0 Hz, 1H), 3.29 (dd, *J*_1_ = 6.0 Hz, *J*_2_ = 7.0 Hz, 1H), 4.22 (br, 1H), 4.54 (m, 2H), 4.62 (d, J = 5.5 Hz, 2H), 5.03 (m, 1H), 5.24 (d, *J* = 10.5 Hz, 1H), 5.15 (d, *J* = 17.0 Hz 1H), 5.87 (m, 1 H), 6.69 (m, 1 H), 7.37 (m, 1H), 7.45 (m, 2H), 7.67 (m, 1H), 7.79 (m, 3H); Anal. calc. for C_25_H_32_N_2_O_6_ (456.23): C65.77, H 7.07, N6.14; found: C65.75, H 7.09, N6.20 (see Additional file 1: Figure S43).

#### Synthesis of Boc-AA-Z-∆Abu-OAllyl

Boc-N-AA-L- Thr-OAllyl **5** (2.0 mmol) was dissolved in 2 mL dry acetonitrile, DMAP (0.2 mmol) was added to above solution, followed by di-*tert*-butyl dicarbonate (3.3 equiv.) under rapid stirring at room temperature 2 h. Then TMG (0.4 mL) was added dropwise to above solution, the reaction was stirred overnight. Evaporation at reduced pressure gave a residue that was partitioned between diethyl ether (100 mL) and KHSO_4_ (50 mL, 1 mol dm^−3^). The organic phase was thoroughly washed with KHSO_4_ (1 mol dm^−3^), NaHCO_3_ (1 mol dm^−3^) and saturated brine (3×50 mL, each), and dried with MgSO_4_. Removal of the solvent afforded **8c** as an oil. The crude product was purified by means of silica-gel chromatography (Petroleum ether/Ethyl acetate = 8:1) to give the product.

##### *Boc*-*L*-*Leu*-*Z*-*ΔAbu*-*OAllyl* (**6a**)

Yellow viscous liquid, yield 90 %. ^1^H NMR (500 MHz, CDCl_3_): δ = 0.96 (m, 6H), 1.45 (s, 9H), 1.54 (m, 1H), 1.76 (d, J = 7.0 Hz, 3H), 1.77 (m, 2H), 4.27 (br, 1H), 4.65 (d, J = 4.5 Hz, 2H), 5.05 (br, 1H), 5.24 (d, J = 5.5 Hz, 1H), 5.33 (d, J = 17.0 Hz, 1H), 5.92 (m, 1 H), 6.83 (m, 1 H), 7.66 (br, 1H). ^13^C NMR (500 MHz, CDCl_3_): δ 14.20 (1C), 14.54 (1C), 22.92 (1C), 24.73 (1C), 28.28 (3C), 40.99 (1C), 53.24 (1C), 60.42 (1C), 65.89 (1C), 118.50 (1C), 125.97 (1C), 131.86 (1C), 134.47 (1C), 155.82 (1C), 163.96 (1C), 171.02 (1C), 173.84 (1C); EI-MS *m/z* 377.3 (M^+^ + Na) (see Additional file 1: Figures S2–S4).

##### *Boc*-*L*-*Val*-*Z*-*ΔAbu*-*OAllyl* (**6b**)

White solids, m.p. 88–89 °C, yield 92 %. ^1^H NMR (500 MHz, CDCl_3_): δ = 0.98 (t, *J* = 7.0 Hz, 3H), 1.03 (d, *J* = 7.0 Hz, 3H), 1.44 (s, 9H), 1.77 (d, *J* = 7.0 Hz, 3H), 2.20 (br, 1H), 4.12 (br, 1H), 4.64 (d, *J* = 5.5 Hz, 2H), 5.22 (d, *J* = 10.5 Hz, 1H), 5.24 (br, 1H), 5.32 (d, *J* = 17.0 Hz, 1H), 5.91 (m, 1H), 6.837 (m, 1H), 7.730 (br, 1H). ^13^C NMR (500 MHz, CDCl_3_): δ 14.47 (1C), 17.84 (1C), 19.29 (1C), 28.27 (3C), 28.40 (1C), 30.88 (1C), 59.98 (1C), 65.87 (1C), 118.51 (1C), 126.15 (1C), 131.84 (1C), 134.67 (1C), 155.99 (1C), 163.95 (1C), 170.45 (1C), EI-MS *m/z* 363.2 (M^+^ + Na) (see Additional file 1: Figures S6–S8).

##### *Boc*-*L*-*ILeu*-*Z*-*ΔAbu*-*OAllyl* (**6c**)

White solids, m.p. 107–109 °C, yield 94 %. ^1^H NMR (500 MHz, CDCl_3_): δ = 0.93 (t, *J* = 7.0 Hz, 3H), 0.96 (d, *J* = 7.5 Hz, 3H), 1.26 (m, 2H), 1.45 (s, 9H), 1.46 (m, 1H), 1.77 (d, *J* = 7.0 Hz, 3H), 2.05 (br, 1H), 4.22 (br, 1H), 4.66 (d, *J* = 5.5 Hz, 2H), 5.019 (br, 1H), 5.24 (d, *J* = 10.5 Hz, 1H), 5.33 (d, *J* = 17.0 Hz, 1H), 5.92 (m, 1H), 6.86 (m, 1H), 7.41 (br, 1H). ^13^C NMR (500 MHz, CDCl_3_): δ11.71 (1C), 14.30 (1C), 14.72 (1C), 26.37 (1C), 28.28 (3C), 28.28 (1C), 37.07 (1C), 58.43 (1C), 65.96 (1C), 118.62 (1C), 125.82 (1C), 131.82 (1C), 134.69 (1C), 155.92 (1C), 164.00 (1C), 170.39 (1C). EI-MS *m/z* 377.4 (M^+^ + Na) (see Additional file 1: Figures S10–S12).

##### *Boc*-*L*-*Trp*-*Z*-*ΔAbu*-*OAllyl* (**6d**)

White solids, m.p. 126–127 °C, yield 88 %. ^1^H NMR (500 MHz, CDCl_3_): δ = 1.42 (s, 9H), 1.67 (t, *J* = 7.0 Hz, 3H), 3.30 (m, 2H), 4.61 (m, 3H), 5.13 (m, 1H), 5.23 (d, *J* = 10.5 Hz, 1H), 5.31 (d, *J* = 17.0 Hz, 1H), 5.90 (m, 1H), 6.80 (m, 1H), 7.14 (m, 2H), 7.21 (m, 1H), 7.36 (d, *J* = 8.0 Hz, 1H), 7.67 (d, *J* = 8.0 Hz, 1H); 8.10 (br, 1H). ^13^C NMR (500 MHz, CDCl_3_): δ14.54 (1C), 28.22 (3C), 29.68 (1C), 55.27 (1C), 61.61 (1C), 65.81 (1C), 111.14 (1C), 118.41 (1C), 118.83 (1C), 119.78 (1C), 122.27 (1C), 123.32 (1C), 125.73 (1C), 127.49 (1C), 128.5 (1C), 131.83 (1C), 134.45 (1C), 136.17 (1C), 163.77 (1C), 170.09 (1C), 170.09 (1C). EI-MS *m/z* 450.3 (M^+^ + Na) (see Additional file 1: Figures S14–S16).

##### *Boc*-*L*- *(p*-*Br)* -*Phe*-*Z*-*ΔAbu*-*OAllyl* (**6e**)

Yellow viscous liquid, yield 88 %. ^1^H NMR (500 MHz, CDCl_3_): δ = 1.11 (d, J = 7.0 Hz, 3H), 1.59 (s, 9H), 3.27 (dd, J = 2.5 Hz, J = 12.0 Hz, 1H), 3.56 (dd, J = 2.5 Hz, J = 12.0 Hz, 1H), 4.64 (m, 2H), 4.817 (m, 1H), 5.23 (d, J = 10.5 Hz, 1H), 5.28 (d, J = 17.0 Hz, 1H), 5.87 (m, 1H), 7.04 (d, J = 8.0 Hz, 2H), 7.19 (m, 1H), 7.40 (d, J = 8.0 Hz, 2H). ^13^C NMR (500 MHz, CDCl_3_): δ13.35 (1C), 27.85 (1C), 28.09 (3C), 34.15 (1C), 60.45 (1C), 66.29 (1C), 118.71 (1C), 121.89 (1C), 131.47 (1C), 131.63 (2C), 131.99 (2C), 132.49 (1C), 144.39 (1C), 148.67 (1C), 149.82 (1C), 161.39 (1C), 168.68 (1C). EI-MS *m/z* 491.2 (M^+^ + H + Na) (see Additional file 1: Figures S18–S20).

##### *Boc*-*L*-*Pro*-*Z*-*ΔAbu*-*OAllyl* (**6f**)

Yellow viscous liquid, yield 90 %. ^1^H NMR (500 MHz, CDCl_3_): δ = 1.48 (s, 9H), 1.78 (d, J = 6.5 Hz 3H), 1.94 (m, 3H), 2.20 (m, 1H), 3.42 (m, 2H), 4.38 (m, 1H), 4.65 (m, 2H), 5.24 (d, J = 10.5 Hz 1H), 5.33 (d, J = 17.0 Hz 1H), 5.92 (m, 1H), 6.82 (m, 1H). ^13^C NMR (500 MHz, CDCl_3_): δ 14.75 (1C), 24.61 (1C), 28.35 (3C), 31.36 (1C), 47.16 (1C), 60.04 (1C), 61.49 (1C), 65.79 (1C), 118.69 (1C), 126.63 (1C), 131.97 (1C), 133.57 (1C), 155.89 (1C), 163.94 (1C), 171.17 (1C). EI-MS *m/z* 361.3 (M^+^ + Na) (see Additional file 1: Figures S22–S24).

##### *Boc*-*L*-*His*-*Z*-*ΔAbu*-*OAllyl* (**6g**)

Yellow viscous liquid, yield 88 %. ^1^H NMR (500 MHz, CDCl_3_): δ = 1.58 (s, 18H), 1.73 (d, J = 7.5 Hz, 3H), 3.34 (dd, J = 6.0 Hz, J = 8.5 Hz, 1H), 3.56 (dd, J = 6.0 Hz, J = 8.5 Hz, 1H), 4.66 (m, 2H), 4.75 (m, 1H), 5.23 (d, J = 10.5 Hz 1H), 5.311 (d, J = 17.0 Hz 1H), 5.90 (m, 1H), 7.14 (s, 1H), 7.33 (m, 1H), 7.90 (s, 1H). ^13^CNMR (500 MHz, CDCl_3_): δ12.82 (1C), 27.00 (3C), 26.83 (3C), 26.69 (1C), 28.67 (1C), 58.18 (1C), 65.19 (1C), 114.39 (1C), 117.55 (1C), 121.45 (1C), 130.55 (1C), 135.49 (1C), 136.04 (1C), 143.58 (1C), 147.46 (1C), 149.45 (1C), 160.83 (1C), 168.29 (1C). EI-MS *m/z* 479.3 (M^+^ + H) (see Additional file 1: Figures S26–S28).

##### *Boc*-*L*-*Phe*-*Z*-*ΔAbu*-*OAllyl* (**6h**)

White solids, m.p. 108–109 °C, yield 91 %. ^1^H NMR (500 MHz, CDCl_3_): δ = 1.41 (s, 9H), 1.70 (d, J = 7.0 Hz, 3H), 3.06 (dd, J = 6.5 Hz, J = 7.5 Hz, 1H), 3.09 (dd, J = 6.5 Hz, J = 7.5 Hz, 1H), 4.49 (m, 1H), 4.64 (m, 2H), 5.00 (m, 1H), 5.246 (d, J = 11.5 Hz 1H), 5.312 (d, J = 17.0 Hz, 1H), 5.90 (m, 1H), 6.83 (q, J = 7.0 Hz, 1H), 7.25 (m, 5H). ^13^C NMR (500 MHz, CDCl_3_): δ = 14.55 (1C), 28.24 (3C), 38.63 (1C), 52.31 (1C), 55.90 (1C), 70.26 (1C), 125.75 (1C), 126.99 (1C), 128.69 (3C), 129.40 (3C), 134.53 (1C), 136.45 (1C), 155.55 (1C), 164.63 (1C), 169.72 (1C). EI-MS *m/z* 411.3 (M^+^ + Na) (see Additional file 1: Figures S30, S31)

##### *Boc*-*L*-*Tyr*-*Z*-*ΔAbu*-*OAllyl* (**6i**)

White solids, m.p. 87–89 °C, yield 92 %. ^1^H NMR (500 MHz, CDCl_3_): δ = 1.41 (s, 9H), 1.69 (d, J = 7.0 Hz, 3H), 3.03 (dd, J_1_ = 6.0 Hz, J_2_ = 8.0 Hz, 1H), 3.17 (dd, J_1_ = 6.0 Hz, J_2_ = 8.0 Hz, 1H), 4.48 (m, 1H), 4.63 (d, J = 6.0 Hz, 2H), 5.07 (m, 1H), 5.23 (d, J = 10.5 Hz, 1H), 5.32 (d, J = 17.0 Hz, 1H), 5.90 (m, 1H), 6.82 (m, 1H), 7.10 (d, *J* = 8.0 Hz, 2H), 7.5 (d, *J* = 8.0 Hz, 2H). 7.48 (br, 1H), ^13^C NMR (500 MHz, CDCl_3_): δ14.59 (1C), 27.70 (3C), 28.25 (1C), 37.14 (1C), 55.76 (1C), 65.93 (1C), 118.55 (1C), 121.48 (2C), 125.77 (1C), 130.37 (2C), 131.85 (1C), 134.74 (1C), 150.09 (1C), 151.84 (1C), 155.53 (1C), 163.81 (1C), 169.60 (1C). EI-MS *m/z* 405.2 (M^+^ + 1) (see Additional file 1: Figures S33–S35).

##### *Boc*-*L*-*β*-*Nap*-*Z*-*Δabu*–*OAllyl* (**6j**)

White solids, m.p. 108–109 °C, yield 93 %. ^1^H NMR (500 MHz, CDCl_3_): δ = 0.64 (d, *J* = 7.0 Hz, 3H), 1.67 (s, 9H), 3.50 (dd *J* = 2.5 Hz, *J* = 11.5 Hz, 1H), 3.77 (d, *J* = 5.0 Hz, *J* = 9.0 Hz, 1H), 4.63 (m, 2H), 4.91 (m, 1H), 5.22 (d, *J* = 11.5 Hz, 1H), 5.30 (d, *J* = 17.0 Hz, 1H), 5.87 (m, 1H), 7.06 (q, *J* = 7.5 Hz, 1H), 7.20 (m, 1H), 7.44 (m, 2H), 7.70 (s, 1H), 7.74 ( (m, 3H).^13^C NMR (500 MHz, CDCl_3_): δ12.97 (1C), 27.73 (1C), 28.15 (3C), 34.87 (1C), 60.92 (1C), 66.22 (1C), 118.65 (1C), 122.11 (1C), 126.11 (1C), 126.38 (1C), 127.56 (1C), 128.55 (1C), 129.16 (1C), 130.98 (1C), 131.50 (1C), 132.67 (1C), 133.40 (1C), 144.45 (1C), 148.77 (1C), 149.88 (1C), 161.45 (1C), 168.99 (1C), 171.17 (1C). EI-MS *m/z* 461.3 (M^+^ + Na) (see Additional file 1: Figures S37–S39).

##### *Boc*-*D*-*Leu*-*Z*-*ΔAbu*-*OAllyl* (**6k**)

Yellow viscous liquid, yield 89 %. ^1^H NMR (500 MHz, CDCl_3_): δ = 0.96 (t, *J* = 6.5 Hz, 6H), 1.46 (s, 9H), 1.56 (m, 2H), 1.66 (m, 2H), 1.75 (d, *J* = 7.0 Hz, 3H), 4.22 (br, 1H), 4.65 (d, *J* = 6.0 Hz, 2H), 4.90 (br, 1H), 5.26 (d, *J* = 10.5 Hz, 1H), 5.35 (d *J* = 17.0 Hz, 1H), 5.92 (m, 1H), 6.85 (m, 1H), 7.48 (br, 1H). ^13^C NMR (500 MHz, CDCl_3_): δ14.21 (1C), 14.53 (1C), 22.92 (1C), 24.73 (1C), 28.29 (3C), 41.00 (1C), 53.24 (1C), 60.44 (1C), 65.89 (1C), 118.50 (1C), 125.97 (1C), 131.83 (1C), 134.47 (1C), 155.84 (1C), 163.99 (1C), 171.02 (1C), 173.88 (1C); EI-MS *m/z* 355.2 (M^+^ + H) (see Additional file 1: Figures S41, S42).

##### *Boc*-*D*-*β*-*Nap*-*Z*-*Δabu*–*Oallyl* (**6l**)

White solids, m.p. 109–110 °C, yield 92 %. ^1^H NMR (500 MHz, CDCl_3_): δ = 0.65 (d, *J* = 7.5 Hz, 3H), 1.61 (s, 9H), 3.49 (dd, *J* = 2.5 Hz, *J* = 11.5 Hz, 1H), 3.770 (d, *J* = 5.0 Hz, *J* = 9.0 Hz, 1H), 4.62 (m, 2H), 4.90 (m, 1H), 5.22 (d, *J* = 10.5 Hz, 1H), 5.26 (d, *J* = 17.0 Hz, 1H), 5.86 (m, 1H), 7.05 (q, *J* = 7.5 Hz, 1H), 7.21 (m, 1H), 7.44 (m, 2H), 7.71 (s, 1H), 7.74 (m, 3H). ^13^C NMR (500 MHz, CDCl_3_): δ12.97 (1C), 21.07 (1H), 28.14 (3C), 34.87 (1C), 60.92 (1C), 66.21 (1C), 118.63 (1C), 122.10 (1C), 126.11 (1C), 126.38 (1C), 127.63 (1C), 128.54 (1C), 129.15 (1C), 130.96 (1C), 131.50 (1C), 132.68 (1C), 133.40 (1C), 144.43 (1C), 148.77 (1C), 149.87 (1C), 161.44 (1C), 168.98 (1C), 171.18 (1C). EI-MS *m/z* 461.2 (M^+^ + Na) (see Additional file 1: Figures S44, S45).

## Conclusions

α,β-Dehydroamino acids play an important role in the design and synthesis of biological peptide and the study of its structure-activity relationship, while The synthesis of peptides containing dehydroamino acids were a challenge. A variety of dehydroamino acid derivatives were synthesized. The results showed that this methods could be carried out as a one-pot procedure, and had high stereoselectivity. 2D NMR NOESY and X-ray crystal diffraction determined Z configuration of double bond.

## Supplementary information

All additional information pertaining to characterization of the complexes using ^1^H NMR, ^13^C NMR and EI-MS spectra are given in the supporting information available at XXX.

## Additional file

10.1186/s40064-016-2005-z ^1^H NMR, ^13^C NMR and MS spectrums of some intermediates and all dehydrodipeptides.
