# Supplementary material for: Stereoselectively synthesis and structural confirmation of dehydrodipeptides with dehydrobutyrine
Source: Springerplus. 2016 Apr 1;5:400. doi: 10.1186/s40064-016-2005-z (PMC4816936; doi:10.1186/s40064-016-2005-z)
Supplement: Supplementary file 1 — 10.1186/s40064-016-2005-z 1H NMR, 13C NMR and MS spectrums of some intermediates and all dehydrodipeptides. [file 40064_2016_2005_MOESM1_ESM.doc]

Surporting Information(SI)

**Stereoselectively Synthesis and Structural Confirmation of Dehydrodipeptides with Dehydrobutyrine**

**Xia Tian, Linna Li, Jianrong Han, Xiaoli Zhen And Shouxin Liu** ^a^College of Sciences, Hebei University of Science & Technology, Shijiazhuang 050018,People’s Republic of China

^b^State Key Laboratory Breeding Base-Hebei Province Key Laboratory of Molecular Chemistry for Drug, Hebei University of Science &Technology, Shijiazhuang 050018, People’s Republic of China

*Boc-L-Leu-L-Thr-OAllyl (5a)*

*
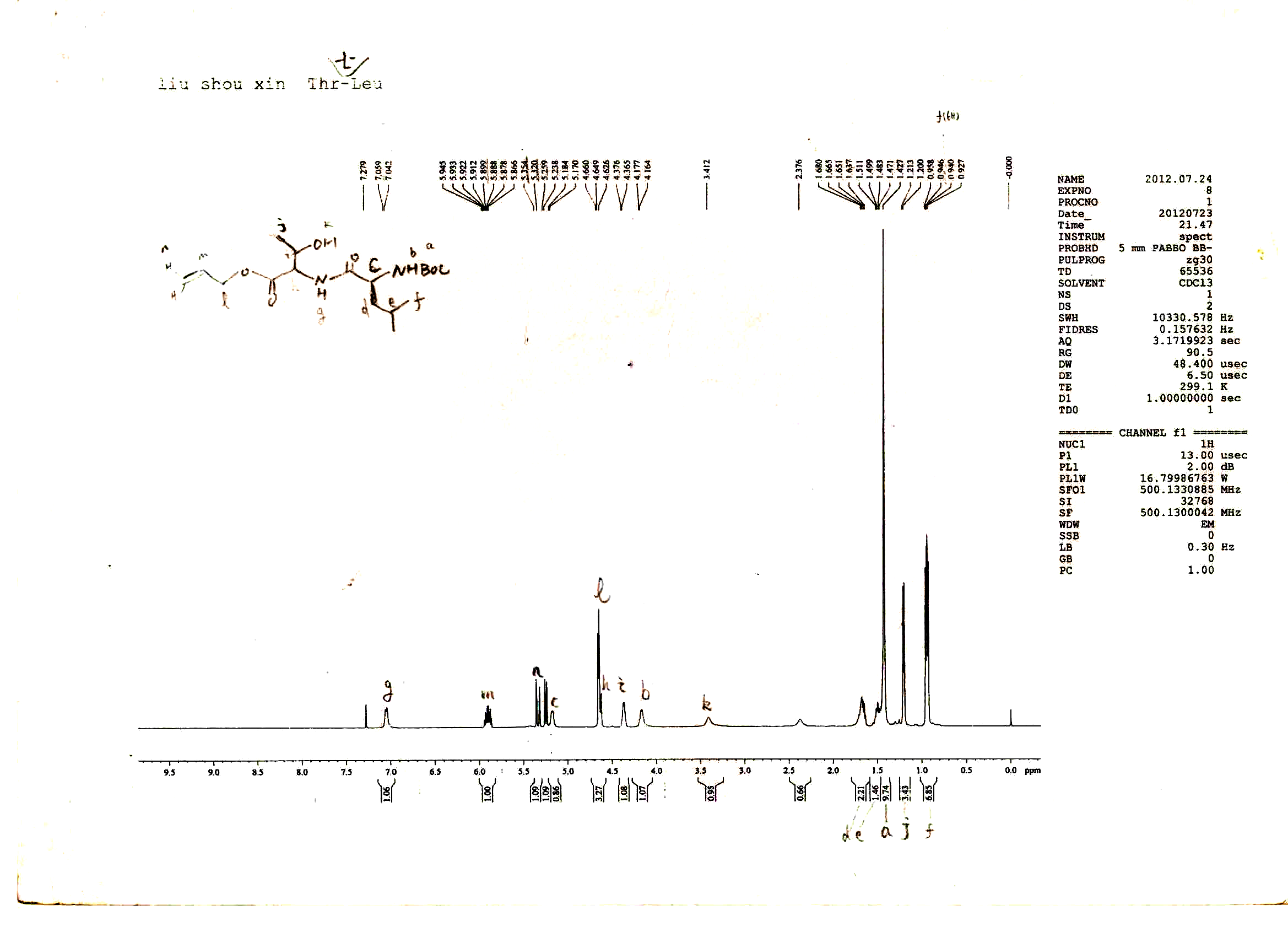
*

Figure S1 .^1^H NMR spectrum of *Boc-L-Leu-L-Thr-OAllyl*

*Boc-L-Leu-Z-ΔAbu-OAllyl (6a)*

*
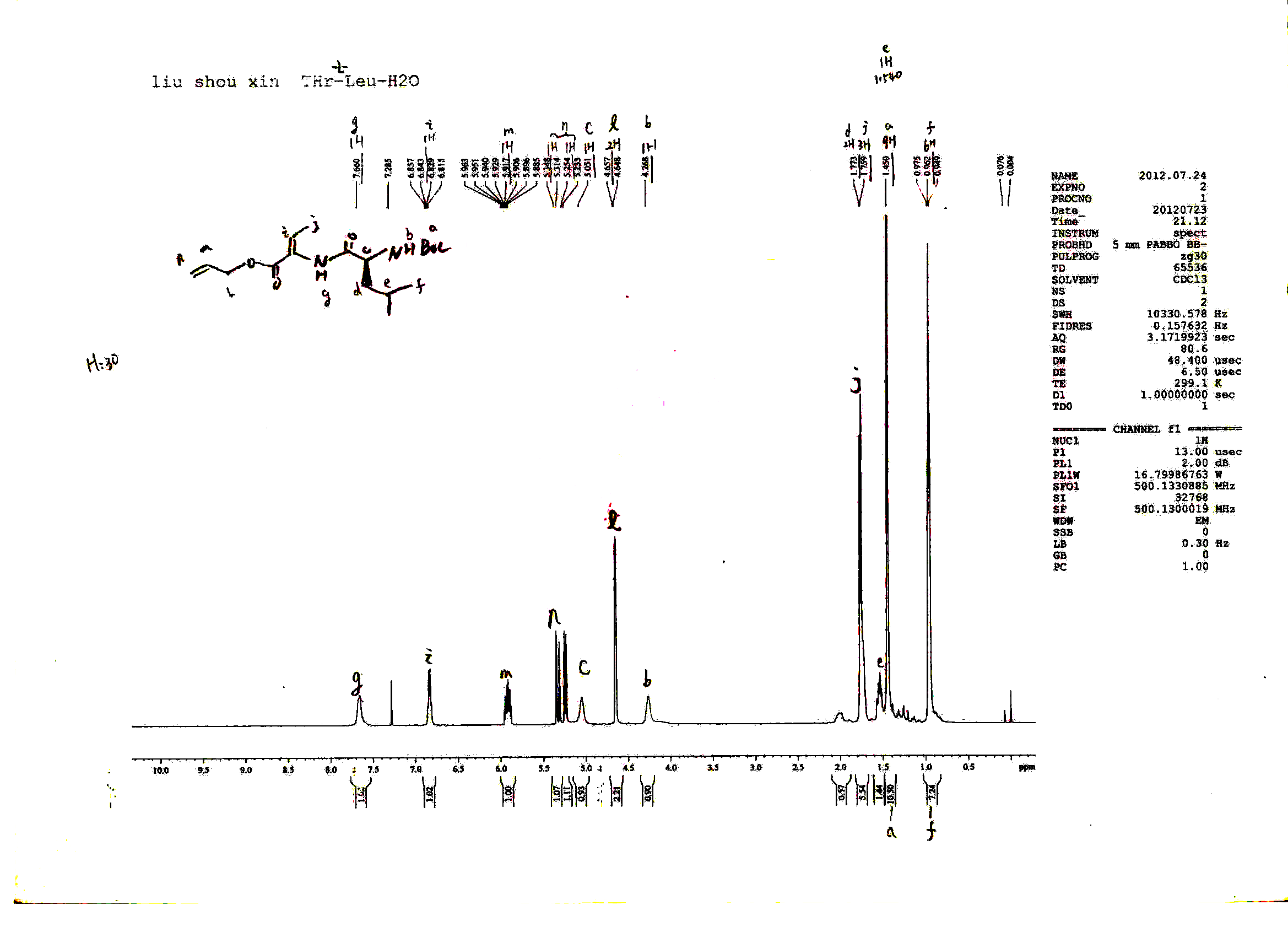
*

Figure S2 .^1^H NMR spectrum of *Boc-L-Leu-Z-ΔAbu-OAllyl*


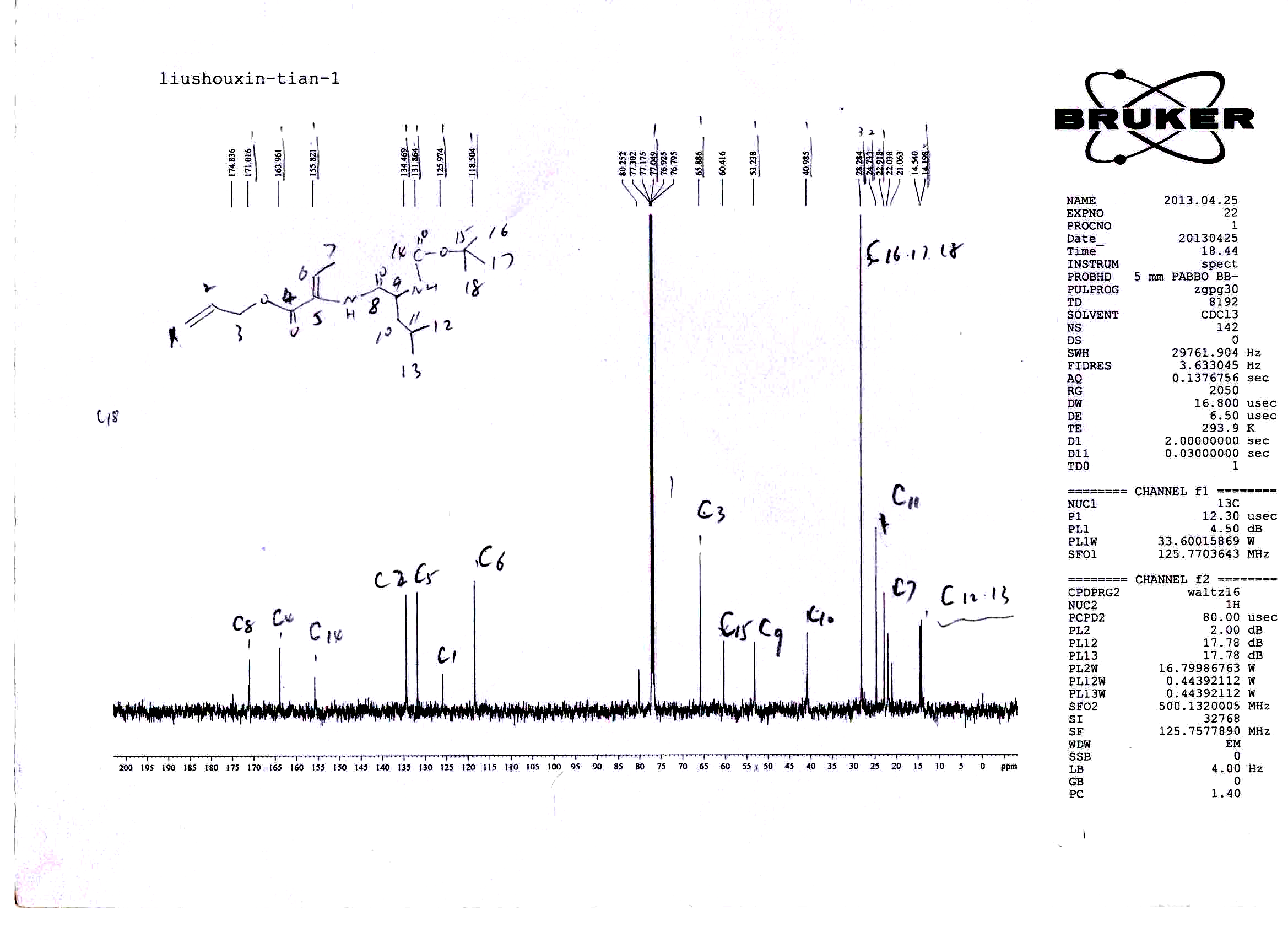


Figure S3 . CNMR spectrum of *Boc-L-Leu-Z-ΔAbu-OAllyl*

Figure S4 . MS spectrum of *Boc-L-Leu-Z-ΔAbu-OAllyl*

*Boc-L-Val-L-Thr-OAllyl (5b)*

**
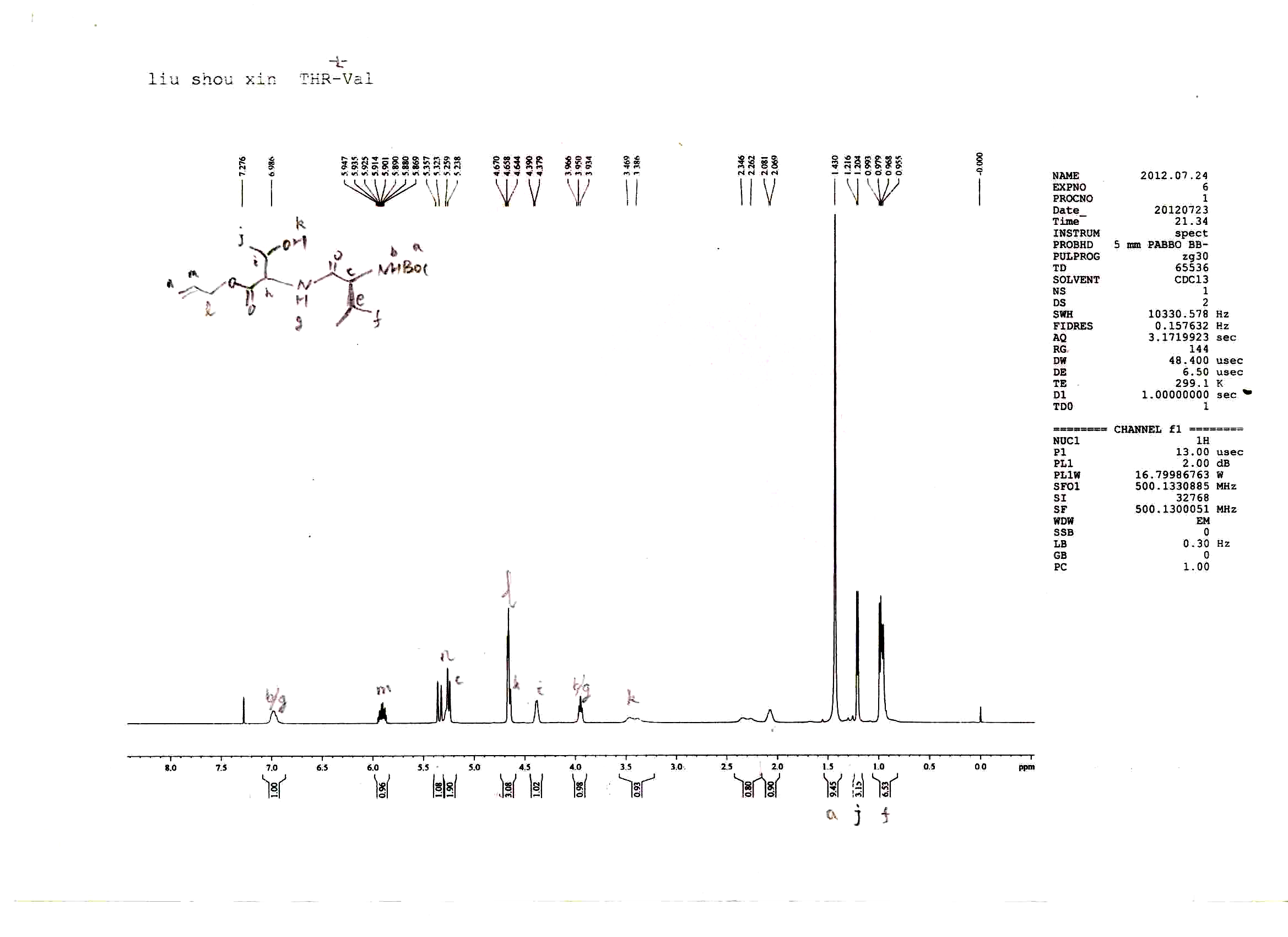
**

Figure S5 .^1^H NMR spectrum of *Boc-L-Val-L-Thr-OAllyl*

*Boc-L-Val-Z-ΔAbu-OAllyl (6b)*

**
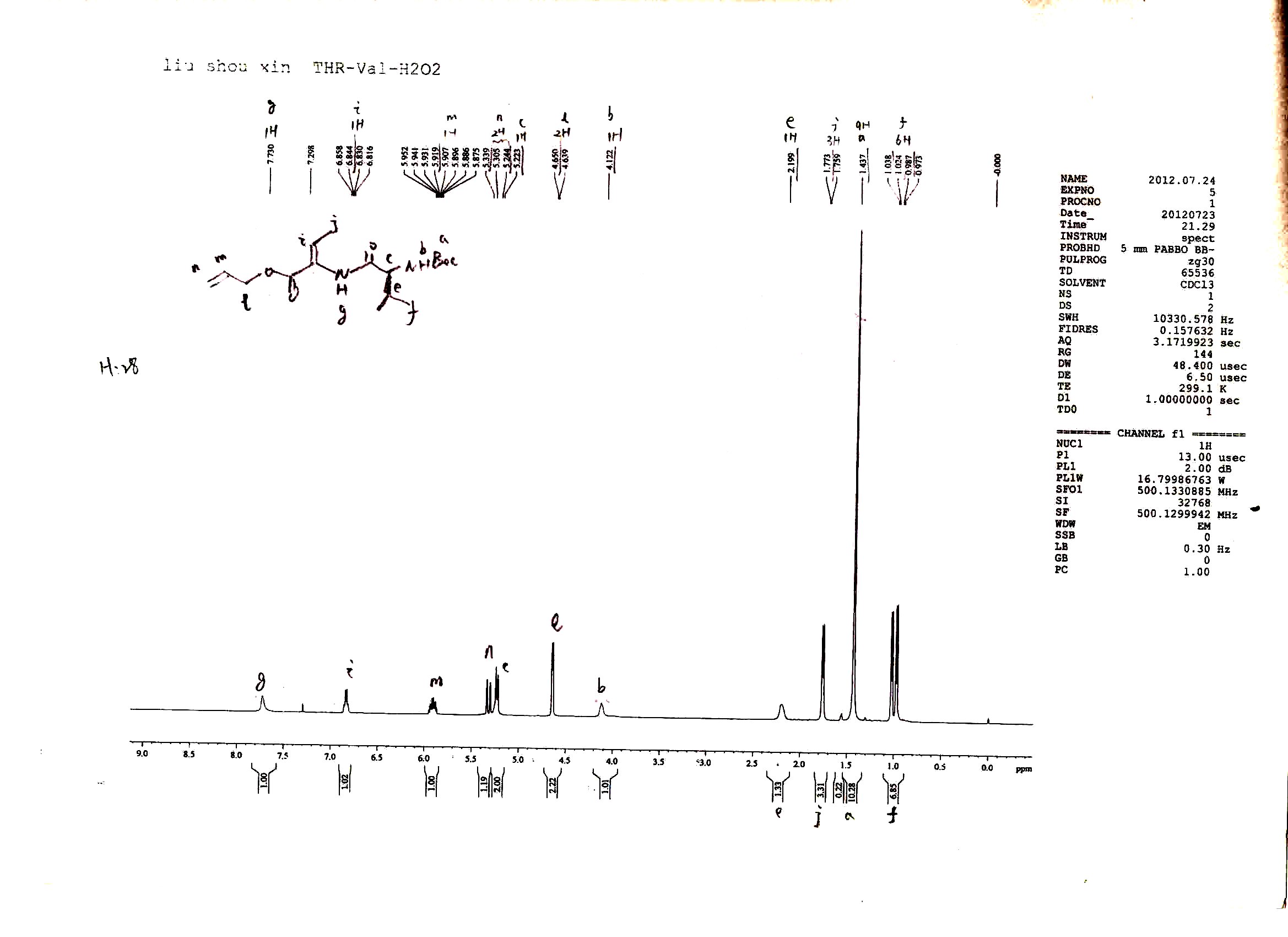
**

Figure S6 .^1^H NMR spectrum of *Boc-L-Val-Z-ΔAbu-OAllyl*

**
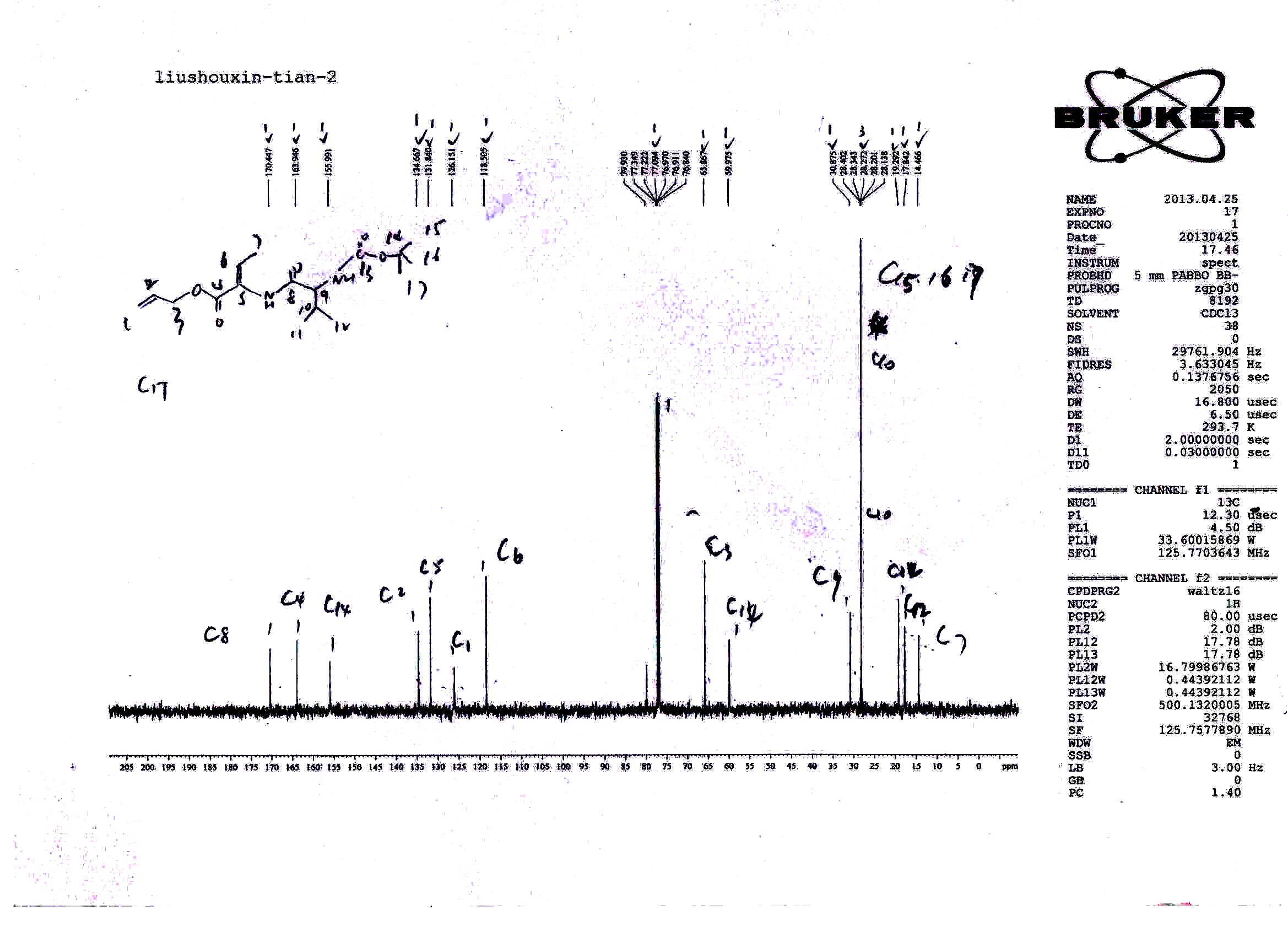
**

Figure S7 . CNMR spectrum of *Boc-L-Val-Z-ΔAbu-OAllyl*

Figure S8 . MS spectrum of *Boc-L-Val-Z-ΔAbu-OAllyl*

*Boc-L-ILeu-L-Thr-OAllyl(5c)
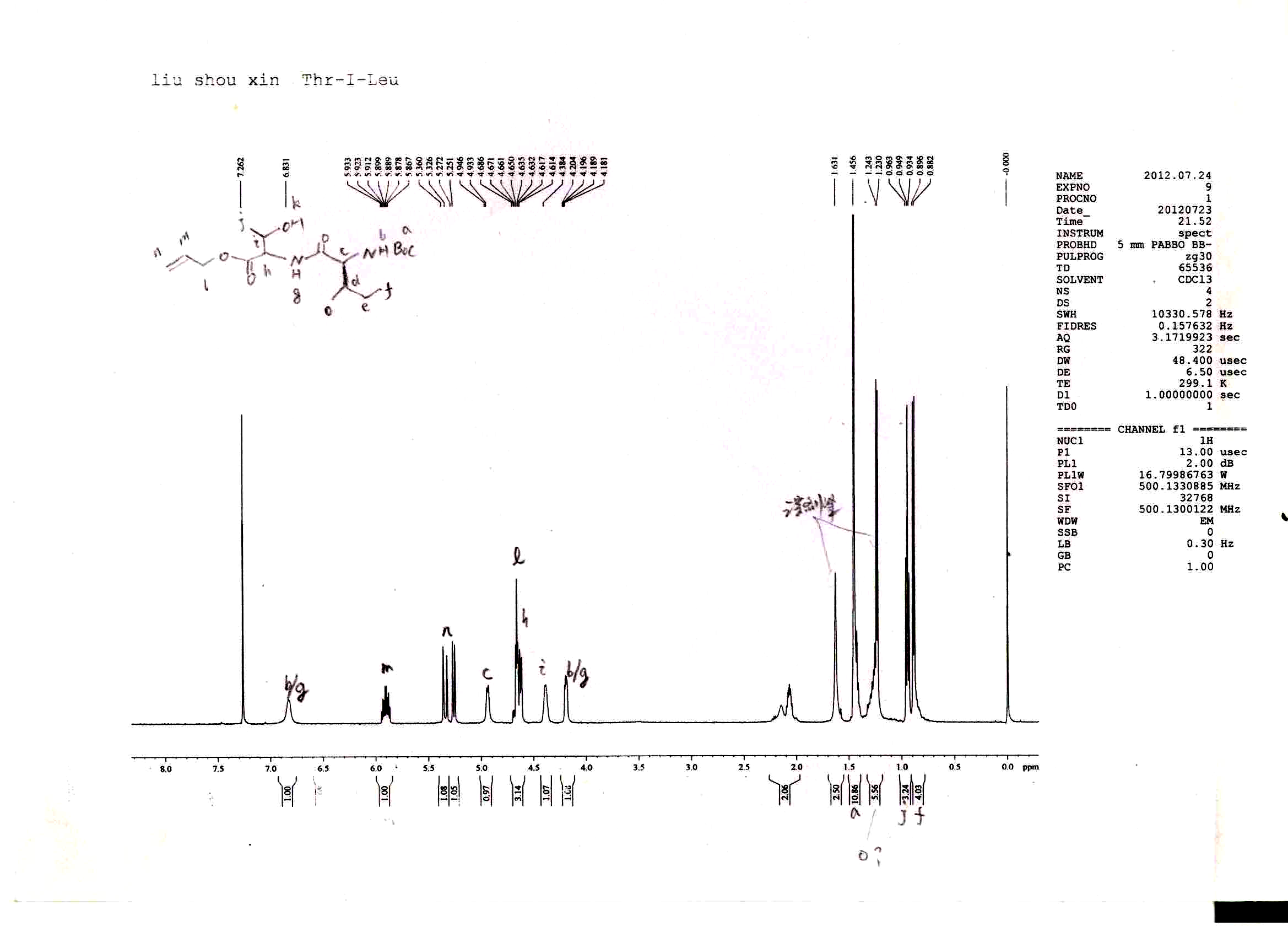
*

Figure S9 .^1^H NMR spectrum of *Boc-L-ILeu-L-Thr-OAllyl (5c)*

*Boc-L-IL-Z-ΔAbu-OAllyl (6c)*

*
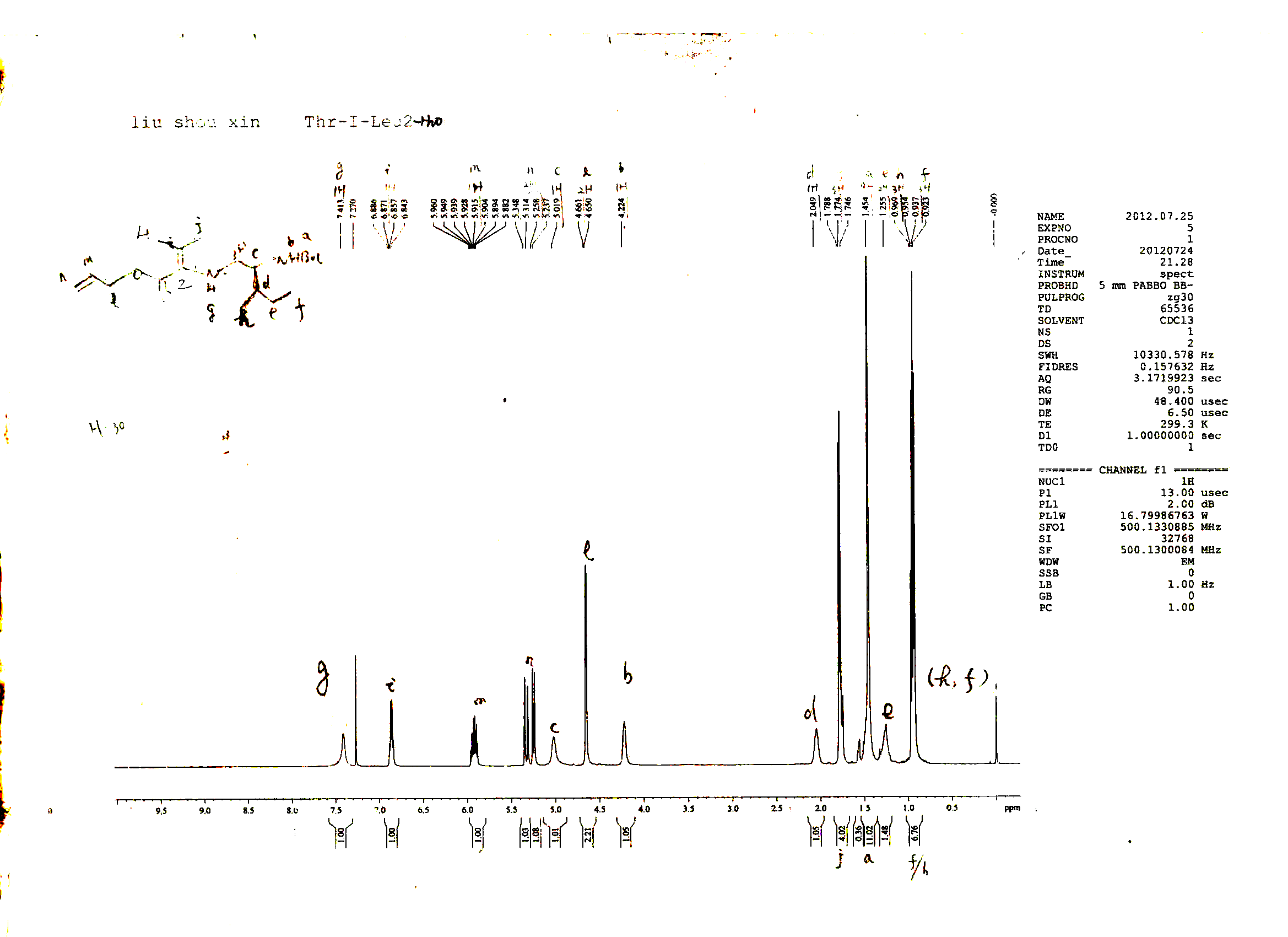
*

Figure S10 .^1^H NMR spectrum of *Boc-L-IL-Z-ΔAbu-OAllyl (6c)*


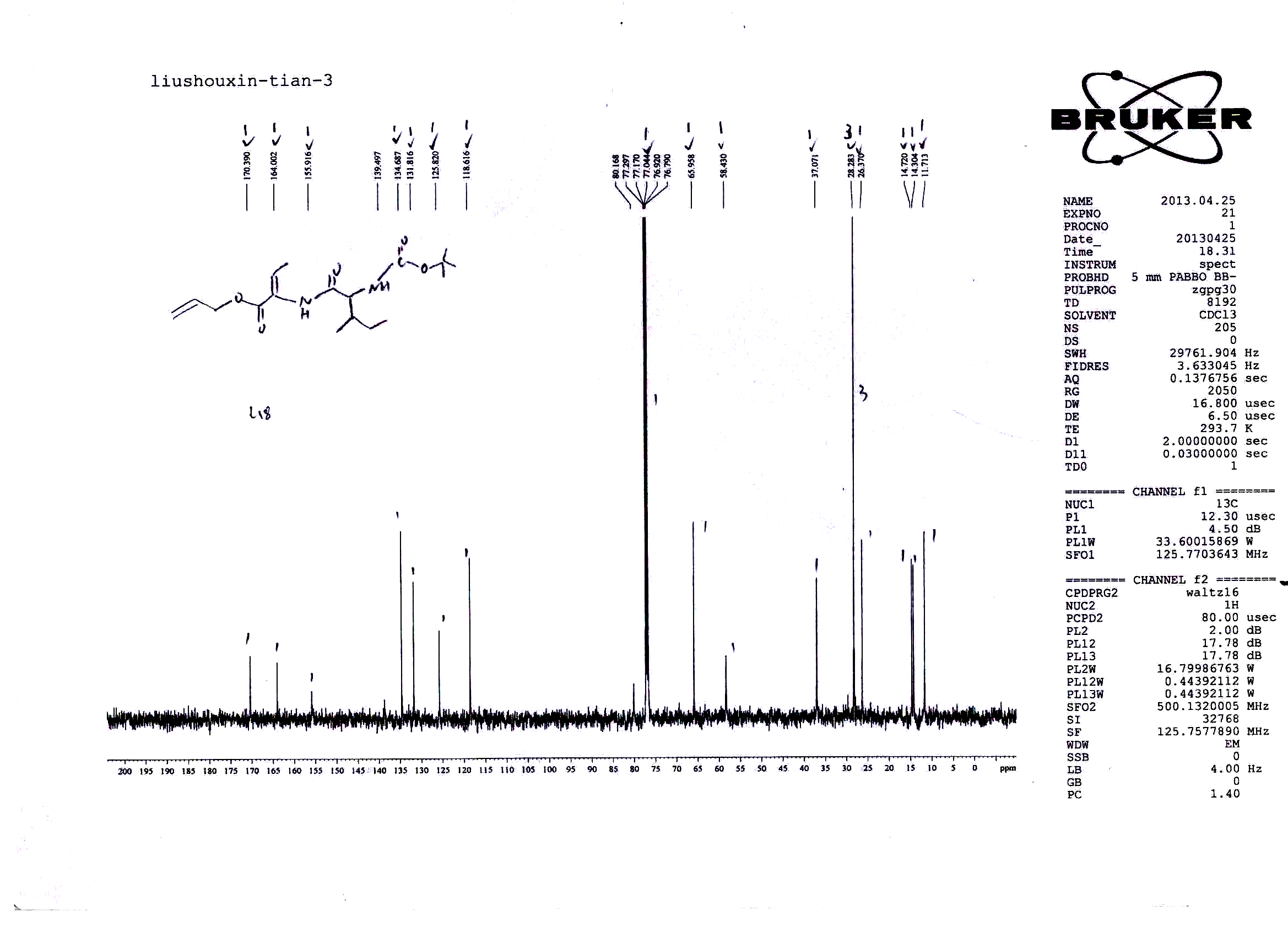


Figure S11 .CNMR spectrum of *Boc-L-IL-Z-ΔAbu-OAllyl (6c)*


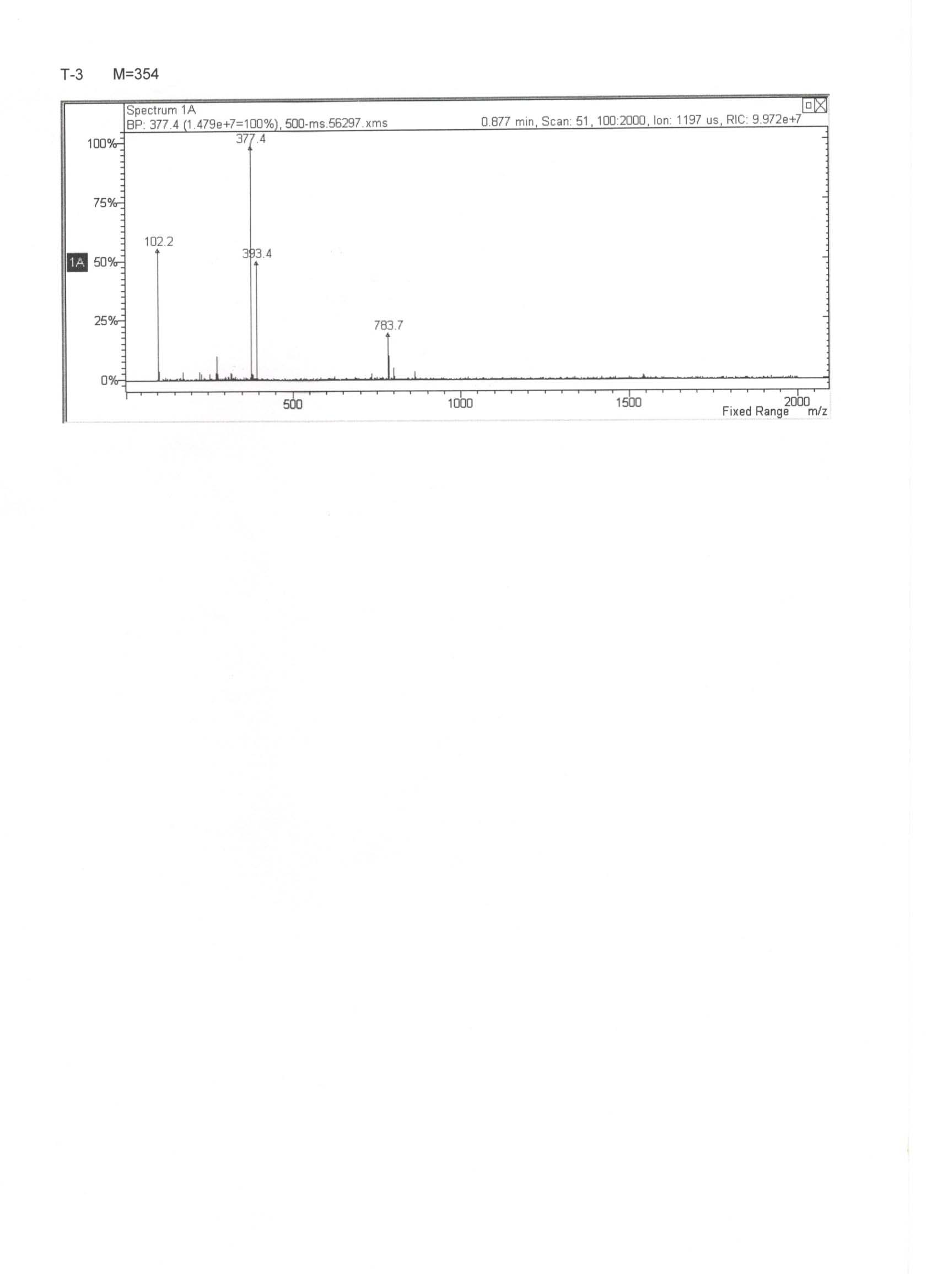


Figure S12 .MS spectrum of *Boc-L-IL-Z-ΔAbu-OAllyl (6c)*

*Boc-L-Trp-L-Thr-OAllyl (5d)*


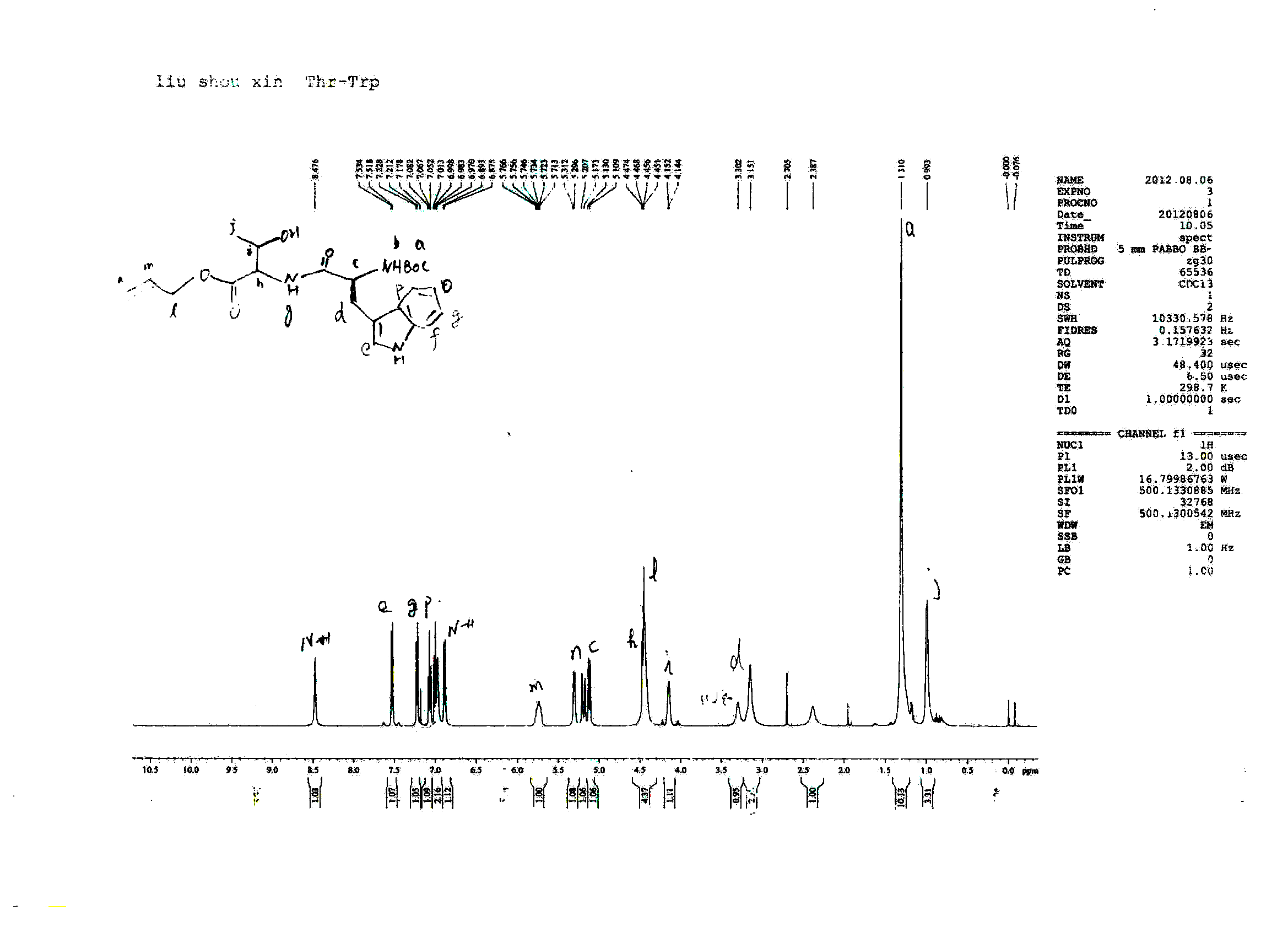


Figure S13 .^1^H NMR spectrum of *Boc-L-Trp-L-Thr-OAllyl (5d)*

*Boc-L-Trp-Z-ΔAbu-OAllyl (6d)*


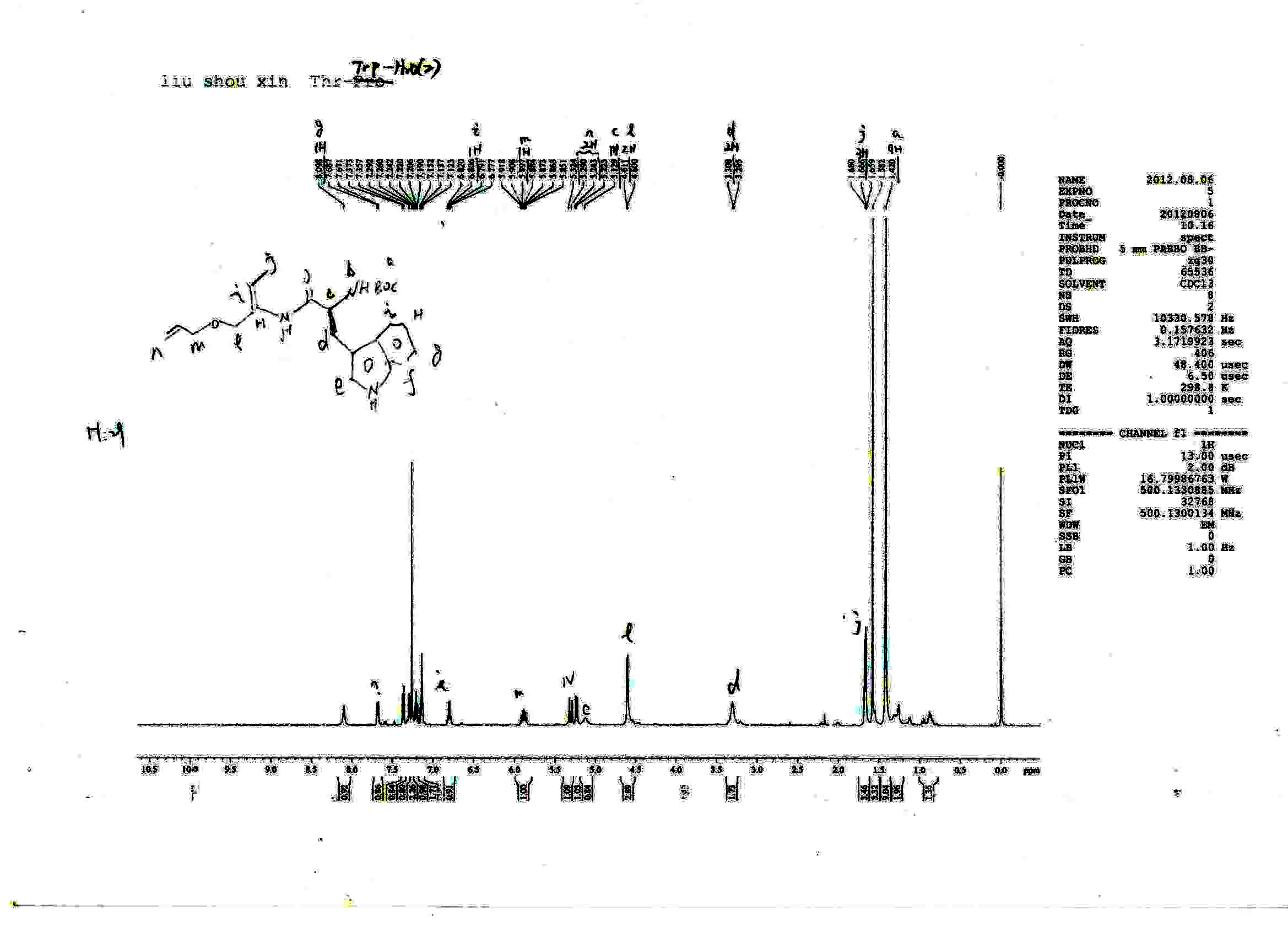


Figure S14 .^1^H NMR spectrum of *Boc-L-Trp-Z-ΔAbu-OAllyl (6d)*

*
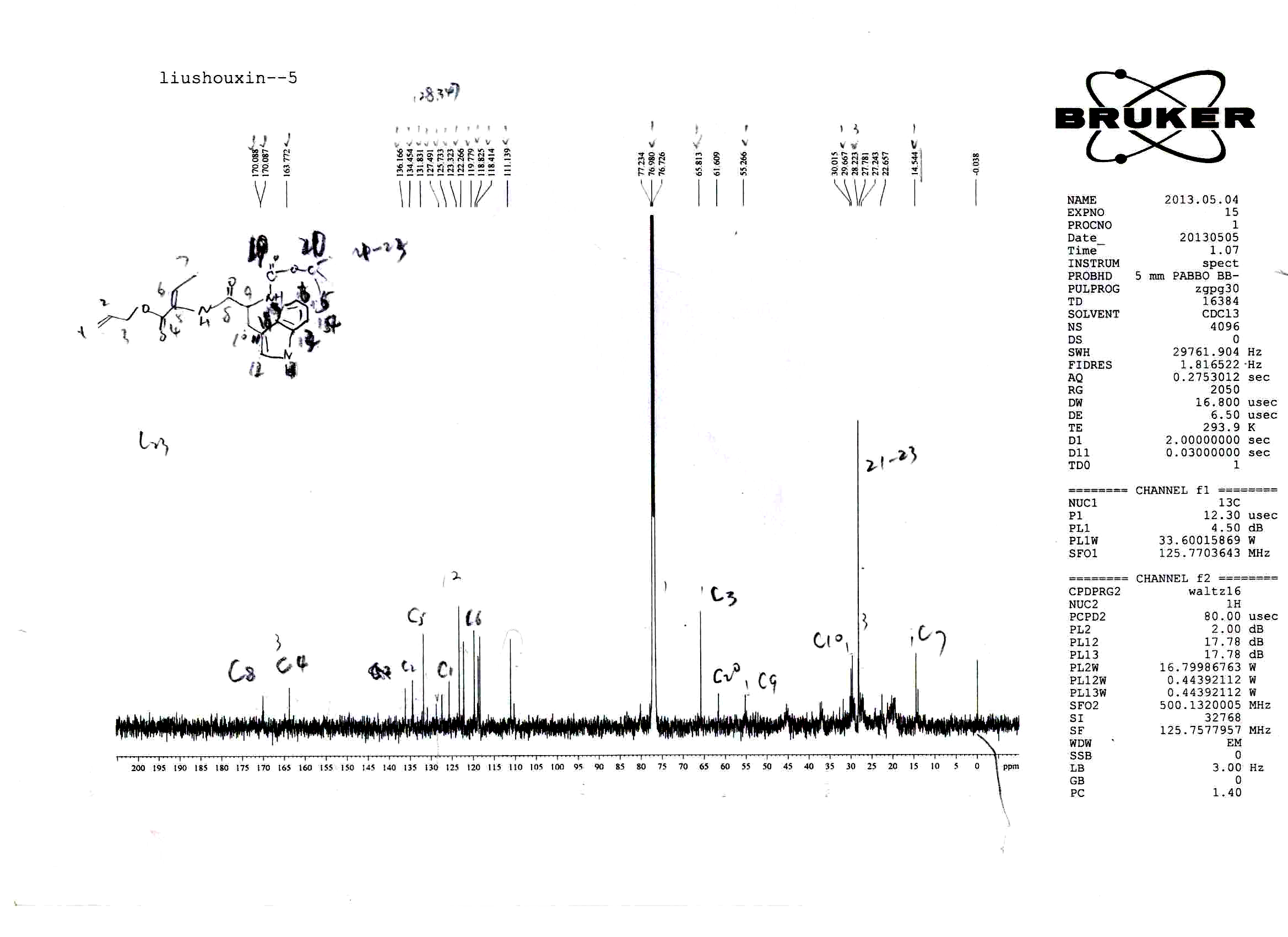
*

Figure S15 .CNMR spectrum of *Boc-L-Trp-Z-ΔAbu-OAllyl (6d)*

*
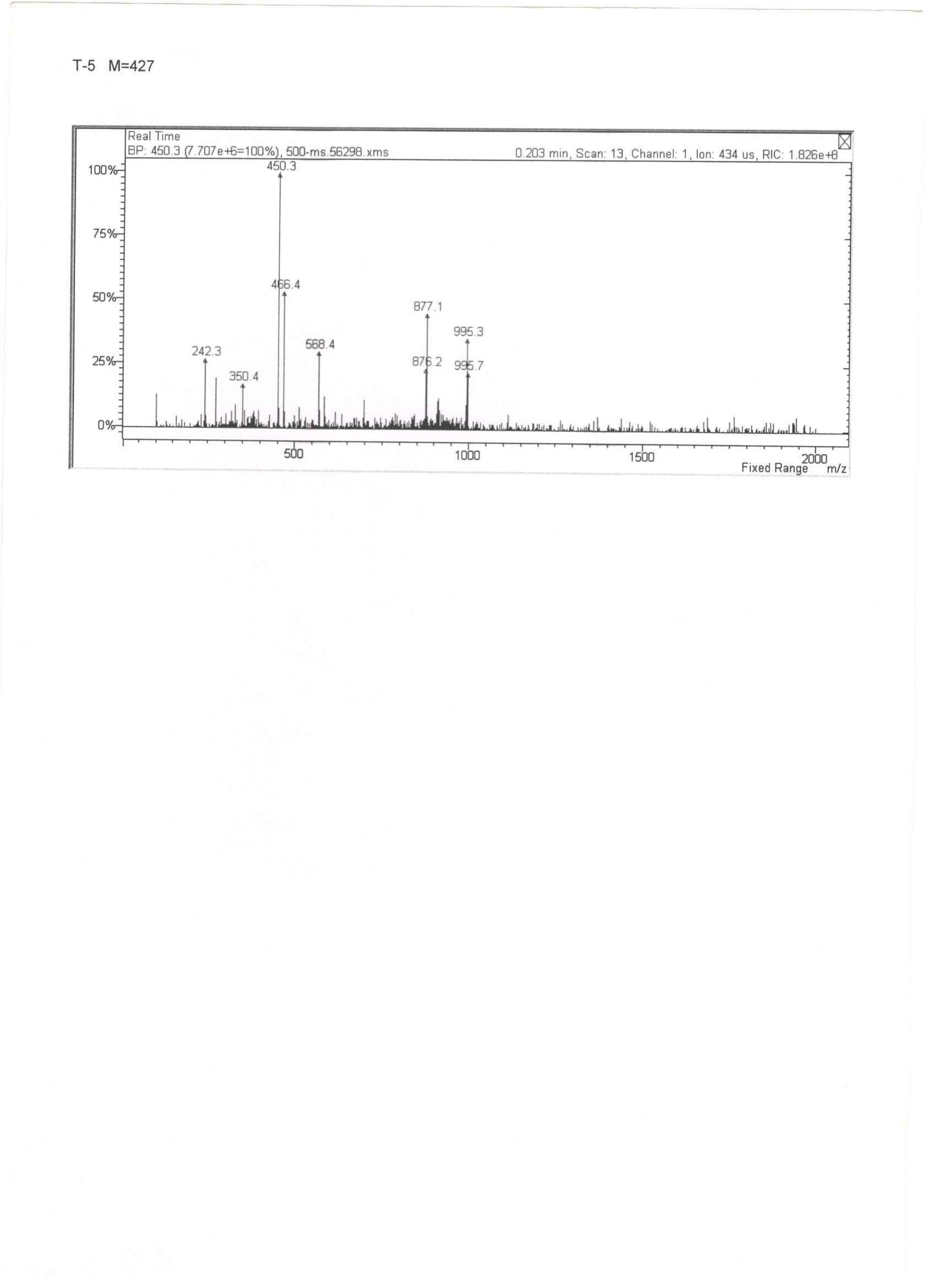
*

Figure S16 .MS spectrum of *Boc-L-Trp-Z-ΔAbu-OAllyl (6d)*

*Boc-L-(p-Br)-Phe-L-Thr-OAllyl (5e)*

*
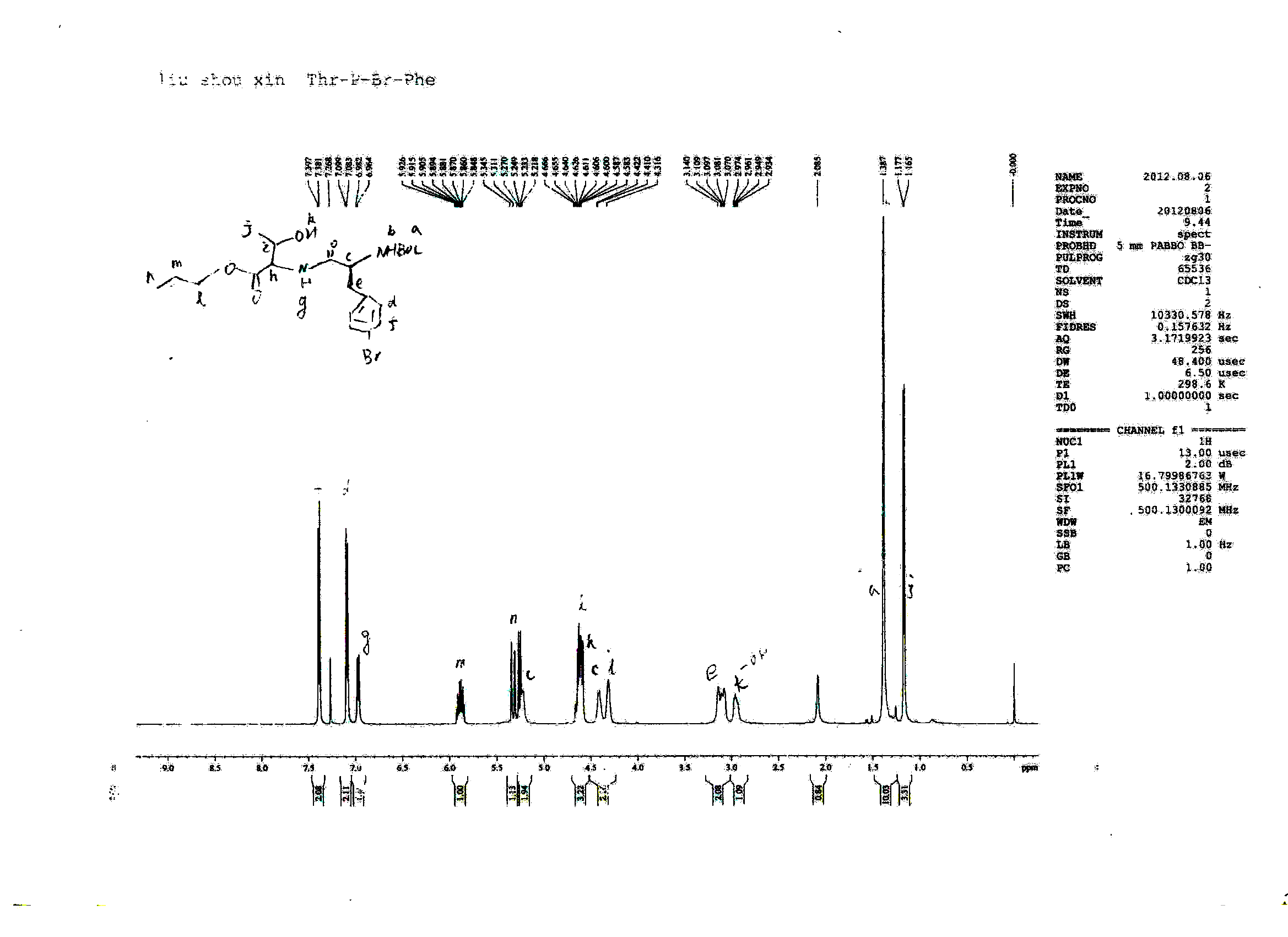
*

Figure S17 .^1^H NMR spectrum of *Boc-L-(p-Br)-Phe-L-Thr-OAllyl (5e)*

*Boc- p-Br L-Phe-Z-ΔAbu-OAllyl (6e)*

*
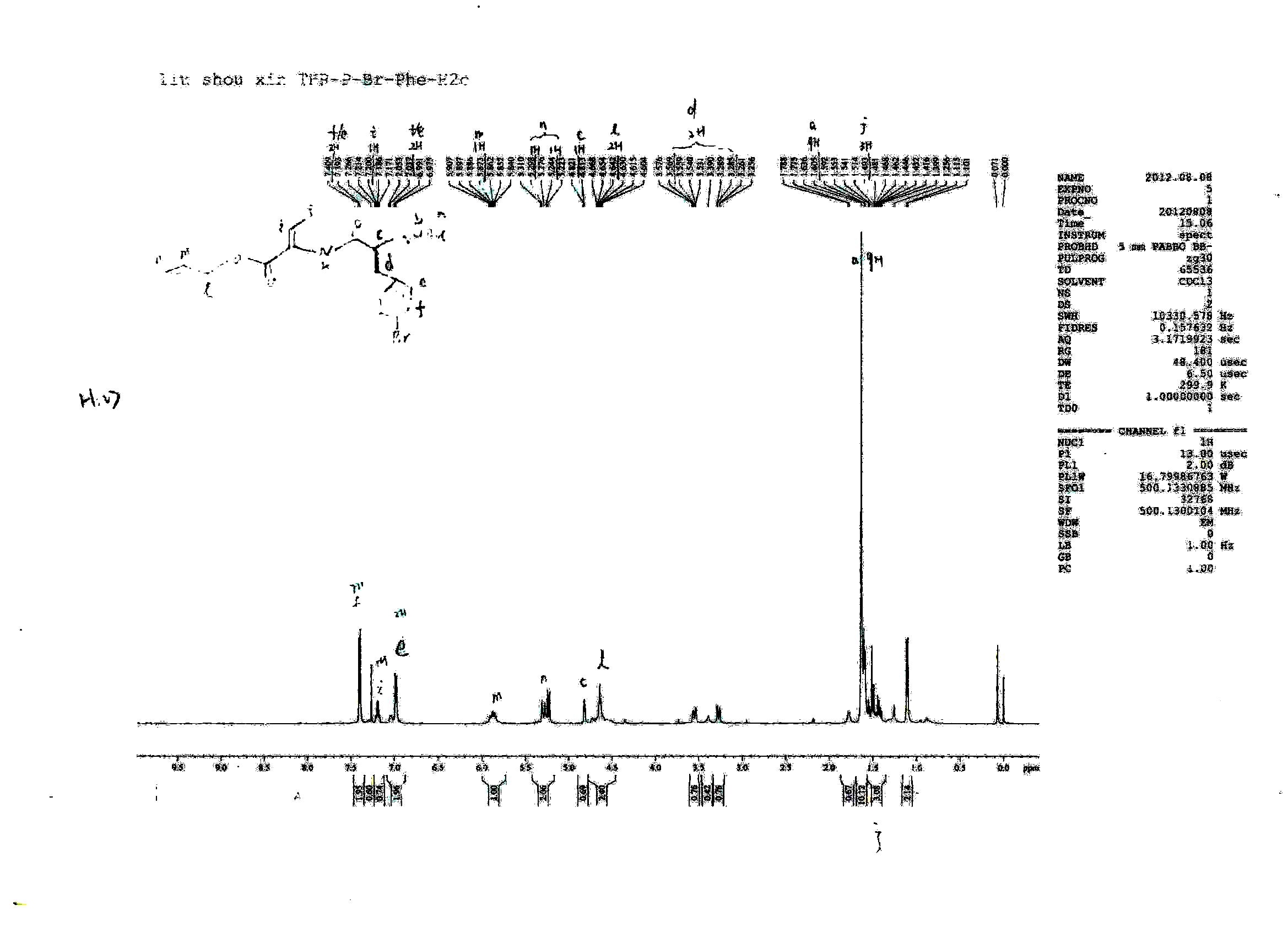
*

Figure S18 .^1^H NMR spectrum of *Boc- p-Br L-Phe-Z-ΔAbu-OAllyl (****6e****)*


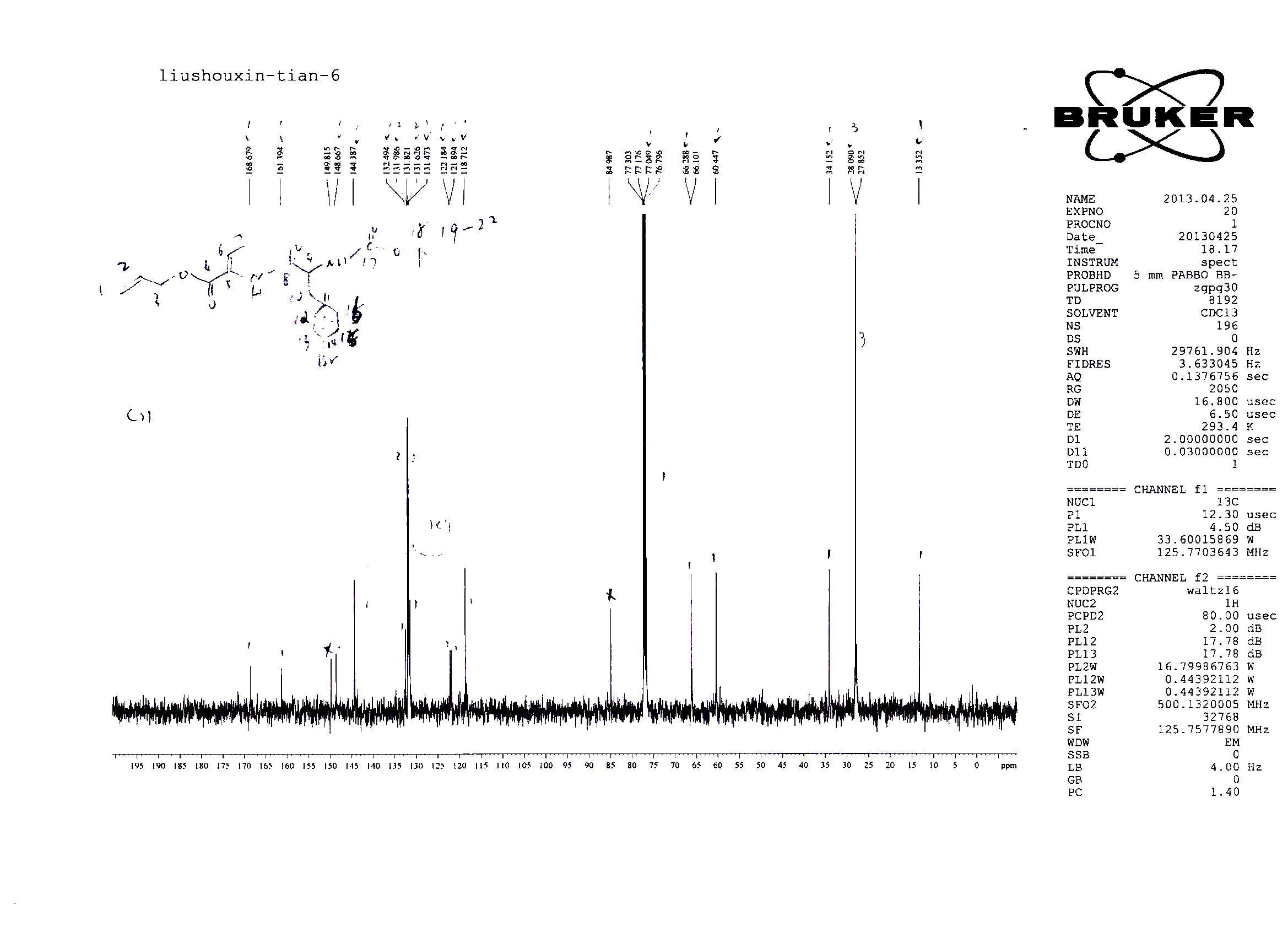


Figure S19 .CNMR spectrum of *Boc- p-Br L-Phe-Z-ΔAbu-OAllyl (****6e****)*

*
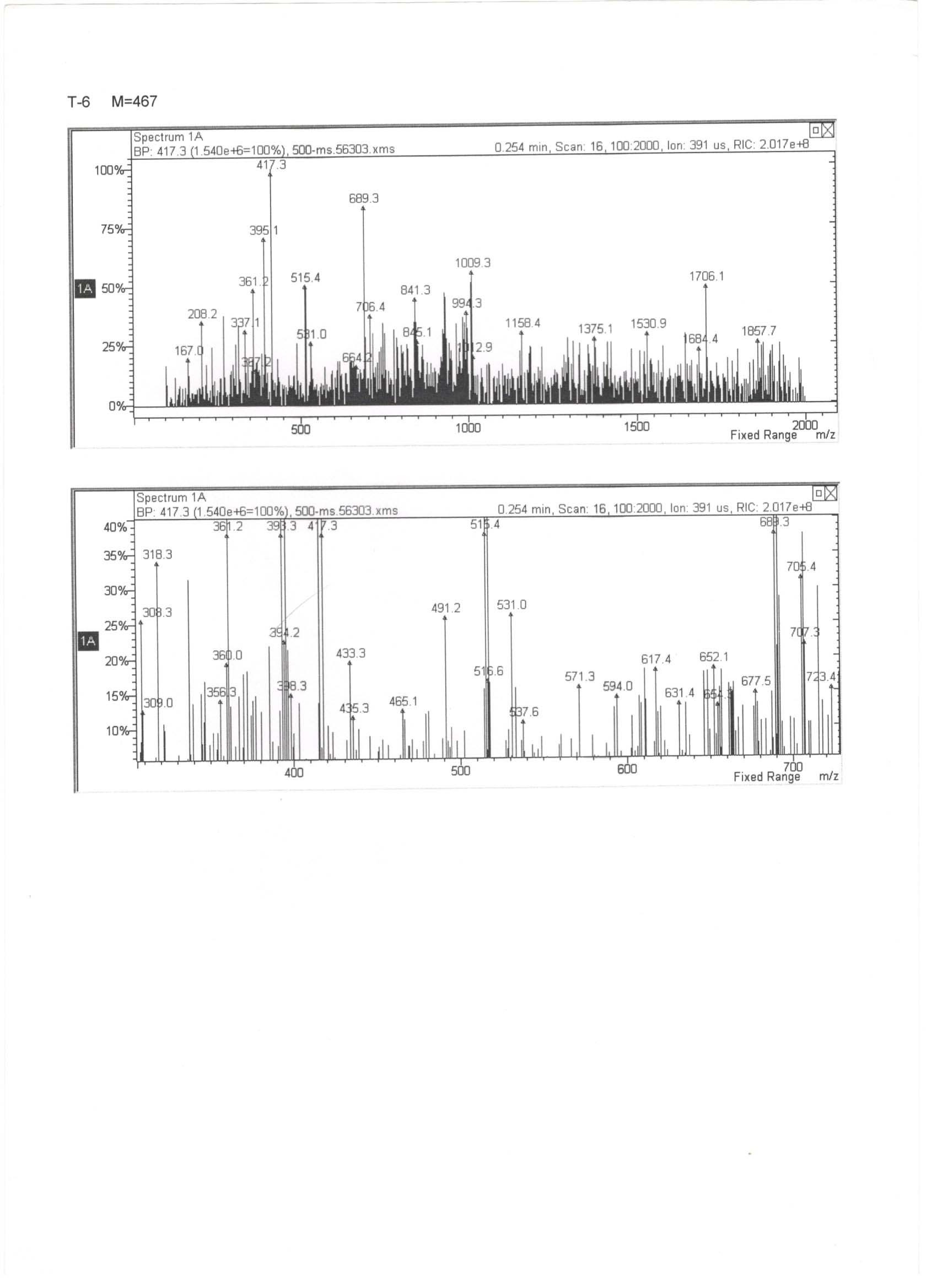
*

Figure S20 .MS spectrum of *Boc- p-Br L-Phe-Z-ΔAbu-OAllyl (****6e****)*

*Boc-L-Pro-L-Thr-OAllyl (5f)*

*
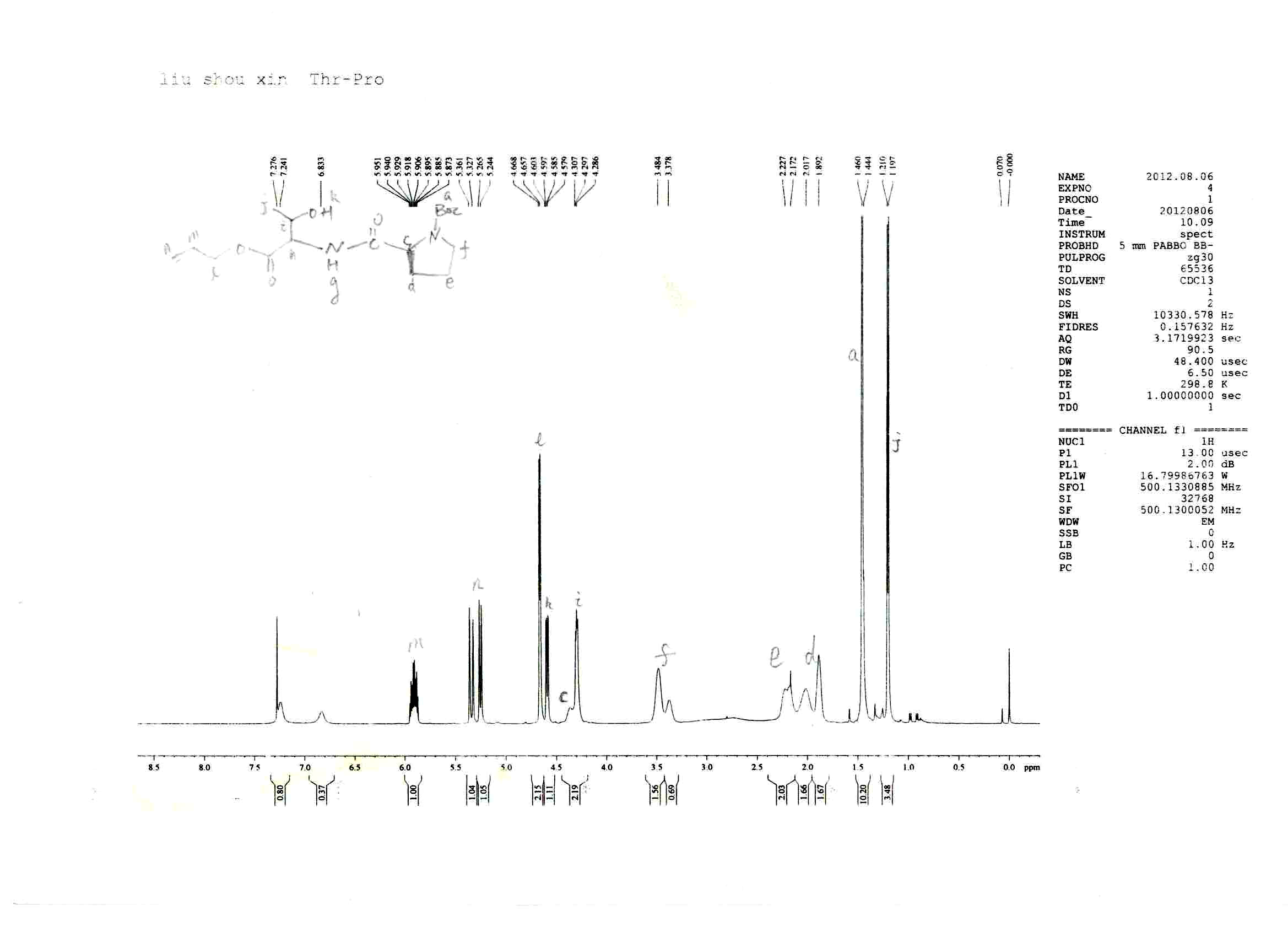
*

Figure S21 .^1^H NMR spectrum of *Boc-L-Pro-L-Thr-OAllyl (5f)*

*Boc-L-Pro-Z-ΔAbu-OAllyl (6f)*

*
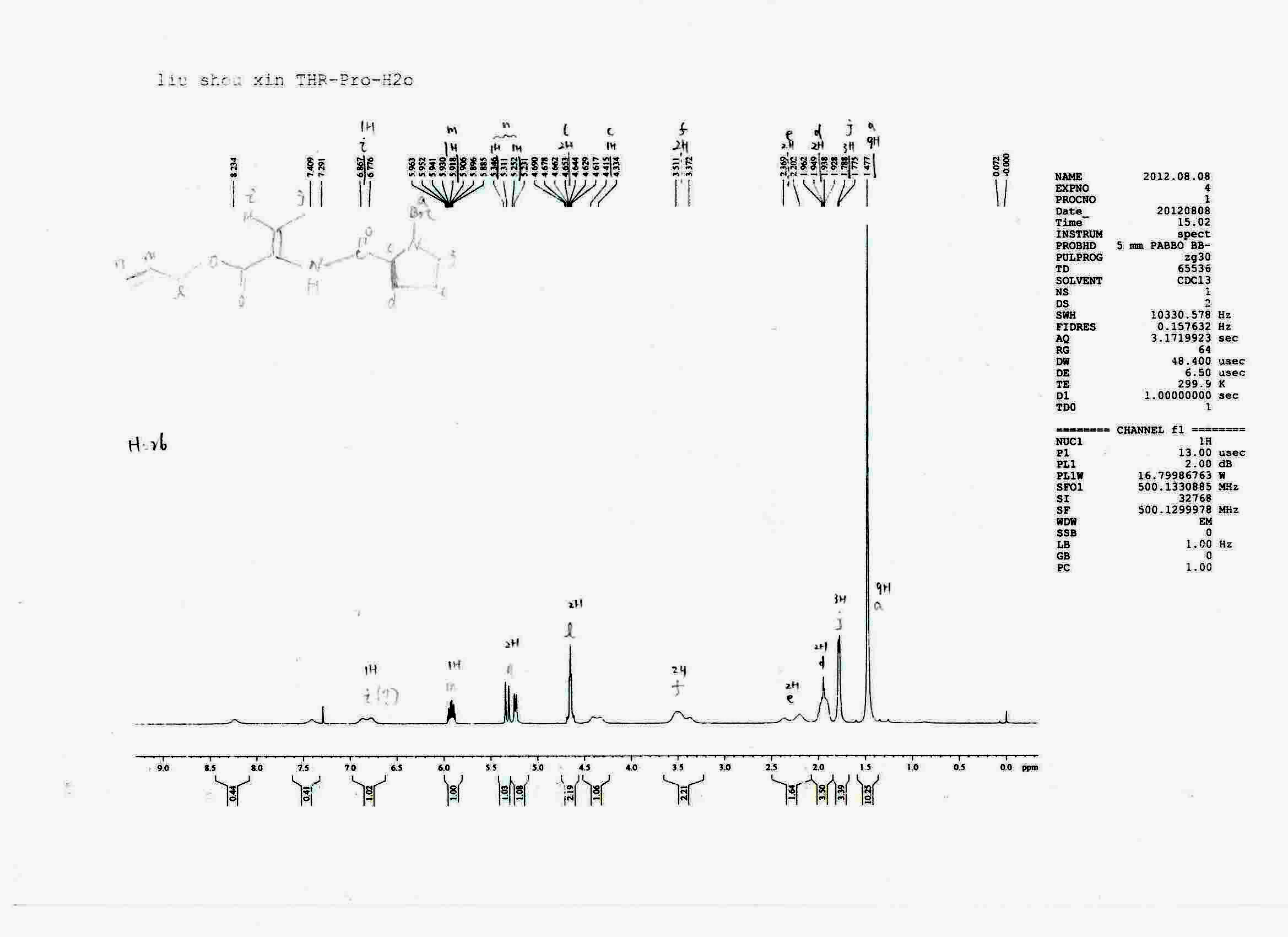
*

Figure S22 .^1^H NMR spectrum of *Boc-L-Pro-Z-ΔAbu-OAllyl (****6f****)*

*
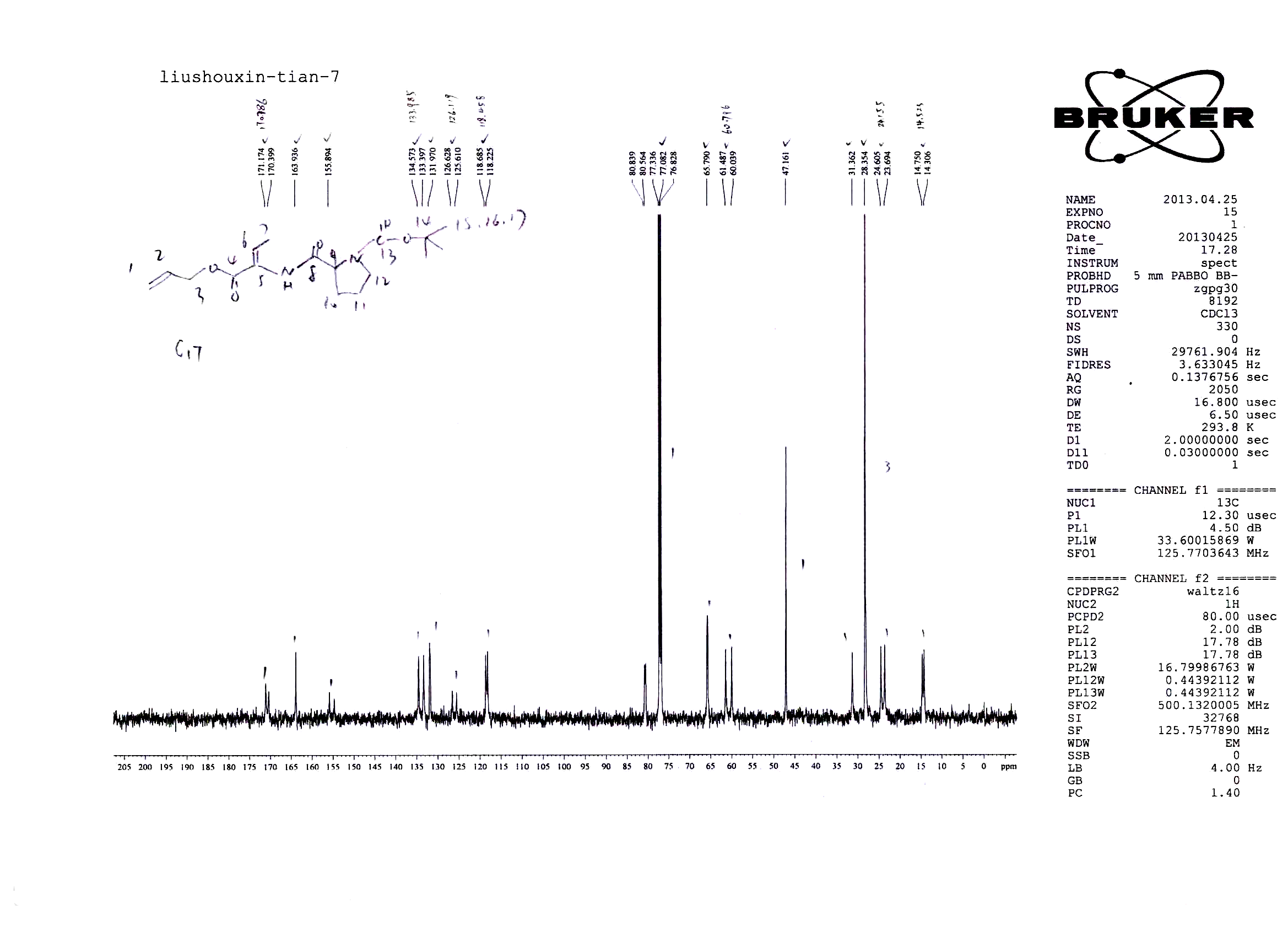
*

Figure S23 .CNMR spectrum of *Boc-L-Pro-Z-ΔAbu-OAllyl (****6f****)*

*
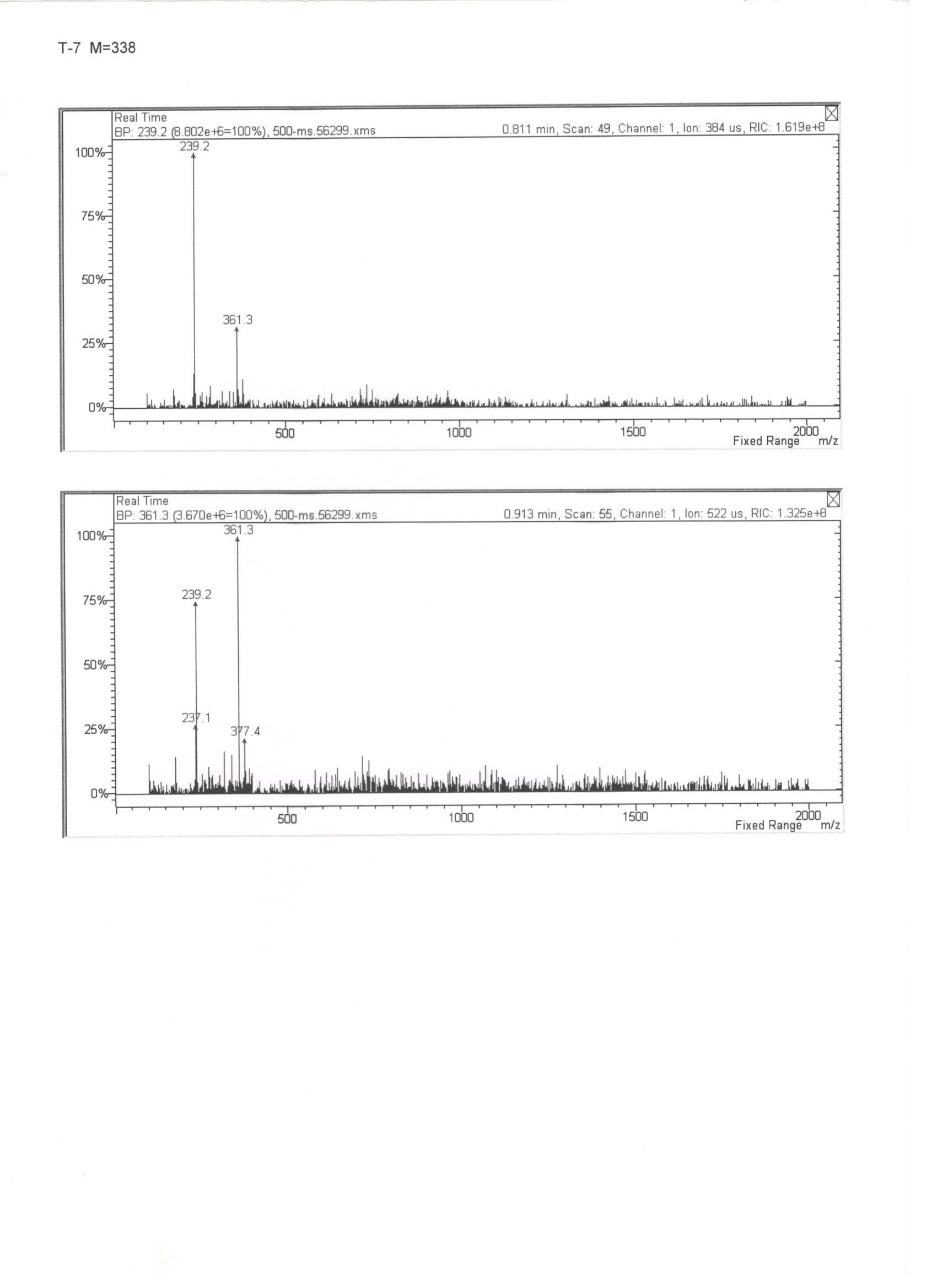
*

Figure S24 .MS spectrum of *Boc-L-Pro-Z-ΔAbu-OAllyl (****6f****)*

*Boc-L-His-L-Thr-OAllyl (5g)*

*
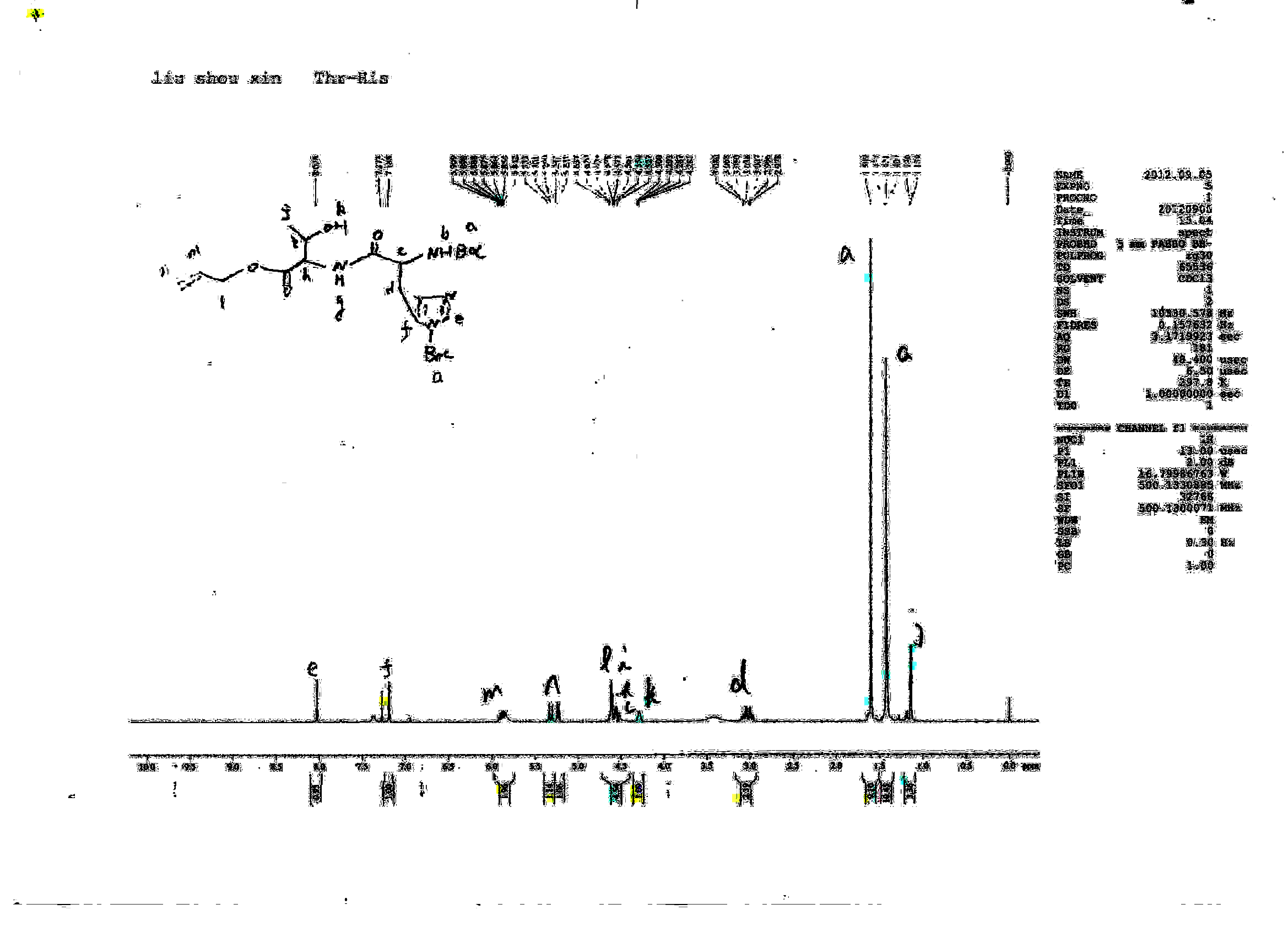
*

Figure S25 .^1^H NMR spectrum of *Boc-L-His-L-Thr-OAllyl (5g)*

*Boc-L-His-Z-ΔAbu-OAllyl (6g)*

*
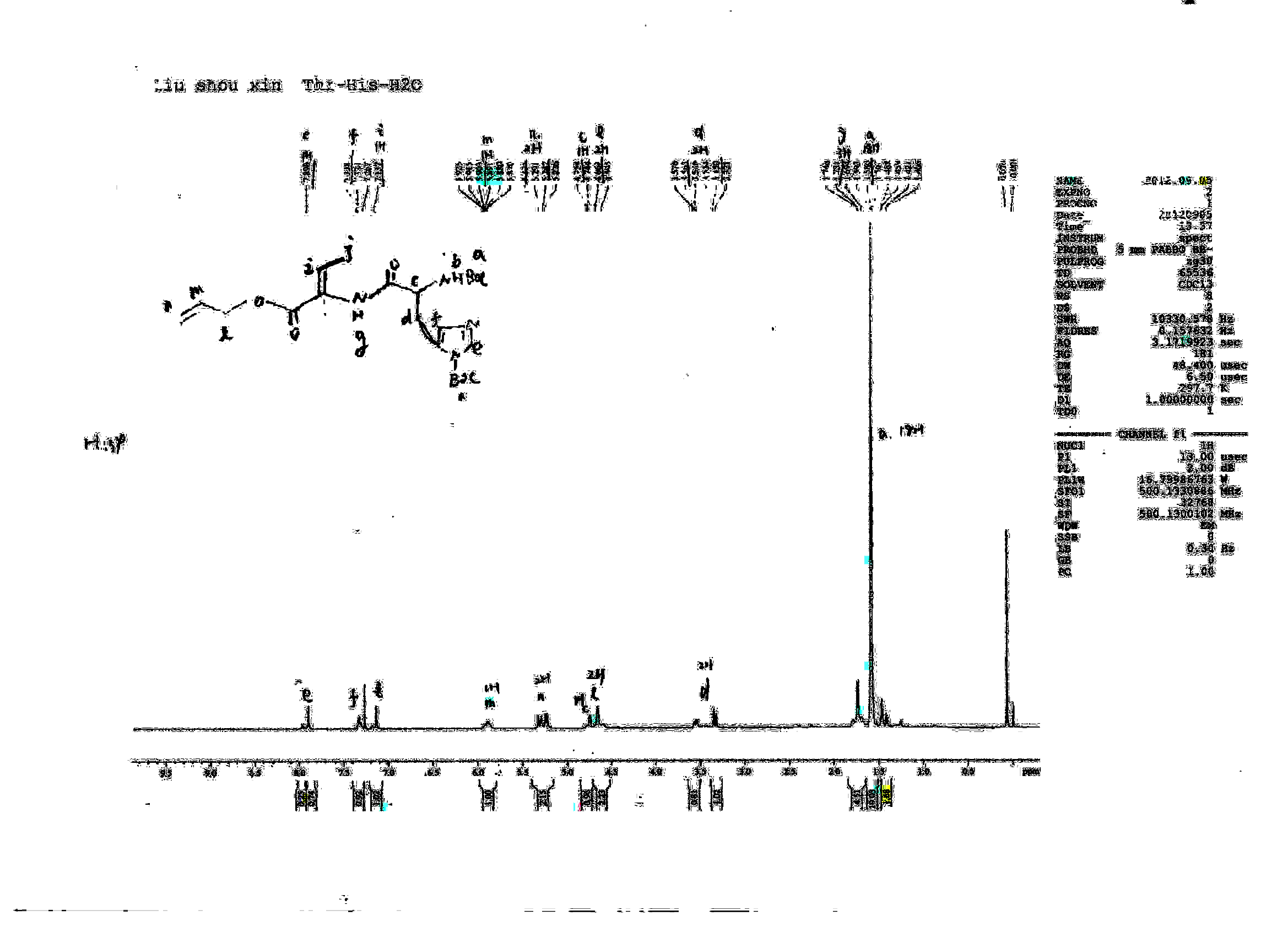
*

Figure S26 .^1^H NMR spectrum of *Boc-L-His-Z-ΔAbu-OAllyl (****6g****)*

*
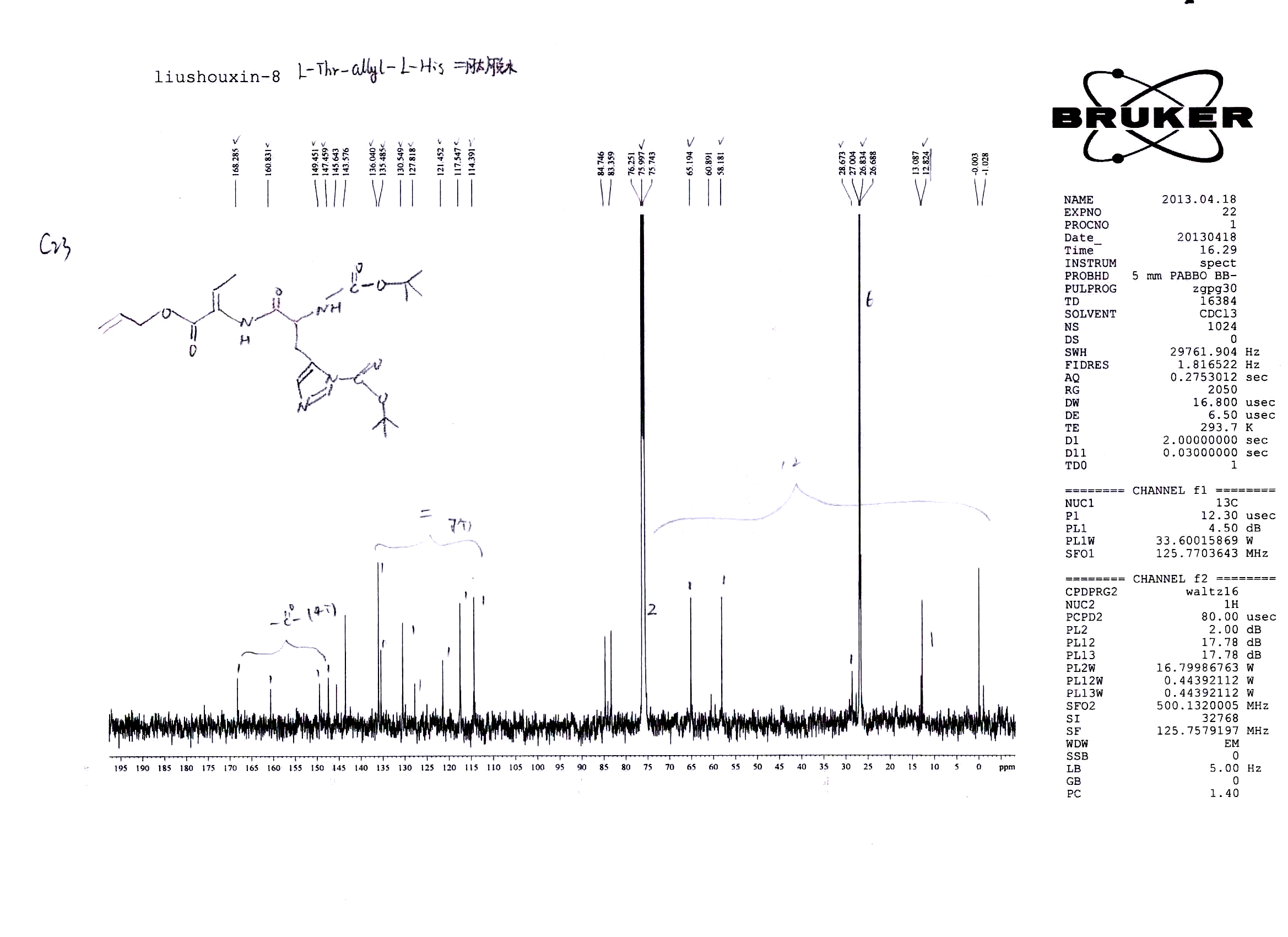
*

Figure S27 .CNMR spectrum of *Boc-L-His-Z-ΔAbu-OAllyl (****6g****)*

*
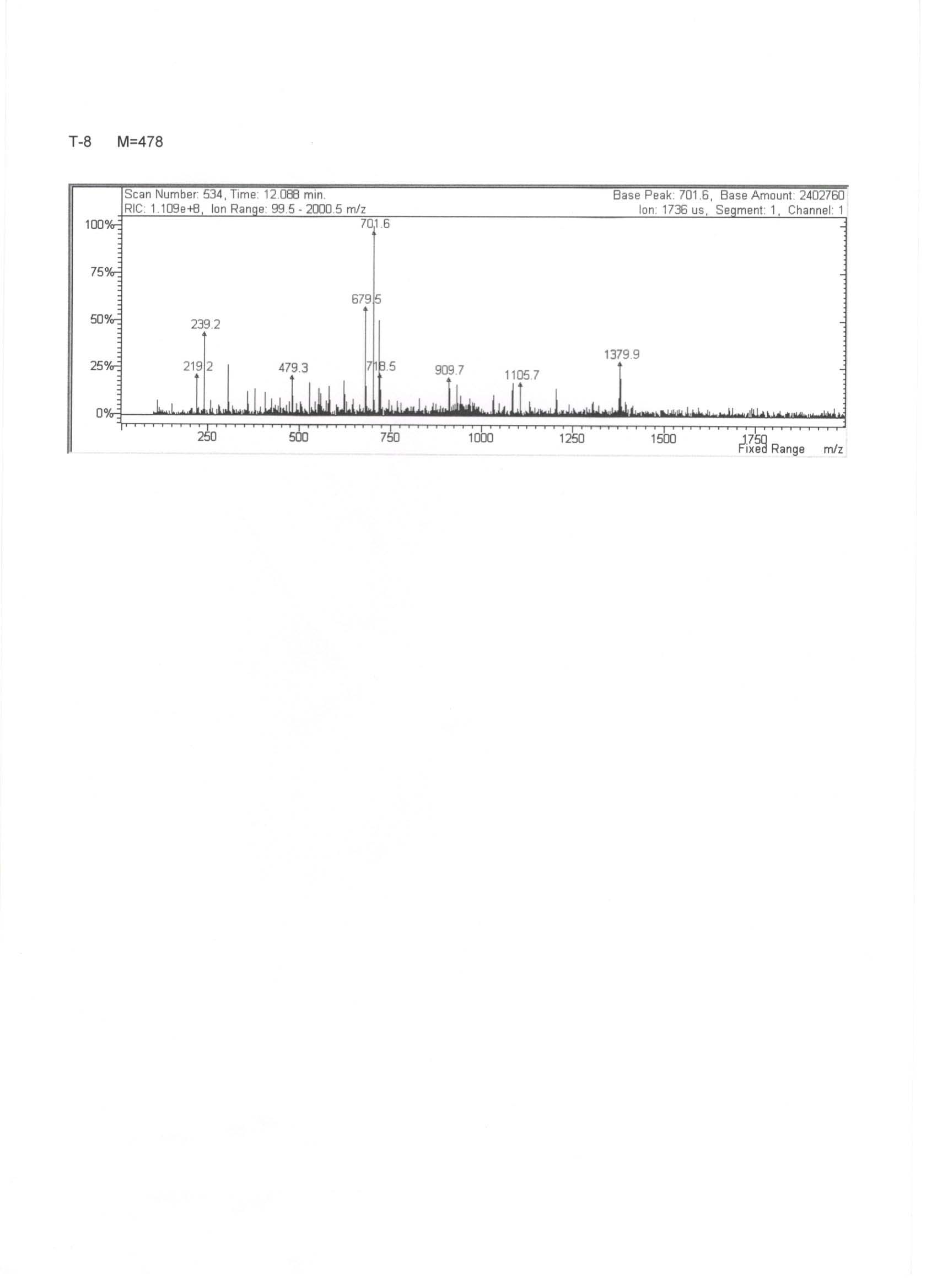
*

Figure S28 .MS spectrum of *Boc-L-His-Z-ΔAbu-OAllyl (****6g****)*

*Boc-L-Phe-L-Thr-OAllyl (5h)*


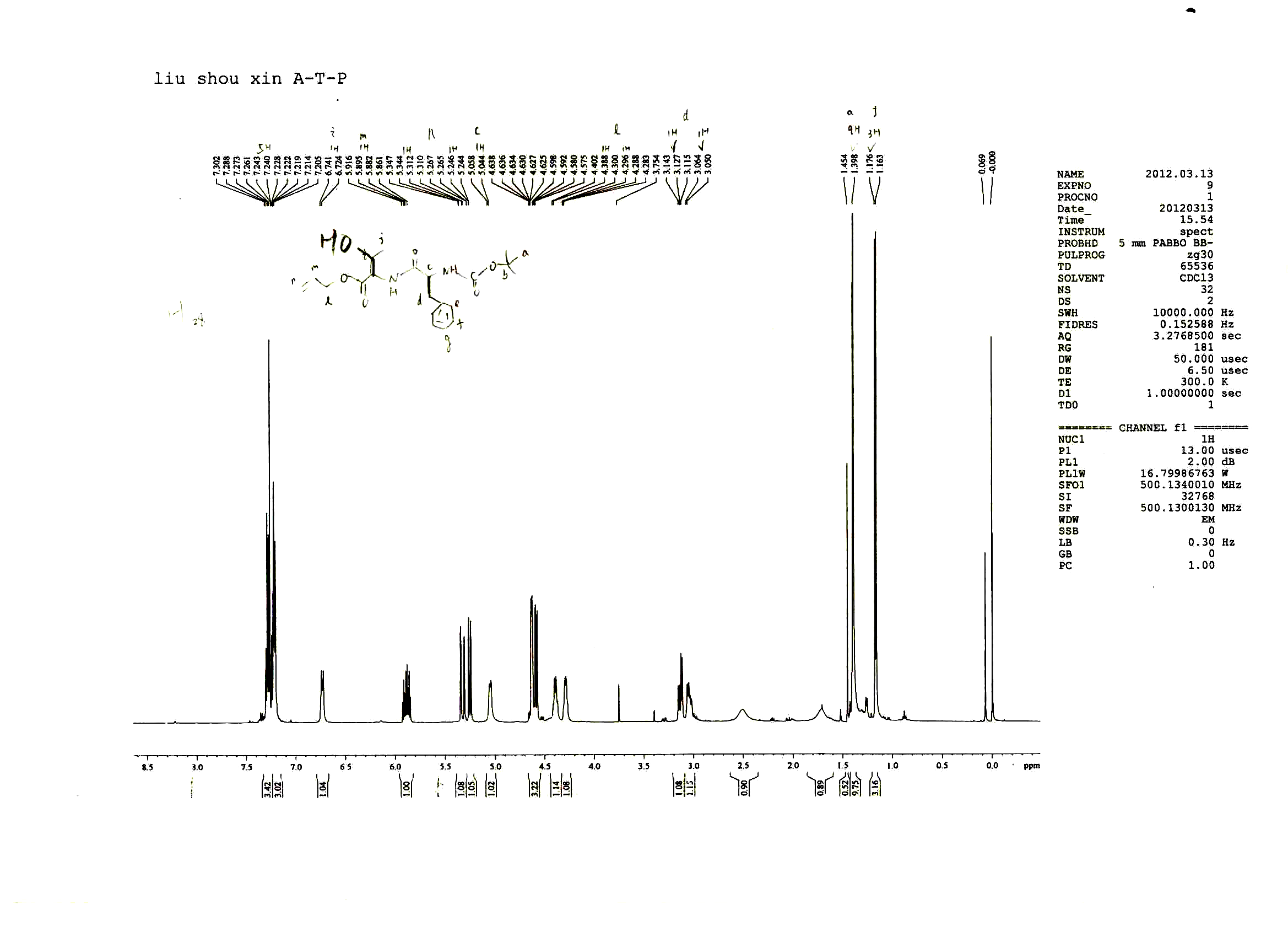


Figure S29 .^1^H NMR spectrum of *Boc-L-Phe-L-Thr-OAllyl (5h)*

*Boc-L-Phe-Z-ΔAbu-OAllyl (6h)*

*
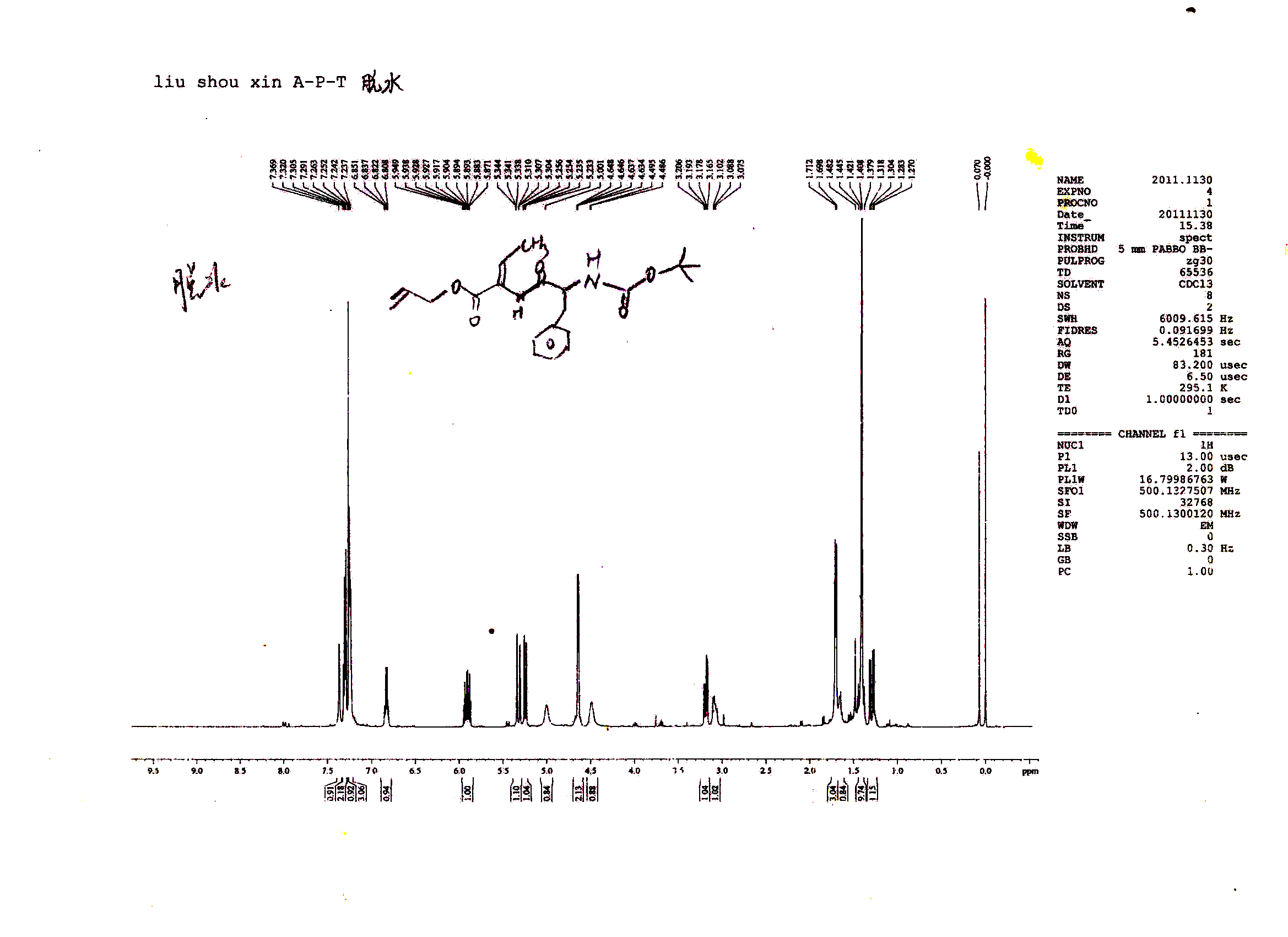
*

Figure S30 .^1^H NMR spectrum of *Boc-L-Phe-Z-ΔAbu-OAllyl (****6h****)*

*
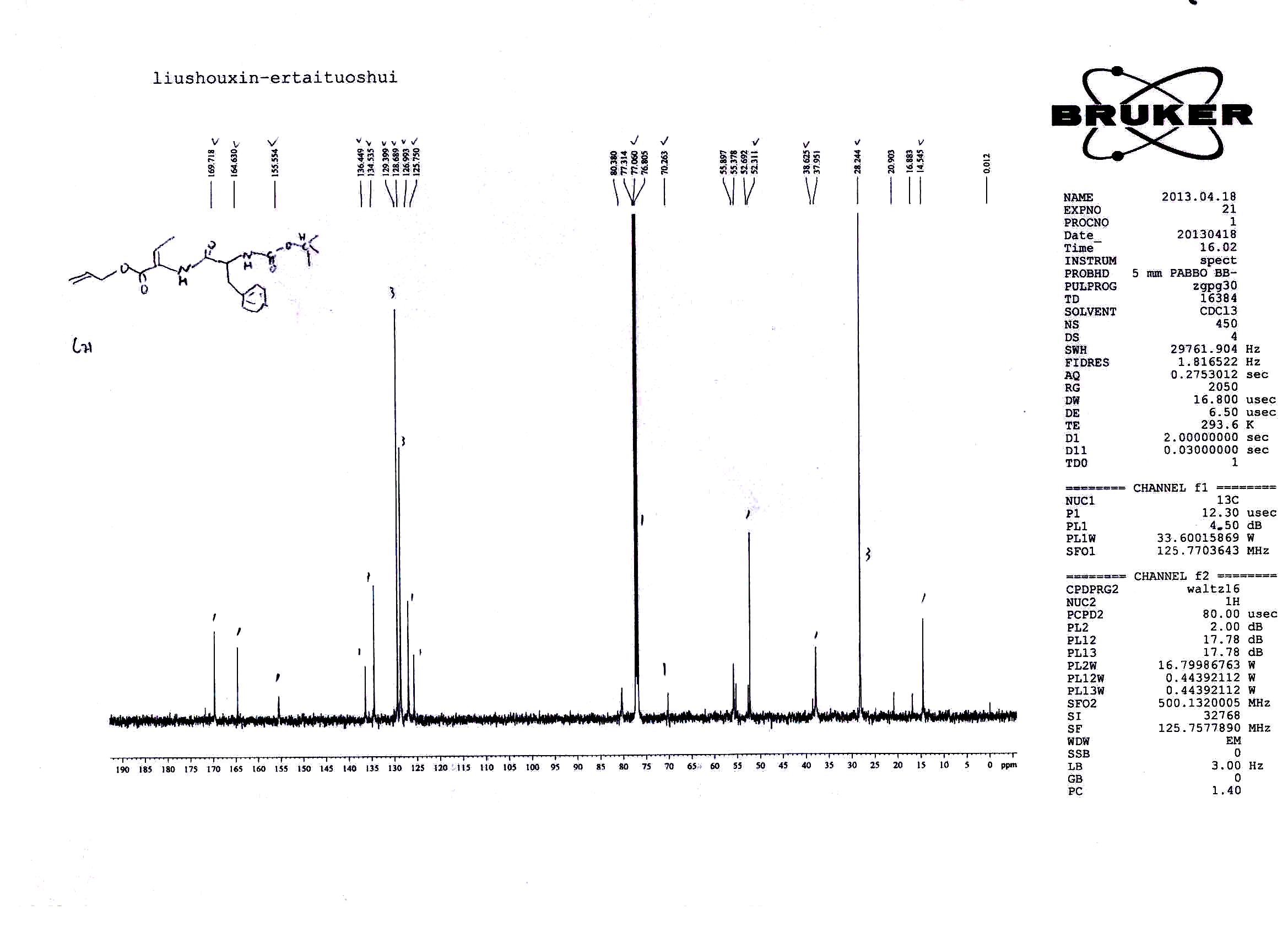
*

Figure S31 .CNMR spectrum of *Boc-L-Phe-Z-ΔAbu-OAllyl (****6h****)*

*Boc-L-Tyr-L-Thr-OAllyl (5i)*

*
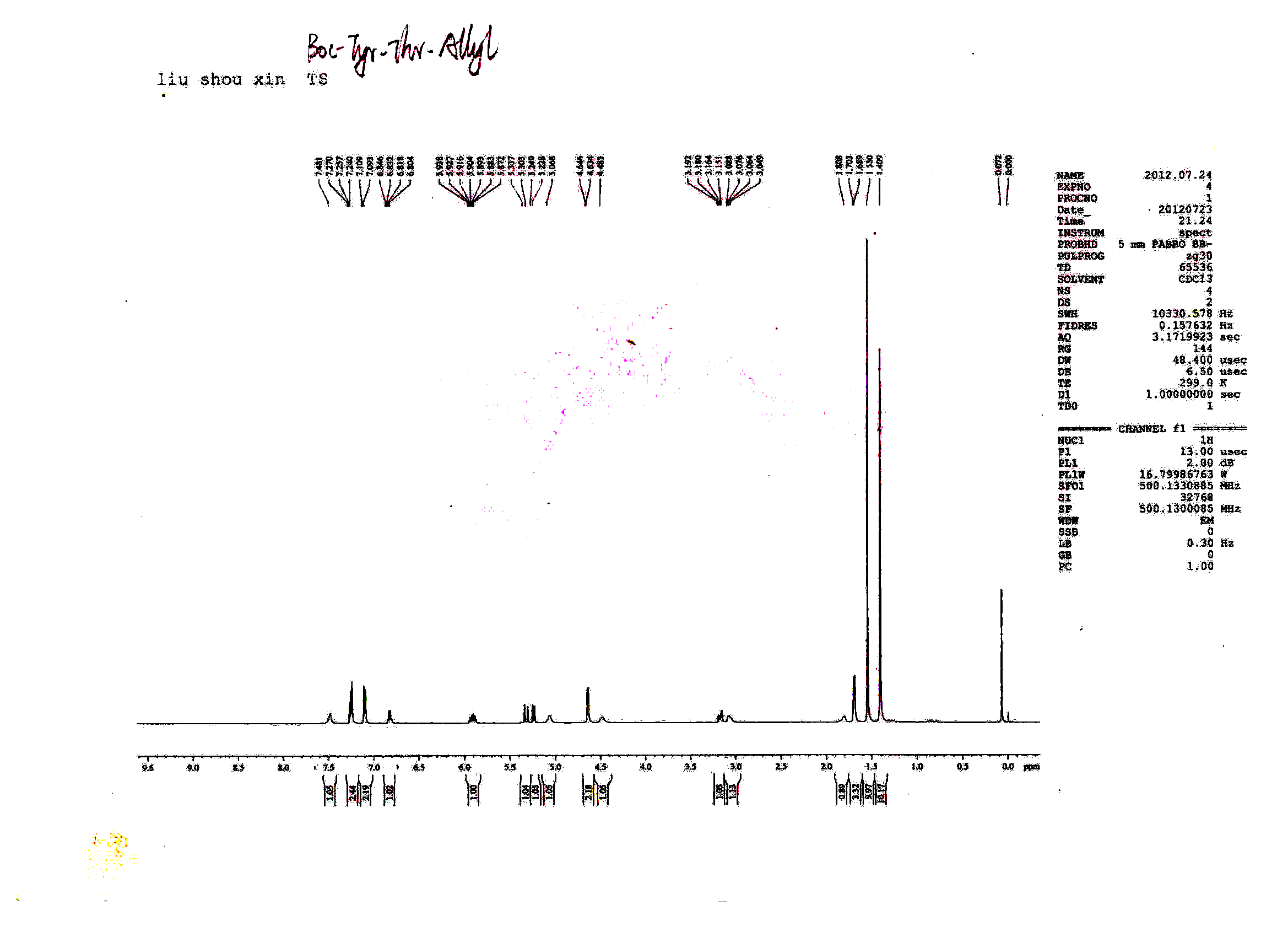
*

Figure S32 .^1^H NMR spectrum of *Boc-L-Tyr-L-Thr-OAllyl (5i)*

*Boc-L-Tyr-Z-ΔAbu-OAllyl (6i)*

*
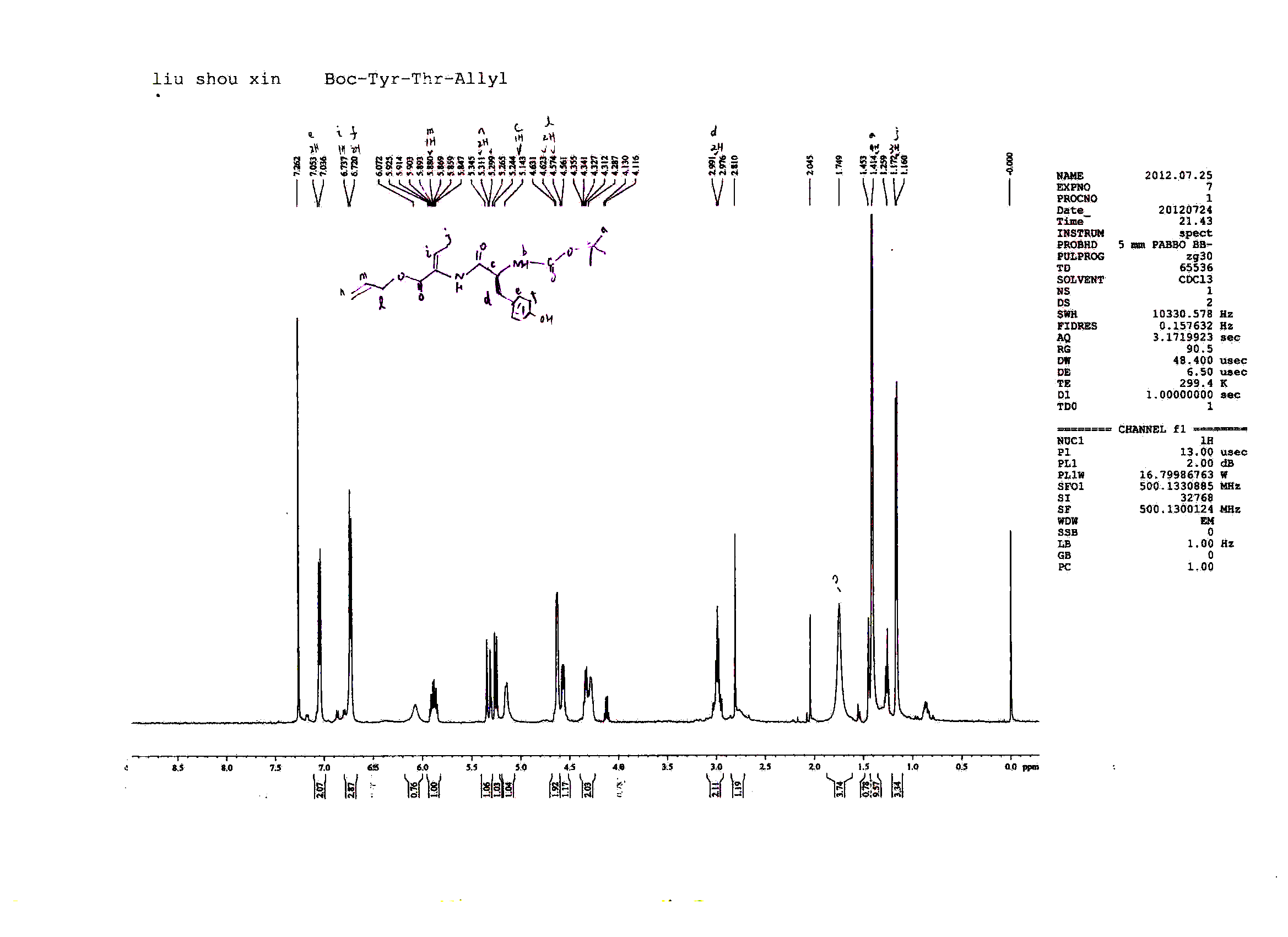
*

Figure S33 .^1^H NMR spectrum of *Boc-L-Tyr-Z-ΔAbu-OAllyl (****6i****)*


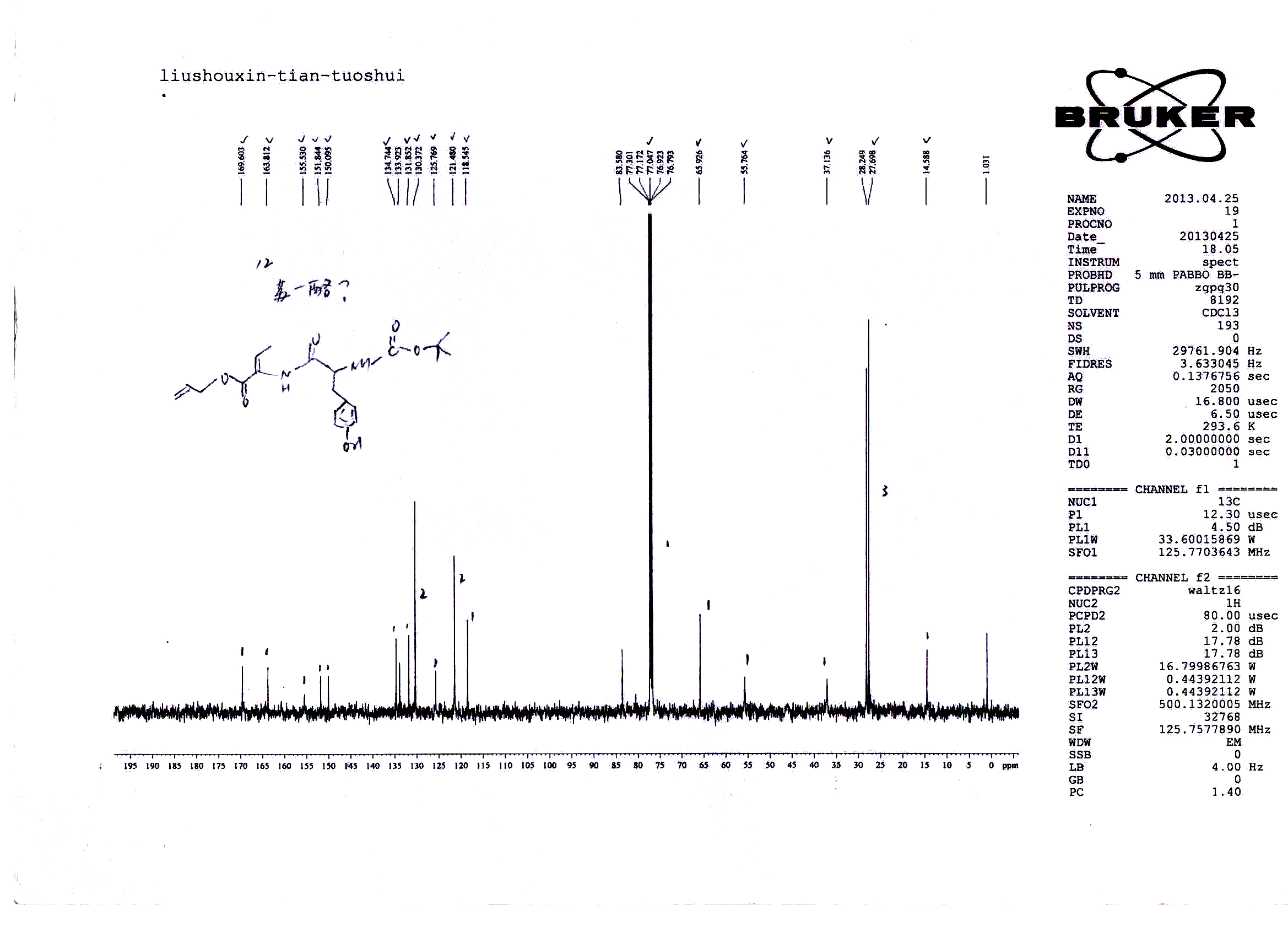


Figure S34 .CNMR spectrum of *Boc-L-Tyr-Z-ΔAbu-OAllyl (****6i****)*

*
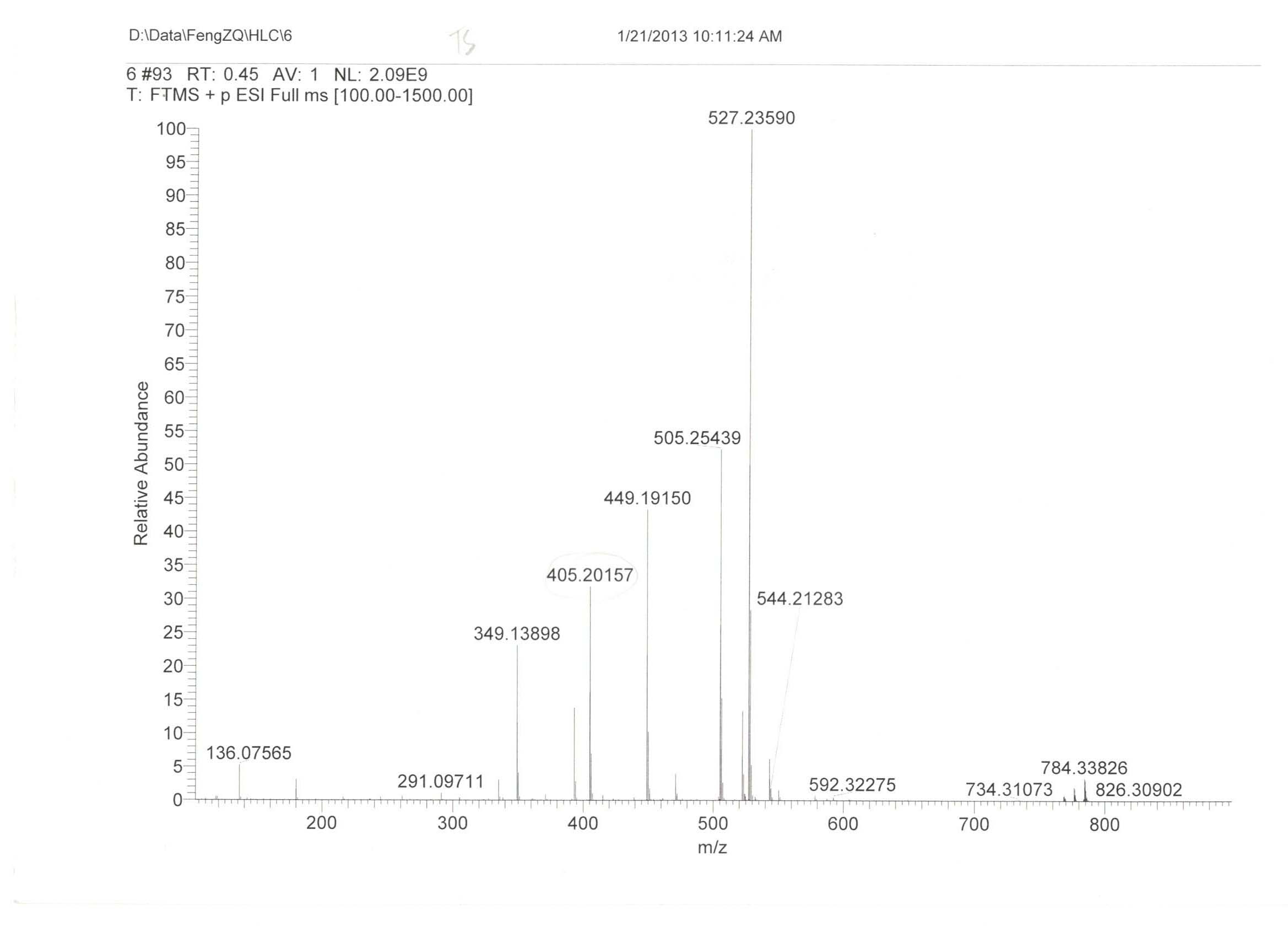
*

Figure S35 .MS spectrum of *Boc-L-Tyr-Z-ΔAbu-OAllyl (****6i****)*

*Boc-L-β-Nap-L-Thr-OAllyl (5j)*

*
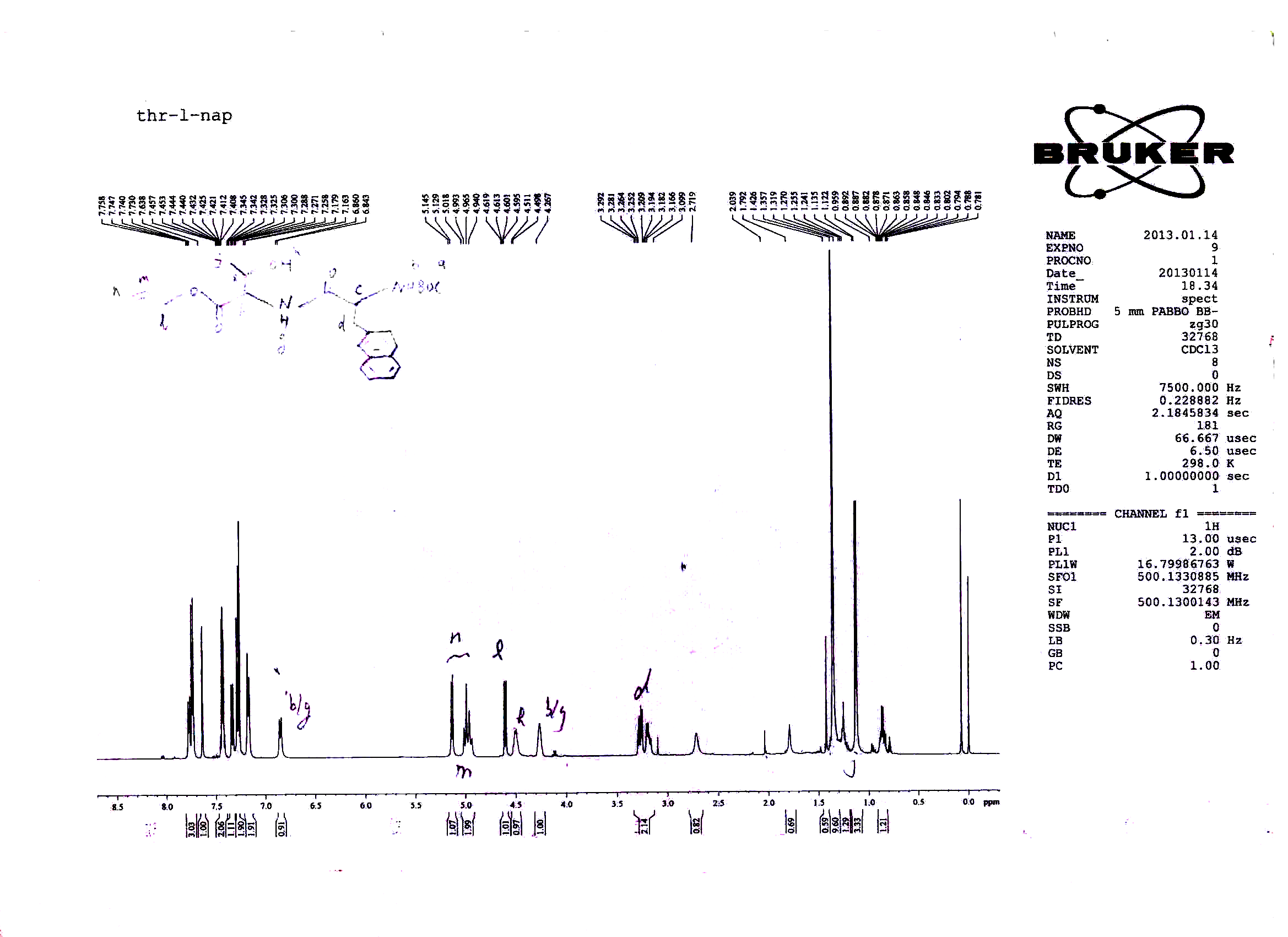
*

Figure S36 .^1^H NMR spectrum of *Boc-L-β-Nap-L-Thr-OAllyl (5j)*

*Boc-L-β-Nap-Z-Δabu–OAllyl (6j)*

*
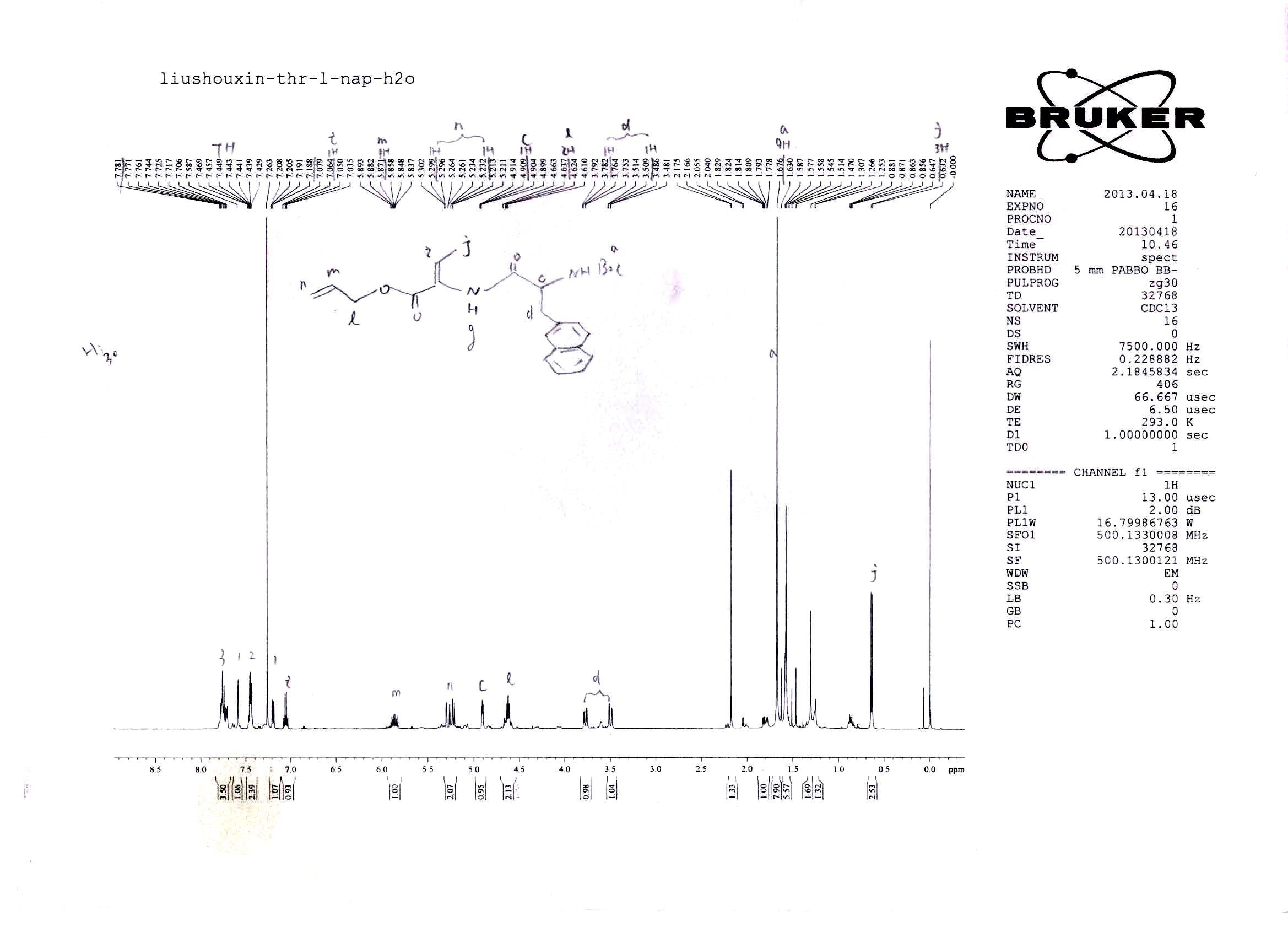
*

Figure S37 .^1^H NMR spectrum of *Boc-L-β-Nap-Z-Δabu–OAllyl (****6j****)*

*
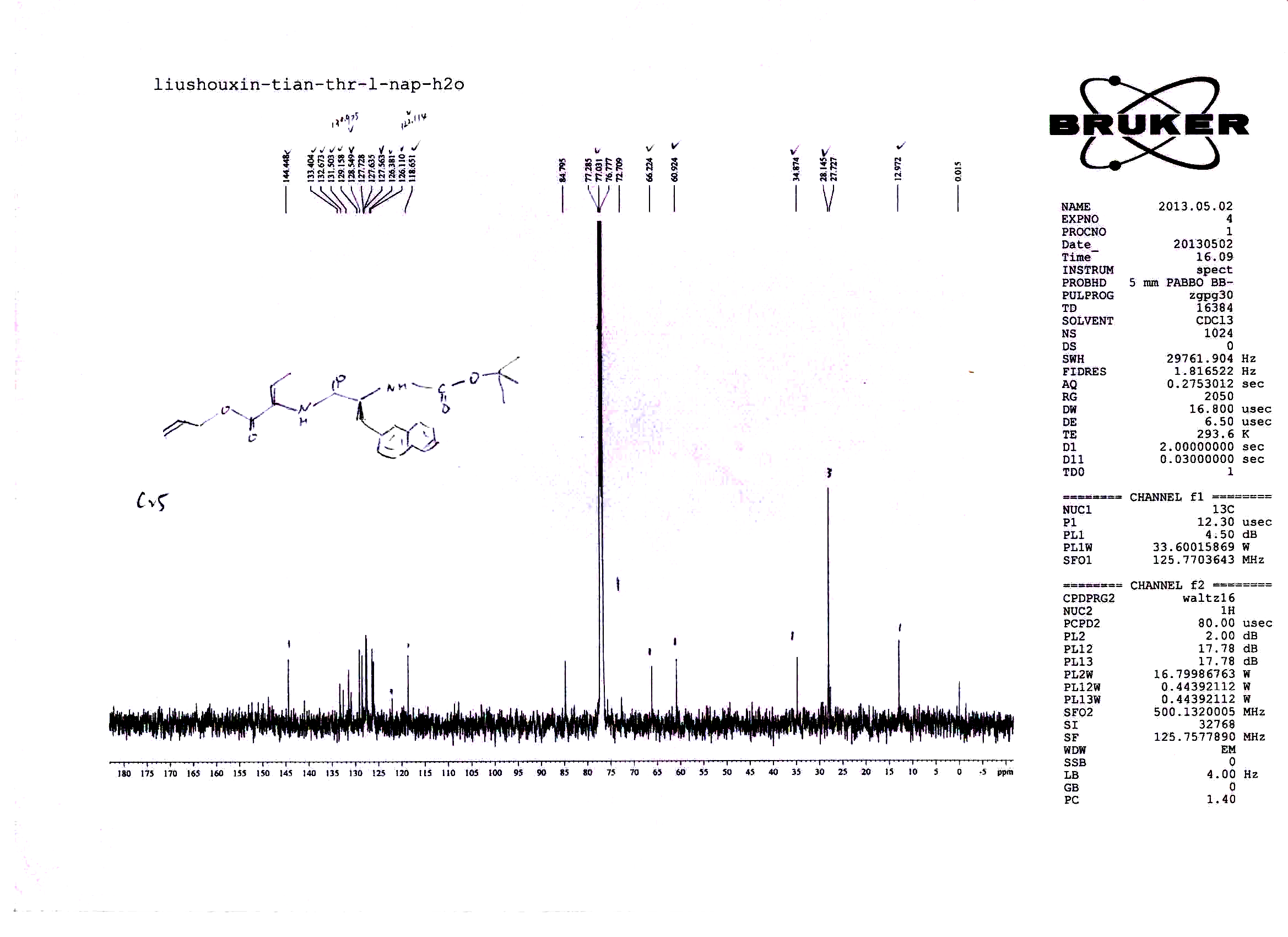
*

Figure S38 .CNMR spectrum of *Boc-L-β-Nap-Z-Δabu–OAllyl (****6j****)*


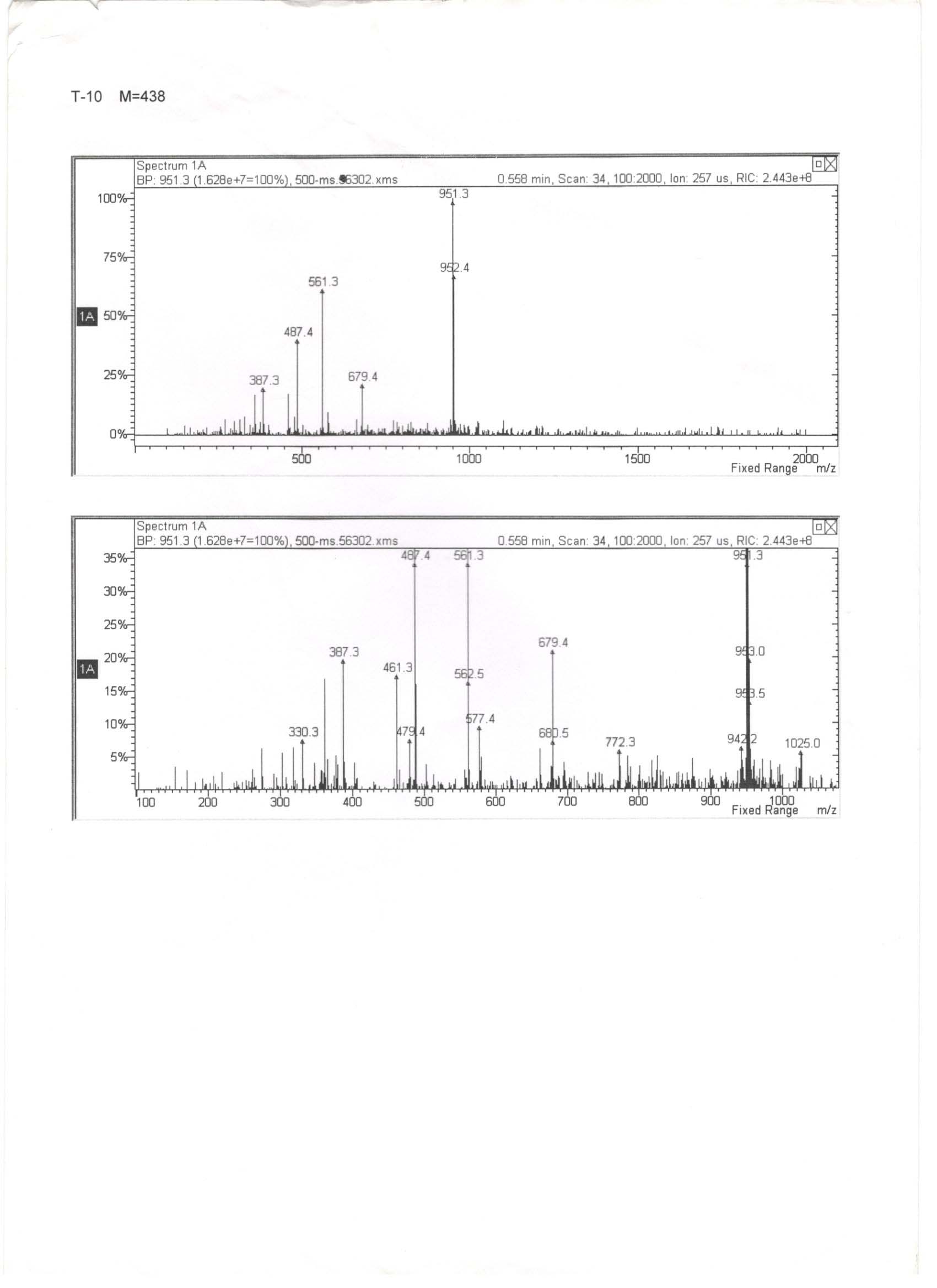


Figure S39 .MS spectrum of *Boc-L-β-Nap-Z-Δabu–OAllyl (****6j****)*

*Boc-D-Leu-L-Thr-OAllyl (5k)*

*
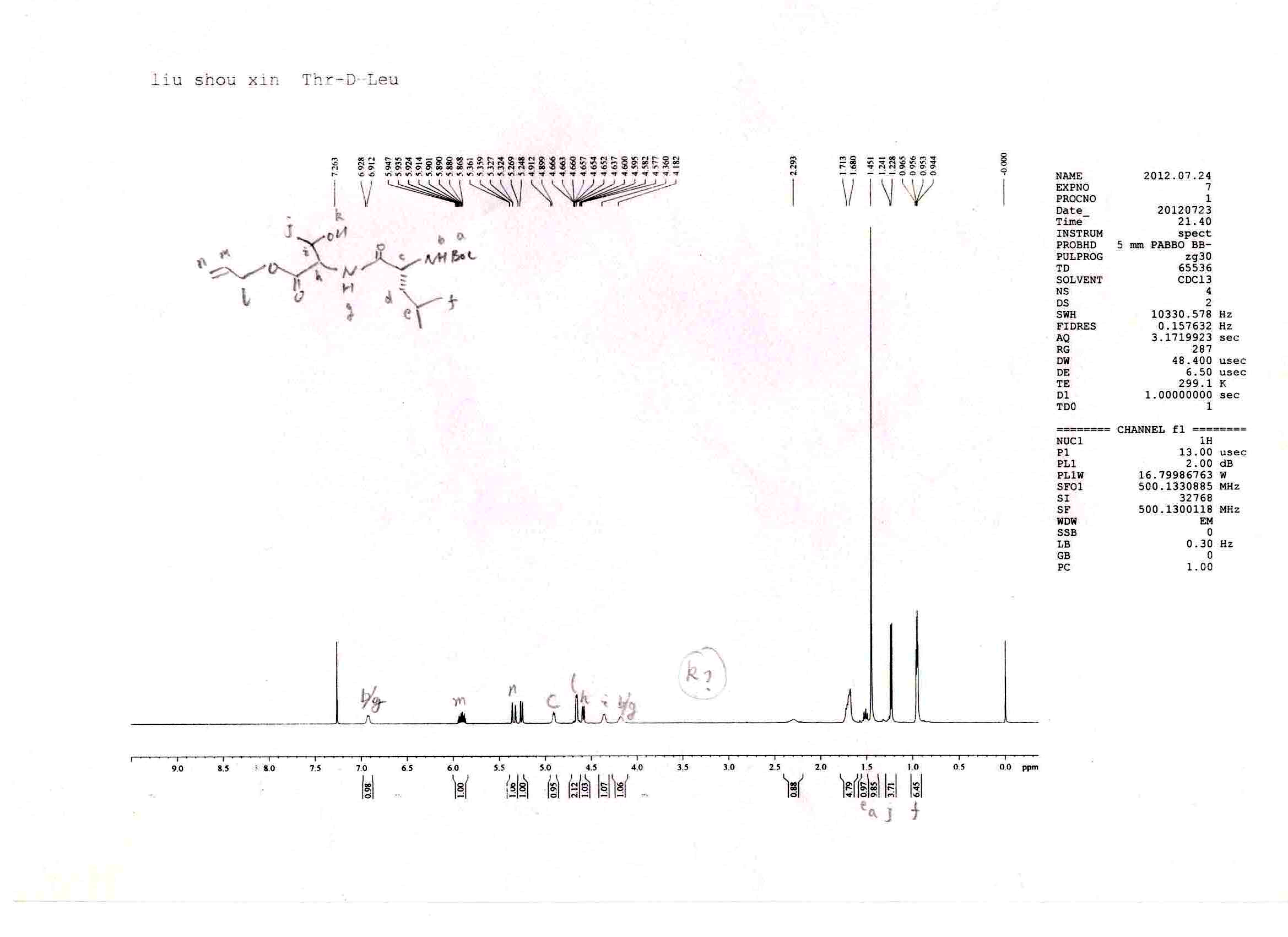
*

Figure S40 .^1^H NMR spectrum of *Boc-D-Leu-L-Thr-OAllyl (5k)*

*Boc-D-Leu-Z-ΔAbu-OAllyl (6k)*


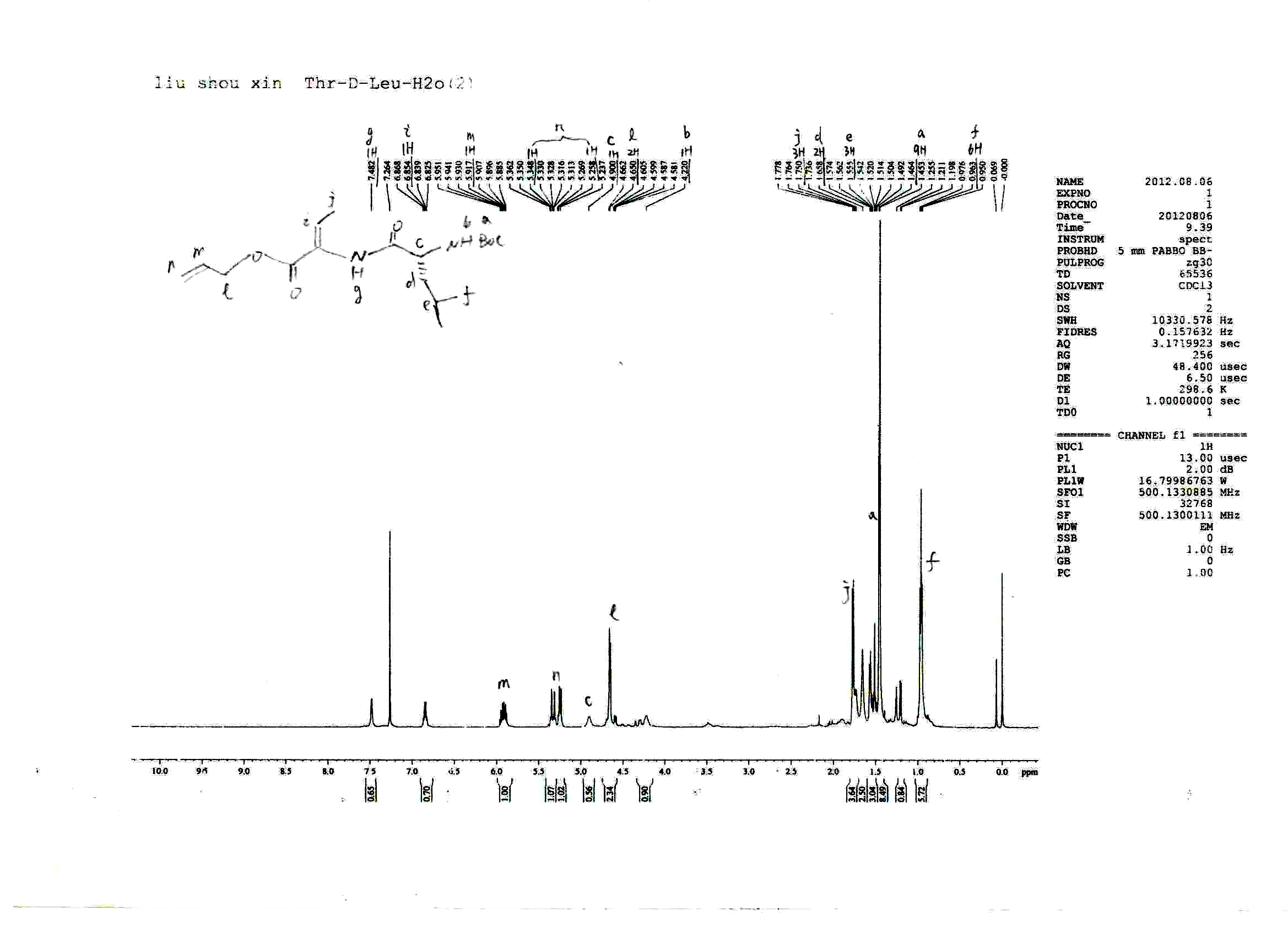


Figure S41 .^1^H NMR spectrum of *Boc-D-Leu-Z-ΔAbu-OAllyl (****6k****)*

*
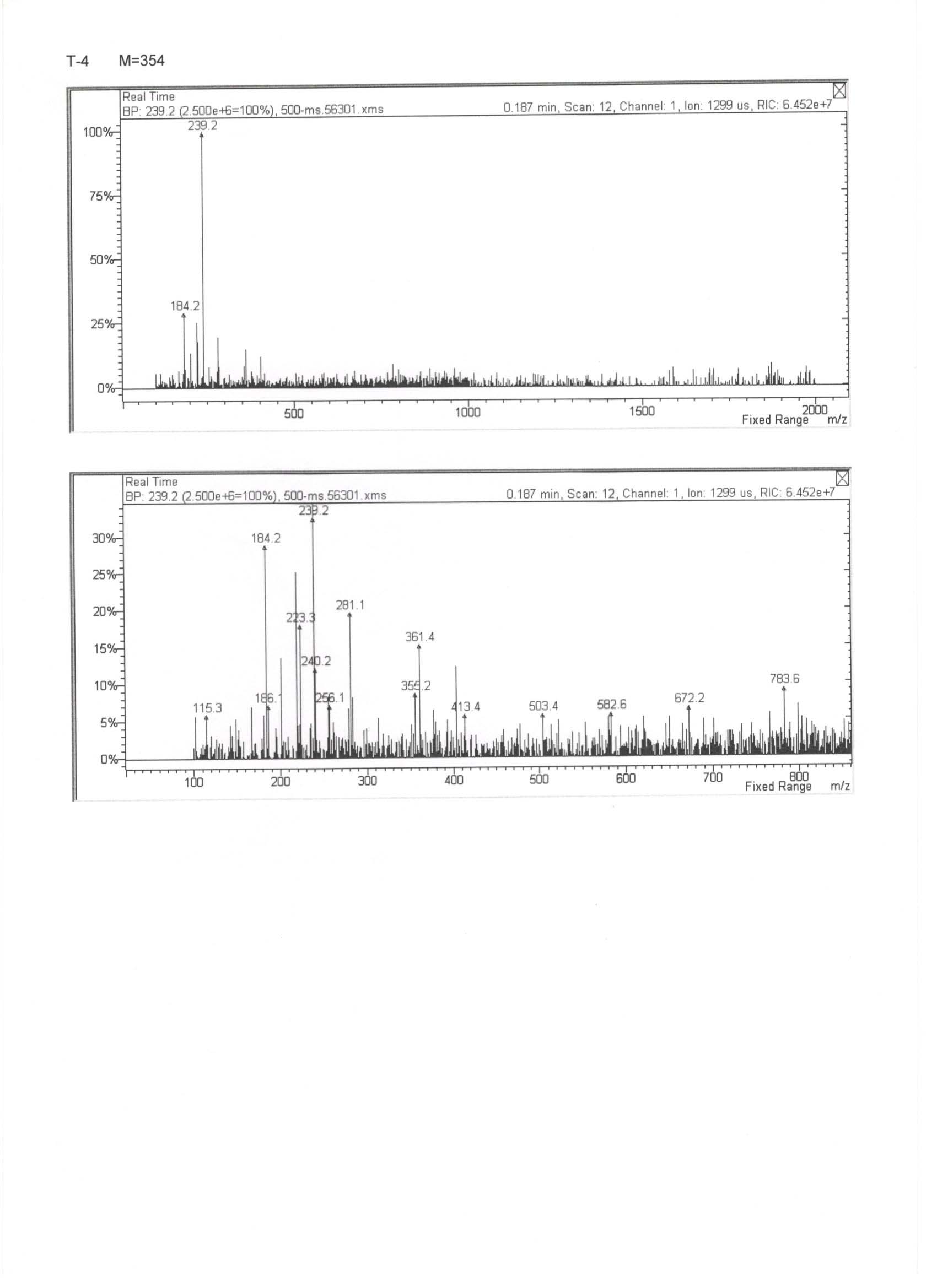
*

Figure S42 .MS spectrum of *Boc-D-Leu-Z-ΔAbu-OAllyl (****6k****)*

*Boc-D- β-Nap-L-Thr-OAllyl (5l)*

*
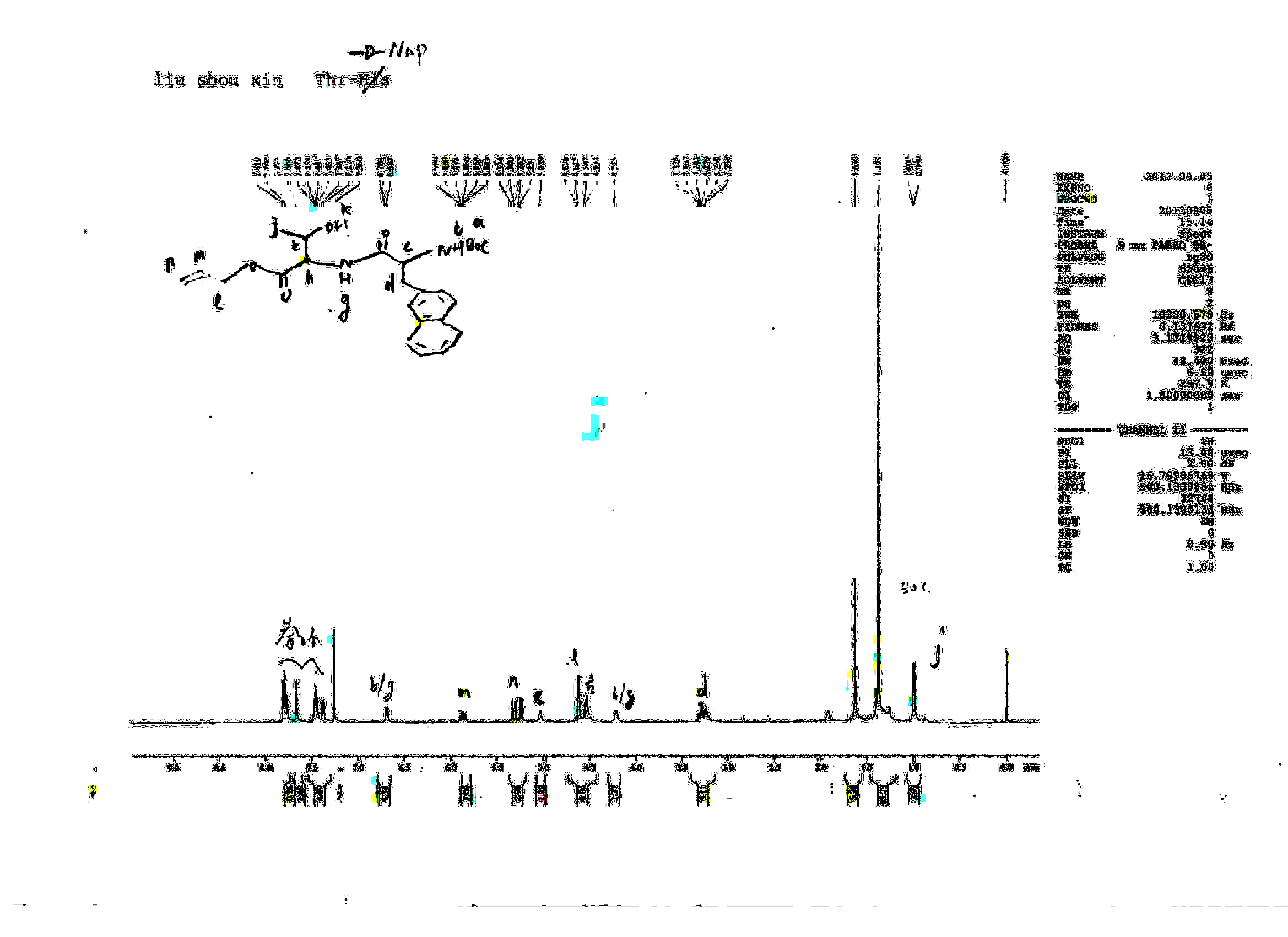
*

Figure S43 .^1^H NMR spectrum of *Boc-D- β-Nap-L-Thr-OAllyl (5l)*

*Boc-D-β-Nap-Z-Δabu–Oallyl (6l)*

*
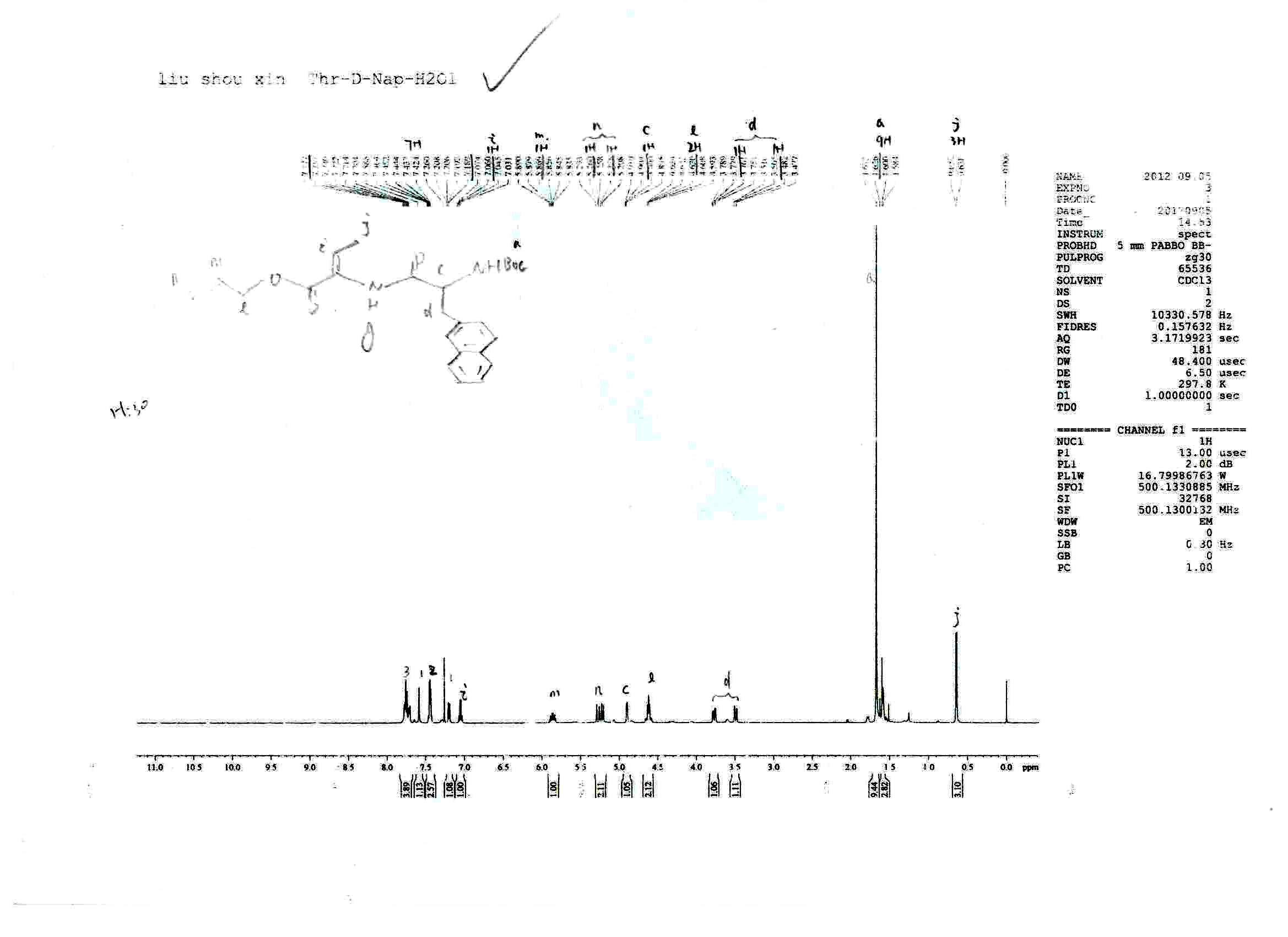
*

Figure S44 .^1^H NMR spectrum of *Boc-D-β-Nap-Z-Δabu–Oallyl (****6l****)*

*
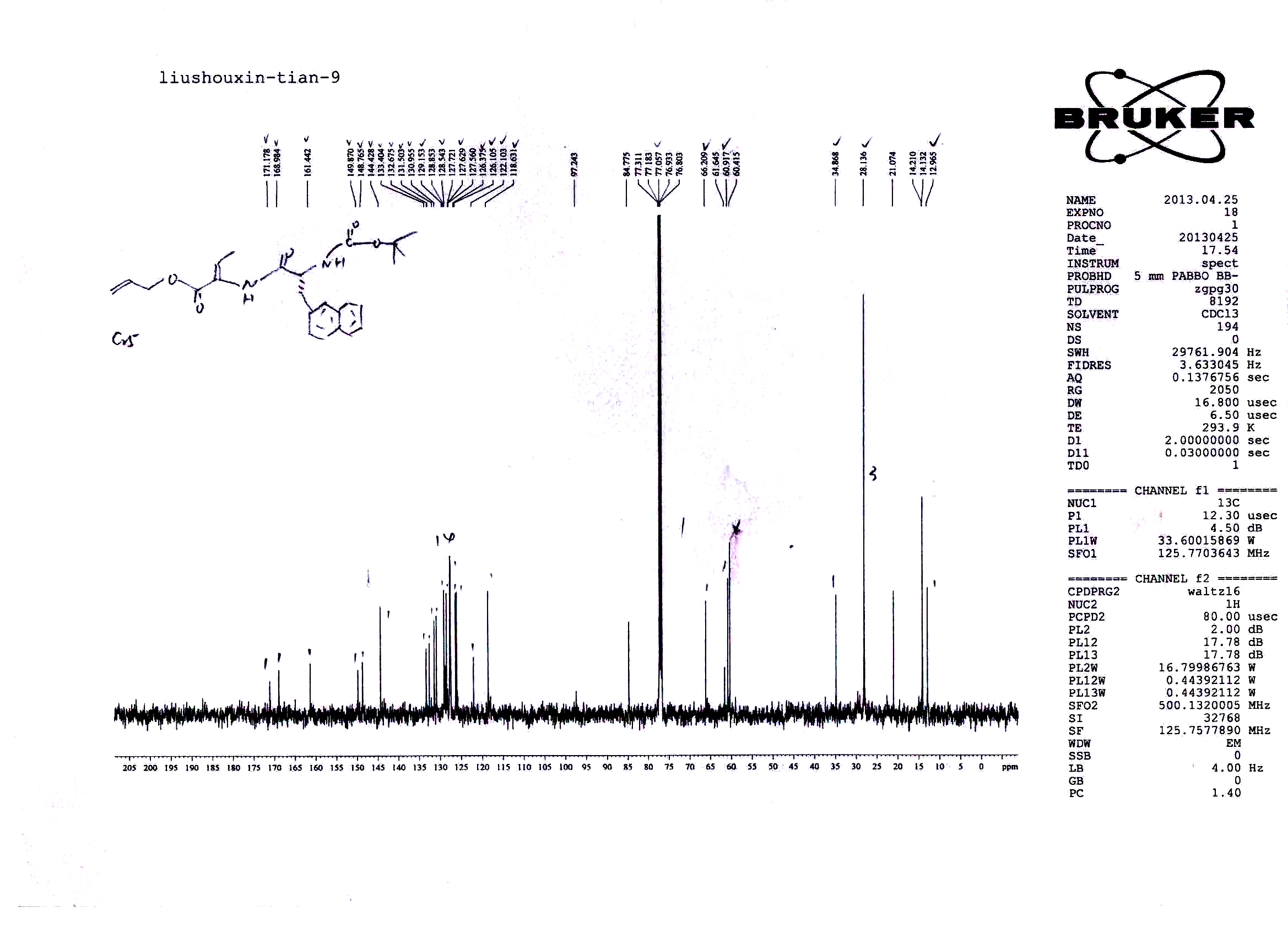
*

Figure S45 .CNMR spectrum of *Boc-D-β-Nap-Z-Δabu–Oallyl (****6l****)*
